# Chemical mapping of the surface interactome of PIEZO1 identifies CADM1 as a modulator of channel inactivation

**DOI:** 10.1073/pnas.2415934121

**Published:** 2024-10-02

**Authors:** Anna K. Koster, Oleg Yarishkin, Adrienne E. Dubin, Jennifer M. Kefauver, Ryan A. Pak, Benjamin F. Cravatt, Ardem Patapoutian

**Affiliations:** ^a^HHMI, Scripps Research, La Jolla CA 92037; ^b^Department of Neuroscience, Scripps Research, La Jolla, CA 92037; ^c^Department of Chemistry, Scripps Research, La Jolla, CA 92037

**Keywords:** PIEZO1, CADM1, mechanosensation, ion channel

## Abstract

The propeller-shaped blades of the PIEZO1 and PIEZO2 ion channels partition into the plasma membrane and respond to indentation or stretching of the lipid bilayer, thus converting mechanical forces into signals that can be interpreted by cells, in the form of calcium flux and changes in membrane potential. While PIEZO channels participate in diverse physiological processes, from sensing the shear stress of blood flow in the vasculature to detecting touch through mechanoreceptors in the skin, the molecular details that enable these mechanosensors to tune their responses over a vast dynamic range of forces remain largely uncharacterized. To survey the molecular landscape surrounding PIEZO channels at the cell surface, we employed a mass spectrometry-based proteomic approach to capture and identify extracellularly exposed proteins in the vicinity of PIEZO1. This PIEZO1-proximal interactome was enriched in surface proteins localized to cell junctions and signaling hubs within the plasma membrane. Functional screening of these interaction candidates by calcium imaging and electrophysiology in an overexpression system identified the adhesion molecule CADM1/SynCAM that slows the inactivation kinetics of PIEZO1 with little effect on PIEZO2. Conversely, we found that CADM1 knockdown accelerates inactivation of endogenous PIEZO1 in Neuro-2a cells. Systematic deletion of CADM1 domains indicates that the transmembrane region is critical for the observed effects on PIEZO1, suggesting that modulation of inactivation is mediated by interactions in or near the lipid bilayer.

## Significance

The process by which the body senses and responds to mechanical pressure (mechanosensation) is often carried out by PIEZO ion channels that reside in the plasma membrane. Local calcium flux through these force sensors is postulated to be upstream of many intracellular pathways that drive inherently mechanical processes. However, the mechanisms of signal transduction regulated by proteins surrounding PIEZOs at the cell surface have been difficult to study directly, as have the molecular details that govern detection of mechanical stimuli over many orders of magnitude within different tissues and organs. In this study, we aimed to capture a snapshot of the local endogenous protein network that may enable calibration of PIEZO-mediated mechanosensation within different cellular contexts.

Sensation of mechanical forces in our external and internal environments is such a routine part of existence that we often take it for granted—for instance, we know where our arms and legs are in relation to the rest of our body (proprioception), and we continually regulate blood pressure by sensing the force of blood flow in the vasculature. At the cellular level, each of these processes is mediated by PIEZO ion channels that reside in the plasma membrane and open in response to mechanical stimuli to permit the flow of calcium and other cations. This conversion of mechanical forces into electrochemical signals, or mechanotransduction, underlies how cells sense and respond to changes within their microenvironments. However, the protein ensembles and signaling pathways that collectively mediate these processes are not well defined at the molecular level due to a scarcity of tools to broadly study the PIEZO channel interactome and subcellular localization within different contexts.

Superresolution microscopy experiments using tagged PIEZO1 proteins have shown that although PIEZO1 freely diffuses within the plasma membrane, it dynamically localizes to sites of high cellular tension and traction forces where transient PIEZO-mediated Ca^2+^ flickering events have been observed (1–5). The downstream consequences of such Ca^2+^ events include calpain-associated proteolysis of focal adhesion proteins, which disrupts cellular attachment and facilitates alignment of vascular endothelial cells in the direction of applied shear stress (6). This is critical for vascular development in mammals—endothelial-specific *Piezo1* knockout in mice is embryonic lethal (6, 7). Such studies suggest that subcellular localization of PIEZO1 proteins within the plasma membrane is important for their physiological function and that such localization is not static. Elucidating the molecular neighborhood of proteins that may participate in PIEZO-initiated signaling pathways may provide insights into this dynamic molecular landscape.

A second motivation for more thoroughly characterizing the PIEZO1 interactome is the observation that endogenous PIEZO1-mediated currents in many primary cells and cell lines exhibit much slower inactivation than is observed in heterologous overexpression systems (8–15). Inactivation, defined as the reduction of channel open probability during sustained application of a mechanical stimulus (presumably as a result of conformational change into an inactivated state), is one of the most important mechanisms of physiological regulation of PIEZO channels, as evidenced by multiple PIEZO1 and PIEZO2 disease-causing point mutations that alter this parameter (16). In C2C12 and HEK293T cells, for example, endogenous PIEZO1-mediated currents do not readily inactivate. However, overexpression of PIEZO1 confers fast-inactivating currents upon these cells, suggesting that endogenous factors contribute to slowing of channel inactivation (9). Although several protein interaction partners for PIEZOs have been suggested in the literature (11, 15, 17–23), systematic evaluation of more extensive protein interaction networks has been technically challenging.

Driven by similar observations and hypotheses, a recent publication disclosed a structure of PIEZO1 intracellularly bound to a transcriptional regulator in the Myo-D (myoblast determination)-family inhibitor class that appears to serve as an auxiliary subunit of the channel and accounts for the noninactivating phenotype of PIEZO1 in many cell lines (21). This study utilized coimmunoprecipitation (co-IP) followed by mass spectrometry (MS) in a fibroblast cell line to identify intracellular MDFIC and MDFI as PIEZO1 interaction partners. In this study, we followed a different and complementary approach that allowed us to probe a broader protein network of PIEZO1 at the cell surface with high spatiotemporal resolution, leveraging covalent capture of adjacent proteins. We specifically used a modified extracellular proximity labeling approach, followed by multiplexed MS-based proteomics to generate a list of protein interaction candidates in HEK293 cells, which we then subjected to functional screening with calcium imaging and electrophysiology. We identified two proteins, CADM1 and GPC4, that modulate PIEZO1 function, and we chose to focus on one of these, CADM1/SynCAM. CADM1 is an adhesion molecule that has been independently characterized within three contexts: neuronal synaptic organization (24), tumor suppression in non–small cell lung cancer (25), and adhesion between spermatogenic and Sertoli cells (26). We found that CADM1 slows PIEZO1 inactivation and increases the open dwell time of the channel in single-channel recordings. Our studies provide a foundation for further exploration into how various proteins may modulate PIEZO channels in a physiological context and lay the groundwork for a unique tool to study diverse and dynamic interaction networks involving PIEZO proteins.

## Results

### Proximity Labeling with PIEZO1 at the Cell Surface.

Establishing a method to capture snapshots of the PIEZO1 protein interaction network may provide insight into the dynamic cellular pathways involved in mechanical signaling. However, mapping such protein networks at the plasma membrane presents technical challenges due to their low abundance and inherent hydrophobicity. Standard approaches to characterize protein–protein interactions, such as co-IP, are often not amenable to membrane proteins, as they require the use of detergents that may cause weak or transient interactions to fall apart before they can be detected. We chose to use a covalent proximity labeling approach, as it avoids some of these technical challenges and provides the opportunity to extract information on transient interactions that impact PIEZO1 activation.

As a starting point for our method development, we considered approaches that genetically tag a protein of interest (POI) with an enzyme capable of covalently appending biotin to nearby proteins within a narrow spatial radius. Captured proteins can then be pulled down with streptavidin, identified, and quantified in a multiplexed MS format through isobaric tagging of proteolytically digested peptide fragments (tandem mass tag (TMT)-based proteomics) (27). One such approach introduces an engineered ascorbate peroxidase (APEX2) (28) onto a POI, which generates a short-lived (<1 ms) biotin-phenol radical in the presence of hydrogen peroxide. This radical only diffuses a short distance (~20 nm) (29) from its source before colliding with nearby amino acids or being quenched by radical scavengers, allowing for the detection of dynamic changes in protein interaction networks with high temporal (<1 min) and spatial resolution.

**Fig. 1.**
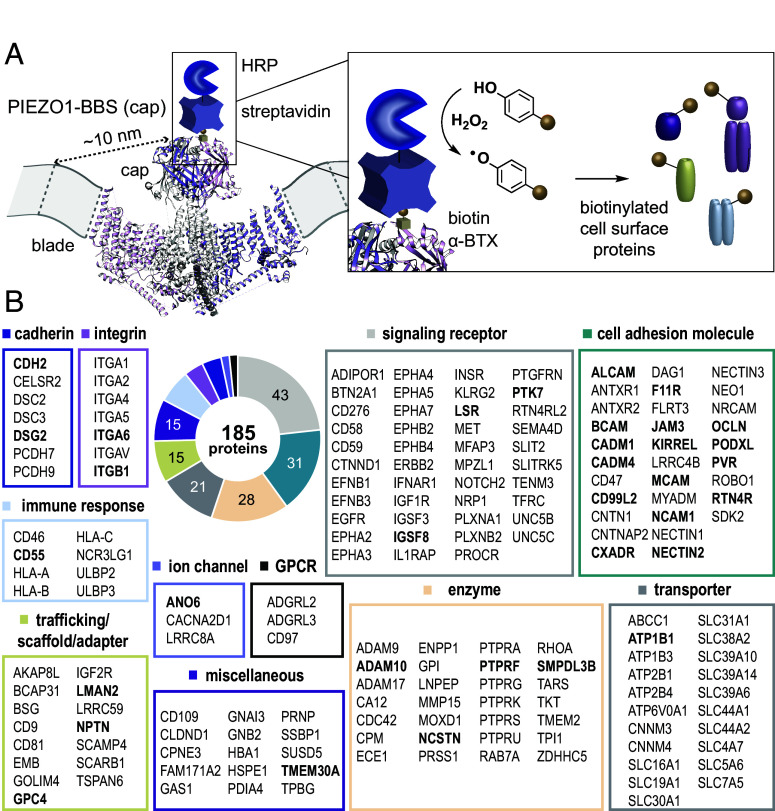
Proteomic mapping of the PIEZO1 surface interactome. (*A*) Side view of the PIEZO1 trimeric structure (PDB: 6BPZ) in the plasma membrane, illustrating the PIEZO1-BBS (cap) construct bound to a cartoon of α-BTX-biotin and a streptavidin-HRP conjugate for biotinylation of extracellularly exposed proteins within ~20 nm of the cap. The distance between the top of the cap domain and end of the extracellular distal blade is ~10 nm, based on other resolved structures (black dotted line). For clarity, the distal blade repeats are not well resolved in the 6BPZ structure and are represented by gray dotted lines within the plasma membrane. (*B*) Set of proteins found by proximity labeling and MS-based proteomics to reside in the vicinity of PIEZO1-BBS (cap), annotated by functional classifications. Bolded proteins were selected for follow-up functional screening (Fig. 2*A*).

To minimize the effects of intracellular protein aggregation and amplify the signal from PIEZO1 specifically on the cell surface, we chose to harness the properties of horseradish peroxidase (HRP), an enzyme that performs the same chemistry as APEX2 but is inactive in the reducing environment of the cytosol due to the presence of structurally essential disulfide bonds (29, 30). Rather than directing HRP to a POI with an antibody (31–36) or creating a genetically encoded fusion of HRP to an extracellular domain (37), we employed a 13-amino acid bungarotoxin binding site (BBS) tag inserted into the extracellular PIEZO1 “cap”(38), a domain which sits over a central ion conduction pore and that is formed by the convergence of three propeller-shaped “blade” subunits (39) (Fig. 1*A*). The BBS sequence is derived from the nicotinic acetylcholine receptor and binds α-bungarotoxin (α-BTX) tightly and stably with an IC_50_ of ~2 nM (40, 41). We then used commercially available α-BTX-biotin to direct a streptavidin-HRP conjugate to PIEZO1 on the cell surface with high affinity (Fig. 1*A*).

For our MS studies, we employed HEK293T cells, which we used because they endogenously express PIEZO1 at low levels and exhibit slowly inactivating endogenous PIEZO1 currents (15), making them suitable for identification of proteins that may be in close proximity to endogenous PIEZO1. Furthermore, HEK cells are easily transfected and can be grown on large scale, making them ideal for initial method development. Using the extracellular labeling approach described above, we found that biotinylation of neighboring proteins by PIEZO1-BBS was extremely clean with little to no MS signal originating from samples in which α-BTX-biotin was omitted or when PIEZO1-GFP was expressed without a BBS tag in the presence of all other labeling components (*SI Appendix*, Fig. S1*D*). We also found that labeling could be accomplished with equivalent efficiency by incubation with only 0.2 μg/mL (~25 nM) α-BTX-biotin compared to 10 μg/mL (~1.25 μM), allowing for cost-effective, large-scale labeling in 15-cm plates (*SI Appendix*, Fig. S1*D*). Preliminary gene ontology (GO) analysis (42, 43) also indicated that membrane proteins were being preferentially enriched. During method development, we also tried several other plasma membrane-localized controls, but it proved difficult to quantitatively interpret these data, in part due to the challenge of normalizing surface expression for these constructs (see details in *SI Appendix*, *Materials and Methods*). To circumvent these challenges, we ultimately chose to generate a PIEZO1-BBS (cap) stable cell line through lentiviral transduction (*SI Appendix*, Fig. S1 *A*–*C*) and performed identical proximity labeling experiments in a system with more uniform and reduced levels of PIEZO1 expression, closer to that of an endogenous system. For consideration as PIEZO1 interactors, we required proteins to have a spectral count >5, be present in all replicate experiments (9 total biological replicates performed for the BBS (cap) construct analyzed across four independent multiplexed TMT runs; n = 6 for transient overexpression, and n = 3 for the stable cell line), and be present in <25% of datasets in the CRAPome repository (44), a database of common and high-abundance contaminant proteins often found in affinity capture proteomics experiments (Fig. 1*B*). Based on these criteria, we generated a list of 185 proteins representing a potential PIEZO1 surface interactome (Fig. 1*B*). We avoided strict ranking of these proteins based on spectral counts, as this metric is dependent on three parameters that are not readily distinguishable: 1) proximity to PIEZO1, 2) abundance on the plasma membrane, and 3) exposure of the extracellular domains of adjacent proteins above the plasma membrane (i.e., more protein surface area accessible for reaction with a biotin-phenol radical).

Given that a goal of this study was to develop a method whereby transient changes in the PIEZO1 interactome under different force stimuli could be acutely captured, we also performed extracellular proximity labeling experiments in the presence and absence of the PIEZO1 agonist Yoda1. We chose a ~1-min timepoint for stimulation because Ca^2+^ imaging experiments show a peak Yoda1 response within this timeframe (45). In the PIEZO1-BBS (cap) stable HEK293 cell line, we found a global decrease in the extent of labeling across the PIEZO1 interactome when 10 μM of Yoda1 was present compared to a DMSO vehicle control (*SI Appendix*, Fig. S1*E*). Mildly decreased labeling in the presence of Yoda1 could be a reflection of PIEZO1 conformational changes, such as blade flattening and expansion (46), or other subtle changes in membrane properties or binding partner affinities. Although we did not capture any remarkable changes of proteins enriched by PIEZO1 in HEK293 cells as a result of Yoda1 stimulation on this timescale, these results provide a foundation for future experiments to quantitatively address molecular changes that occur in the vicinity of PIEZO1 under different types of mechanical stimuli applied over different timescales, perhaps in more physiologically relevant systems where the BBS tag has been knocked into the *PIEZO1* endogenous gene locus.

### Selection and Functional Testing of Potential PIEZO1 Interaction Candidates.

GO and literature analysis of the PIEZO1 interactome by cellular compartment and molecular function revealed that the majority (~90%) of captured proteins are plasma membrane associated. PANTHER classifications (47) of these proteins by primary function or protein class showed that signaling receptors and adhesion molecules were the most commonly captured (Fig. 1*B*), suggesting that PIEZO1 may be localized to intercellular signaling hubs and cell–cell or cell–substrate junctions, as has been suggested previously (1, 3, 5, 20, 22, 48, 49).

**Fig. 2.**
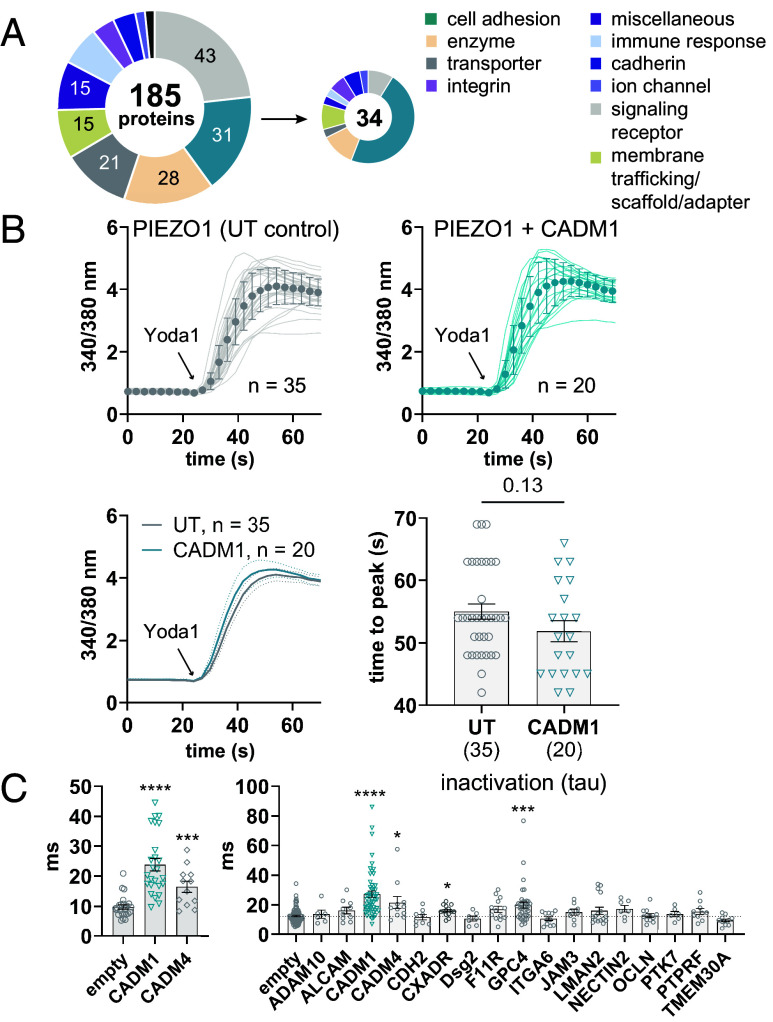
Functional screening of protein interaction candidates with PIEZO1. (*A*) Protein classifications of proteins selected for functional screening from the PIEZO1 interactome dataset. The 34 selected proteins in the pie chart are bolded in Fig. 1*B*. (*B*) *Top*: Representative calcium imaging data in mPIEZO1 stable HEK293F cells showing the left-shifted response to 5 μM Yoda1 in cells visibly transfected with CADM1 (turquoise) vs. untransfected (UT) cells (gray) in the same field of view (n = 20 and n = 35 cells, respectively). Markers show the average ± SD; *Bottom Left*: Average of raw traces shown at the top with dotted lines indicating the 90% CI. *Bottom Right*: Quantification of the time-to-peak Yoda1 response for the individual cells averaged in the left panel. (*C*) *Left*: PIEZO1 inactivation quantified by whole-cell poke in 3-G7 cells used for calcium imaging. Cells were transfected with an empty vector control, CADM1, or CADM4 (mean ± SEM, empty: 9.8 ± 0.7 ms, n = 24; CADM1: 24 ± 2 ms, n = 25, *P* < 0.0001 vs. empty; CADM4: 16 ± 2 ms, n = 12, *P* = 0.0003 vs. empty). One cell for CADM1 revealed little inactivation during the stimulus (>125 ms, no exponential could be fit) and was not included in the statistical analysis of tau. *Right*: Inactivation summary panel of 17 proteins screened by whole-cell poke electrophysiology in HEK293T PIEZO1 KO cells. Cells were cotransfected with mPIEZO1 and each untagged candidate protein. CADM1, CADM4, GPC4, and CXADR were statistically different from the control vector (*SI Appendix*, Table S1), with CADM1 and GPC4 showing the largest effects.

We selected a subset of representative proteins across classes and structural motifs known to be at cell interfaces for functional testing (Figs. 1*B* and 2*A*). For preliminary screening, we subcloned 34 genes into a mammalian expression vector with a C-terminal fusion mCherry fluorescent tag (*SI Appendix*, *Appendix II*) and tested 29 of these by ratiometric calcium imaging (the remaining 5 constructs did not express well or at all and were not tested, *SI Appendix*, *Appendix II and III*). We tested protein interaction candidates by transiently overexpressing each cDNA in a stable PIEZO1-GFP HEK293F cell line derived from a single-cell clone (*SI Appendix*, Fig. S1 *A* and *B*). In these cells, we looked for differences in the response of PIEZO1 to 5 μM of Yoda1 (Fig. 2*B* and *SI Appendix*, *Appendix III*). CADM1 (a single-pass adhesion molecule in the immunoglobulin superfamily) and GPC4 (a cell surface heparan sulfate proteoglycan, HSPG) were identified as preliminary hits, with CADM1 accelerating (Fig. 2*B*) and GPC4 suppressing (*SI Appendix*, Fig. S2*B*) the Yoda1-induced response. While these results were promising, we were concerned that the fusion of mCherry to the C terminus of our protein candidates might affect their folding, trafficking, function, and potential protein–protein interactions with PIEZO1, especially given their small size and important signaling domains that reside at both the C and N termini of such proteins. We also questioned whether PIEZO1 stimulation by Yoda1 was sensitive enough to detect all possible functional effects.

To test whether the C-terminal mCherry fusion might alter candidate functionality and to confirm our calcium imaging results, we selected 17 candidate proteins for additional testing by whole-cell patch clamp electrophysiology (Fig. 2*C* and *SI Appendix*, Table S1). We subcloned each gene into an IRES expression vector and tested the effect of coexpressing untagged proteins with PIEZO1 in our standard “poke” assay (50), a method of mechanically stimulating single cells by indenting the plasma membrane in half-micron increments while simultaneously recording their whole-cell currents. Due to the extremely low throughput of this assay, we tested only a small subset of genes, albeit quantitatively and with high precision. Gratifyingly, we found that the most active PIEZO1 modulators determined by calcium imaging, CADM1 and GPC4, were also the strongest candidates by electrophysiology (Fig. 2 *B* and *C*). We measured four parameters from the poking-induced whole-cell current families: 1) the inactivation time constant (tau), a measurement of how fast PIEZO1 macroscopic current decays during mechanical stimulation; 2) apparent maximal current (I_max_) at –80 mV; 3) percent of I_max_ remaining at the end of the stimulus; and 4) apparent threshold, the difference in indentation depth between the first probe contact with the cell (determined visually) and the first mechanical response (determined electrophysiologically).

**Fig. 3.**
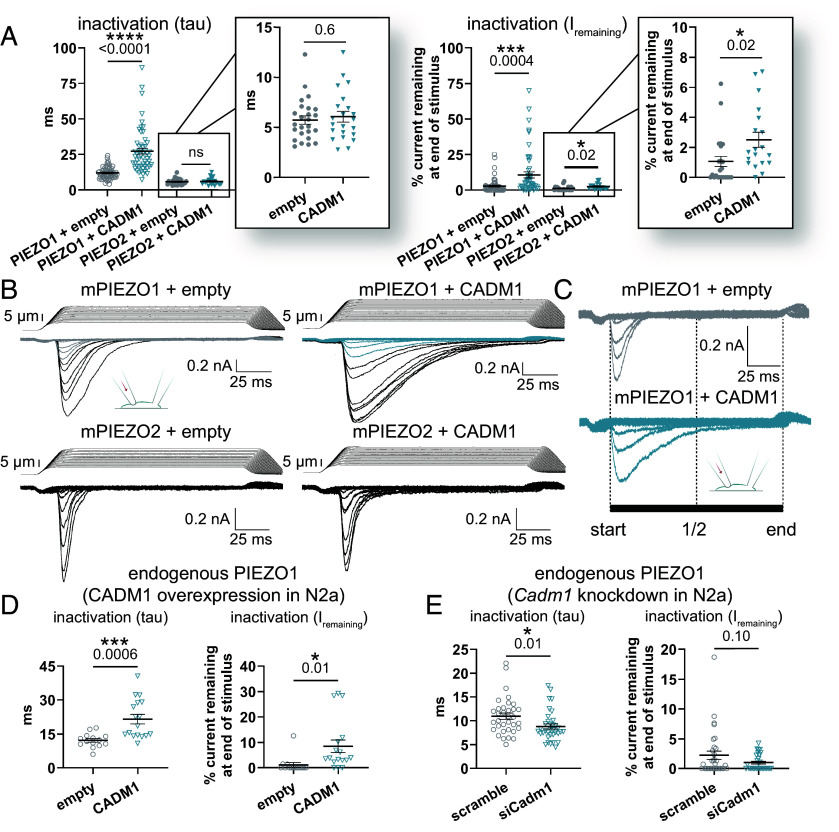
Electrophysiological characterization of the effect of CADM1 on PIEZO channels. (*A*) Comparison of the effects of CADM1 overexpression on mPIEZO1 and mPIEZO2 overexpressed in HEK293T PIEZO1 KO cells, using whole-cell poke for mechanical stimulation at –80 mV. *Left*: The inactivation kinetics of mPIEZO1 are slower with CADM1 compared to empty vector control (mean ± SEM: empty, 11.9 ± 0.5 ms, n = 62; CADM1, 27 ± 2 ms, n = 54; *P* < 0.0001) with little to no effect on mPIEZO2 (mean ± SEM: empty, 5.7 ± 0.4 ms, n = 25; CADM1, 6.1 ± 0.5 ms, n = 22; *P* = 0.6). *Right*: Percent of apparent maximal current (I_max_) remaining at the end of the mechanical stimulus is greater for mPIEZO1 with CADM1 compared to empty vector control (mean ± SEM: empty, 2.8 ± 0.7%, n = 58; CADM1, 11 ± 2%, n = 53; *P* = 0.0004) with a more subtle effect on mPIEZO2 (mean ± SEM: empty, 1.1 ± 0.3%, n = 23; CADM1, 2.5 ± 0.5%, n = 19; *P* = 0.02). For PIEZO1, data are pooled from eight independent transfections, and statistical significance is observed on each day when data are not pooled (*SI Appendix*, Fig. S3*A*). Data for PIEZO2 are pooled from four independent transfections. (*B*) Representative PIEZO1 (*Top*) and PIEZO2 (*Bottom*) traces for the data shown in panel *A* with and without CADM1 overexpression. (*C*) Zoomed in view of the currents elicited by the lowest intensity stimuli shown in panel *B* (gray = empty control, turquoise = CADM1), for clarity. (*D*) Summary data of whole-cell poke recordings of endogenous mPIEZO1 in N2a cells with transient overexpression of CADM1, showing slowed inactivation (*Left panel*, mean ± SEM: empty, 12.2 ± 0.8 ms, n = 14; CADM1, 22 ± 2 ms, n = 17; *P* = 0.0006) and increased I_max_ % remaining (*Right panel*, mean ± SEM: empty, 1.1 ± 0.9%, n = 14; CADM1, 9 ± 2%, n = 17; *P* = 0.01) in the presence of CADM1 that mirrors the effects seen with transient PIEZO1 overexpression in HEK cells (panel *A*). Data are pooled from three independent recording days. (*E*) Summary data of whole-cell poke recordings of endogenous mPIEZO1 in N2a cells with siRNA knockdown of endogenous CADM1, showing acceleration of channel inactivation in contrast to the slowing seen with overexpression (*Left*: nontargeting scramble control, 11.0 ± 0.7 ms, n = 34; si*Cadm1*, 8.8 ± 0.5 ms, n = 35; *P* = 0.01). Percent I_max_ remaining is not significantly different between conditions but trends in the opposite direction of overexpression (*Right*: nontargeting scramble control, 2.3 ± 0.7%, n = 32; si*Cadm1*, 1.0 ± 0.2%, n = 30; *P* = 0.1). Data are pooled from three independent transfections with the experimenter blinded to conditions.

Similar results were observed when our protein candidates were transiently overexpressed either in the PIEZO1-GFP stable HEK293F cells that we used for Ca^2+^ imaging or with overexpressed PIEZO1 in a cell line lacking endogenous PIEZO1-mediated currents [HEK293T PIEZO1 knockout (KO) cells (15)] (Fig. 2*C*). CADM1 slowed the rate of PIEZO1 inactivation more than twofold compared to vector controls recorded on the same day (Figs. 2*C* and 3*A* and *SI Appendix*, Table S1) and created a ~3-fold increase in the percentage of current remaining at the end of the mechanical stimulus (Fig. 3*A* and *SI Appendix*, Table S1). The slow inactivation phenotype of PIEZO1 in the presence of CADM1 was consistent, with a statistically significant difference in tau relative to control on all eight individual experiment days with different experimenters who were blinded to the conditions (*SI Appendix*, Fig. S3*A*). CADM1 had no effect on the apparent I_max_ and a minimal effect on the apparent mechanical threshold for the poke-induced response (*SI Appendix*, *Appendix I*). CADM1 also had no effect on threshold in cell-attached stretch electrophysiology recordings using high-speed pressure clamp (HSPC), a system to apply negative pressure through a recording pipette on the cell membrane that is considered the gold standard for evaluating mechanical sensitivity (*SI Appendix*, Fig. S3*B*). CADM1 is one of four closely related subtypes (CADM1–4) that engage in both homophilic and heterophilic interactions with each other through their extracellular immunoglobulin (Ig) domains. To evaluate whether other family members also modulate PIEZO1, we tested CADM4, which also appears in our MS datasets. We found that CADM4 produces a similar slow inactivation phenotype to CADM1 (Fig. 2*C*), preliminarily suggesting a conserved mechanism among CADM family members.

GPC4 had a significant but more variable effect on PIEZO1 inactivation, but, unlike CADM1, also showed significant effects on the apparent I_max_ and mechanical threshold of activation in poke experiments (Fig. 2*C* and *SI Appendix*, Fig. S2 and Tables S1 and S2). Glypicans (GPC1–6) are extracellularly facing HSPGs that are anchored to the cell surface through a glycosylphosphatidylinositol (GPI) linkage (51) (*SI Appendix*, Fig. S2). Interestingly, we found that all GPCs modulate PIEZO1 properties to varying degrees (*SI Appendix*, Fig. S2 andTable S2), but we also observed that glypican overexpression in HEK293T cells could cause distinct morphology changes, in particular, cellular flattening. Thus, the change in apparent mechanical threshold that we measure in poke experiments could be related to cytoskeletal reorganization and actin stress fiber formation, which has been reported to occur as a result of modified canonical and noncanonical Wnt signaling upon GPC4 upregulation or overexpression (52, 53). Puzzled by our seemingly contradictory electrophysiology and calcium imaging results for GPC4 (increased I_max_ but decreased Yoda1 response) and the possibility of structural cellular changes driving our observations, we decided to focus our attention on CADM1 for further characterization. In addition to CADM1, CADM4, and GPC4, a few proteins that we tested in our electrophysiology screen had subtle effects on PIEZO1 function that were not captured by our calcium imaging assay (*SI Appendix*, Table S1). CXADR was the only other protein to have a significant effect on inactivation, but this effect was small compared to CADM1. CXADR also significantly increased the apparent I_max_ of PIEZO1, as well as F11R and OCLN.

### Characterizing the Effects of CADM1 on PIEZO Ion Channels.

One way that cells and tissues are able to respond to a wide range of forces (both type and magnitude) is to express two PIEZO subtypes, PIEZO1 and PIEZO2, which share ~50% sequence identity and are differentially expressed in organ systems that are exposed to a variety of mechanical forces (16, 54). PIEZO1 is generally expressed in tissues that experience shear stress, such as in vascular endothelial cells (6, 7, 16, 55), whereas PIEZO2 is primarily expressed in sensory neurons and Merkel cells where it plays a role in touch sensation (16, 56–58). To test whether CADM1 universally modulates both channel subtypes, we overexpressed it together with PIEZO2 in HEK293T PIEZO1 KO cells (necessary to avoid potentially confounding effects from endogenous PIEZO1 in HEK293T cells). We found no significant change in mPIEZO2 inactivation kinetics by CADM1 coexpression and observed only a mild effect on the current remaining at the end of the mechanical stimulus. Overall, these results suggest that the effects of CADM1 on inactivation are somehow specific to PIEZO1 (Fig. 3 *A*–*C* and *SI Appendix*, Table S3). As an additional control, we coexpressed PIEZO1 and PIEZO2 with the recently reported auxiliary subunit, MDFI, which induces a noninactivating phenotype for both PIEZO family members (21). In contrast to CADM1, we confirmed the induction of noninactivating currents on both PIEZOs in the presence of MDFI (*SI Appendix*, Fig. S3*G*).

We next sought to determine whether CADM1 alters other functional characteristics of PIEZO1, such as voltage dependence of inactivation and single-channel properties. We found that CADM1-dependent slowing of inactivation appears to occur at both positive and negative holding potentials, suggesting that CADM1 does not influence regions contributing to voltage dependence (*SI Appendix*, Fig. S3*F*). The current-voltage relationship, reversal potential, and recovery from inactivation of PIEZO1 also remain apparently unaffected by the presence of CADM1 (*SI Appendix*, Fig. S3 *F* and *H*–*J*). In preliminary cell-attached recordings of overexpressed PIEZO1 with or without CADM1, we found no difference in the single-channel conductance (*SI Appendix*, Fig. S3, *C* and *D*); however, the open dwell time of PIEZO1 was nearly three times longer in cells coexpressing CADM1 (*SI Appendix*, Fig. S3*E*), consistent with the slowing of PIEZO1 inactivation observed in whole-cell recordings.

To better address the physiological relevance of the functional interaction between PIEZO1 and CADM1, we examined endogenous PIEZO1-mediated currents in Neuro-2a (N2a) cells, a mouse neuroblast cell line in which PIEZO1 was first discovered (9). Endogenous poke-induced currents in N2a cells are large and robust (~100 to 200 pA). While they are moderately fast inactivating, their mechanocurrents depend entirely upon *Piezo1* expression (9). Importantly, they also do not express MDFI or MDFIC, which induce slow-inactivating PIEZO1 currents (21). First, we transiently overexpressed CADM1 in N2a cells and recorded their poking-induced currents. We observed a slowly inactivating PIEZO1 phenotype nearly identical to that observed with overexpressed PIEZO1 in HEK293T PIEZO1 KO cells (Fig. 3*D* and *SI Appendix*, Table S4). This cell line also expresses endogenous CADM1, so we tested whether modulation of endogenous levels of CADM1 could influence PIEZO1 kinetics. We transiently knocked down endogenous *Cadm1* with ~62 to 70% bulk efficiency (evaluated by qRT-PCR; *SI Appendix*, Fig. S4) and performed blinded endogenous PIEZO1 poke recordings in cells transfected with either a noninactivating siRNA control or a pool of four siRNAs directed against *Cadm1*. A subtle but significant acceleration of endogenous PIEZO1 inactivation was observed for N2a cells with reduced levels of CADM1 (Fig. 3*E* and *SI Appendix*, Table S5). It is important to note that CADM4, another member of the CADM protein family that is capable of modulating PIEZO1 inactivation (Fig. 2*C*), is also present in N2a cells and may partially mask the effects of CADM1 knockdown. Interestingly, the current remaining at the end of the mechanical stimulus was also diminished in the CADM1-deficient N2a cells (Fig. 3*E*), which is opposite that seen with CADM1 overexpression (Fig. 3*D*). Our findings that overexpression and knockdown approaches produce opposing effects on endogenous PIEZO1 inactivation affirm the relevance of the overexpression system.

### Probing the Mechanism of PIEZO1 Modulation by CADM1.

**Fig. 4.**
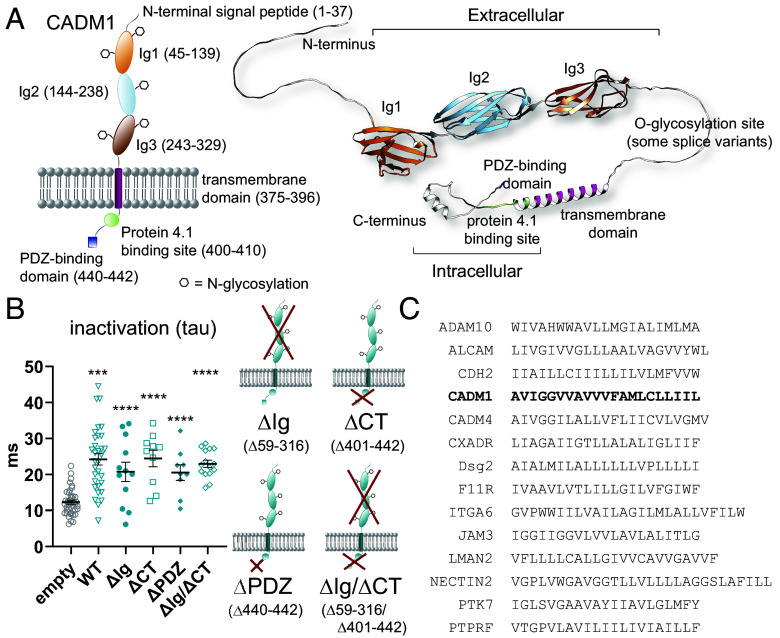
Mechanistic exploration of the effects of CADM1 on PIEZO1 inactivation. (*A*) Color-coded schematic of human CADM1 within the plasma membrane with the corresponding AlphaFold structure (AF-Q9BY67-F1) shown top right. Amino acid sequence numbers for the various domains are shown in parenthesis. (*B*) Comparison of the effects of deletion mutant CADM1 overexpression on mPIEZO1 overexpressed in HEK293T PIEZO1 KO cells, using whole-cell poke for mechanical stimulation (WT = wild-type CADM1; deleted residues for each mutant are listed in parenthesis in the cartoon shown to the right). All mutants slowed PIEZO1 inactivation similarly to WT CADM1 (mean ± SEM, tau: empty, 12.3 ± 0.6 ms, n = 40; CADM1 WT, 24 ± 2 ms, n = 32; ΔIg, 21 ± 3 ms, n = 12; ΔCT, 24 ± 2 ms, n = 10; ΔPDZ, 21 ± 2 ms, n = 9; ΔIg/ΔCT, 23 ± 1 ms, n = 15; *P* < 0.0001 relative to empty vector control for all conditions except ΔIg where *P* = 0.0005; also *SI Appendix*, Table S6). Data are pooled from 2 to 4 independent transfections and were compared to the empty vector control and WT CADM1 from the same day. (*C*) Comparison of the TM domain of single-pass membrane proteins that were screened by electrophysiology with CADM1 in bold. Only CADM1 and CADM4 are highly conserved, and both slow PIEZO1 inactivation (Fig. 2*C*).

Because of the diverse physiological roles that CADM1 plays, ranging from promoting cellular adhesion to directing intracellular signaling, we postulated that its ability to modulate PIEZO1 inactivation might directly involve the extracellular or intracellular domains that are functionally important in these processes. To delineate regions of CADM1 involved in modulating PIEZO1 inactivation, we systematically deleted each of these domains from human CADM1 and tested their effects on PIEZO1 by poke electrophysiology. The extracellular portion of CADM1 is composed of an N-terminal signal peptide that directs trafficking of the protein to the plasma membrane, followed by a triad of N-glycosylated Ig domains (24, 59), which are required for Ca^2+^-independent cellular adhesion (Fig. 4*A*). To evaluate whether the adhesive function of CADM1 is necessary to modulate PIEZO1, we deleted Ig domains 1 to 3, leaving the N-terminal signal peptide, transmembrane (TM) domain, and C terminus intact (ΔIg, Fig. 4*B*). The equivalent mutation of the mouse CADM1 homolog had previously been shown to localize normally to the plasma membrane but lacked the ability to associate with other CADM1 molecules (24). Surprisingly, we found that the extracellular Ig domains, which compose the largest region of the protein, were dispensable when it came to PIEZO1 modulation (Fig. 4*B* and *SI Appendix*, Table S6), suggesting that this process is independent of cellular adhesion through CADM1.

The short intracellular domain of CADM1 (CT, C-terminal) has an equally important but different function from the adhesion domains (60). It contains a PDZ binding motif, which interacts with MAGUKs (membrane-associated guanylate kinases) (61) (Fig. 4*A*), scaffolding proteins that interface between transmembrane proteins and intracellular signaling pathways and are essential for organization of both synapses (24, 62) and epithelial cells (polarity/morphology) (63–65). CADM1 also contains an intracellular protein 4.1 binding motif (66) (Fig. 4*A*) and may associate with intracellular protein 4.1 homologs, which participate in cytoskeletal organization (67). Modulation of PIEZO inactivation is multifaceted, and can depend on composition of membrane lipids (68–73) and structural elements, such as the cytoskeleton and extracellular matrix (ECM) (12, 22, 68, 74–77). As such, we hypothesized that CADM1 could modulate PIEZO1 inactivation through a direct interaction of the CT, structural effects (such as tethering to the cytoskeleton), or secondary effects related to signaling pathways triggered by the binding of various signaling molecules to the CT. To test these possibilities, we deleted the PDZ domain alone (ΔPDZ) or the entirety of the C terminus (ΔCT, Fig. 4*B*). We found that these mutants functioned equivalently to the wild-type protein to slow PIEZO1 inactivation (Fig. 4*B* and *SI Appendix*, Table S6), suggesting that secondary signaling pathways or cytoskeletal tethering are not required for the effects we observe. These results led us to hypothesize that PIEZO1 modulation by CADM1 is transmitted directly through the lipid bilayer, either through a direct interaction with the TM domain or by changing the local biophysical properties of the plasma membrane. To test this idea, we combined the ΔIg and ΔCT mutations into a single construct (ΔIg/ΔCT) and overexpressed the protein with PIEZO1 in HEK293T PIEZO1 KO cells. Remarkably, the ΔIg/ΔCT mutant slowed PIEZO1 inactivation equally to wild-type CADM1, even though ~70% of the protein is absent (Fig. 4*B* and *SI Appendix*, Table S6). The detailed mechanisms of possible amino acid-level interactions between PIEZO1 and CADM1 would require further investigation and structural characterization beyond the scope of this work.

## Discussion

Here, we employed a proximity labeling technique to globally survey the surface protein interactome of PIEZO1. We subsequently evaluated proteins in this interactome using calcium imaging and electrophysiology, uncovering two families of proteins, cell adhesion molecules (CADMs) and glypicans (GPCs), that modulate the activity of overexpressed PIEZO1 in HEK293T cells. We show that members from both families of proteins slow PIEZO1 channel inactivation in a standard poking assay and that the slowing in the presence of CADM1/SynCAM is consistent with an increase in channel open dwell time in preliminary cell-attached stretch recordings. Furthermore, overexpression of CADM1 in N2a cells slows endogenous PIEZO1 inactivation, while knockdown of endogenous CADM1 in the same cell line produces the opposite effect (accelerated inactivation), suggesting that this interaction may be physiologically important. The degree of modulation of PIEZO1 inactivation is within that observed by pathological mutations (16), indicating the potential of CADM1 and GPCs to alter PIEZO1-mediated physiologies within certain cellular contexts, in particular when they may become up-regulated.

Deletional mutagenesis studies on CADM1 revealed that neither the Ig extracellular domain nor the intracellular domains containing motifs for cellular signaling and cytoskeletal tethering are required to slow inactivation, suggesting the transmembrane domain and/or the remaining ~20 to 30% of extracellular amino acids are sufficient for PIEZO1 modulation. Our ΔIg/ΔCT mutant retains small parts of the wild-type sequence besides the TM domain: the signal peptide, two fragments of the proximal and distal ends of Ig1 and Ig3, and the membrane-proximal O-glycosylation site (*SI Appendix*, Fig. S5). The signal peptide that remains in the ΔIg/ΔCT CADM1 mutant is not well-conserved within the CADM family and is typically cleaved during or following translocation of single-pass membrane proteins to their respective subcellular compartments. Therefore, this sequence is not likely to be mechanistically important. Similarly, the O-glycosylation site is variable or missing in CADM1 splice variants and in CADM4 (59) (*SI Appendix*, Fig. S5), which is capable of modifying PIEZO1 inactivation (Fig. 2*C*). Given the high sequence conservation of the TM domain between CADM1 and CADM4 (Fig. 4*C* and *SI Appendix*, Fig. S5), it seems likely that this region is the most substantial contributor to PIEZO1 modulation. We also examined the TM domain sequences across the other protein candidates that we tested, none of which were notably similar to CADM1 (Fig. 4*C*). CXADR, the only other protein candidate to have a minor but significant effect on PIEZO1 inactivation, does not share any obvious TM sequence similarity with CADM1, which suggests a different mechanism.

The multiple functions of TM domains have been the most systematically studied in the context of chimeric antigen receptor T (CAR-T) engineering, a field dedicated to the modular design of synthetic receptors that are expressed in T lymphocytes for the purpose of recognizing and killing cancer cells. The choice of the TM domain in CARs can dramatically change CAR expression levels, stability, and activity, in part by affecting their propensity to dimerize, oligomerize, and engage with the TM domains of endogenous membrane proteins (78–80). The rules governing these types of intramembrane interactions are still being unraveled but often involve particular amino acid motifs (81–84). Glycines spaced at regular intervals, for example, can provide a flat surface to allow for tight helix packing and hydrogen bonding of the peptide backbone while exposing more hydrophobic sidechains at the lipid interface (85–88). A proteome-wide alignment of single-pass TM domains clustered CADM1 and its family members into a group of 11 proteins that share the sequence AviGGvia (capitalized letters are >90% conserved) (81). Interestingly, other members in this cluster are known to associate with or modulate ion channels, including the contactin-associated protein-like (CNTNAP) family, which associates with voltage-gated potassium channels (89), and SCN4B, a β subunit of voltage-gated sodium channels that modulates their gating and kinetics (90).

Our finding that CADM1 exerts little if any effect on PIEZO2 suggests that this is not a bulk effect on membrane properties, which might be expected to affect both PIEZO1 and PIEZO2. We do not altogether exclude this possibility, however, as PIEZO1 inactivation mechanisms are complex and are influenced by both intrinsic structural properties of the channel and extrinsic factors, such as voltage, temperature, lipid composition, and protein–protein interactions (91). PIEZO1 voltage dependence of inactivation relies on a pair of positively charged residues lining the channel pore (92, 93). Although other mutations affecting inactivation have been found throughout the channel, including throughout the blades, many functionally important residues are clustered at the intracellular C terminus near the pore (94, 95) and in the extracellular cap domain (38, 92, 96). Unaltered current-voltage relationships of poke-induced PIEZO1 whole-cell currents, the reversal potential, and single-channel conductance do not support a direct interaction of CADM1 with the channel pore, although this cannot be completely ruled out. The cap domain appears to be the primary contributor to the difference in inactivation kinetics between PIEZO1 and PIEZO2 (92, 96). As such, the interface between the cap domain and the extracellular side of the pore near the plasma membrane could be an interesting region to explore in terms of the enhanced ability of CADM1 to change the inactivation kinetics of PIEZO1 but not PIEZO2.

Physiological functions and expression patterns of PIEZO1 and CADM1/GPCs point to several intersecting and mechanically sensitive cellular pathways. GPCs are broadly involved in developmental morphogenesis and in directing tissue/synapse organization through the Wnt, Hedgehog, and other signaling pathways (51–53, 97). In fact, there are numerous intersecting roles of GPCs and PIEZO1 in tissue development (16) and in cancer (98, 99). As glypicans are components of the ECM (100), they may play a structural role in mechanosensing by changing the biomechanical properties of this network (77). For instance, PIEZO1 is upstream of shear-stress-induced nitric oxide (NO) production in adult vasculature (55). Degradation of various sugars that compose the endothelial glycocalyx (101–103), as well as knockdown or knockout of GPC1 (the primary glypican in the vasculature) (104, 105), impairs NO production in response to shear stress, suggesting a possible contribution of GPC1 in modulating the vascular PIEZO1 shear stress response.

CADM1 expression is similarly developmentally regulated or changed under pathological conditions [e.g., cancer (106)] that involve cellular remodeling. CADM1 is expressed in developing bone osteoblasts (107), a cell type where PIEZO1 also plays a role in cellular differentiation and bone growth in response to mechanical loading (108, 109). CADM1 protein expression has also been demonstrated in endothelial and smooth muscle cells throughout the human macro- and microvascular systems (110), and single-nucleotide polymorphisms in regulatory and intronic regions of the CADM1 gene have been associated with risk of venous thrombosis (111). CADM1 is additionally expressed in epithelial cells [particularly in lung alveolar cells (112) and cells of the developing bile duct (113)], pancreatic islet cells (114–118), mast cells (119, 120), epidermal stem cells (121), and some macrophages (115). Within some of these settings, CADM1 appears to regulate neuroendocrine and immune functions by facilitating cellular attachment and hormone secretion that happens at the axis between peripheral nerves and resident immune or islet cells. Both CADM1 and PIEZO1 are proposed to participate in glucagon and insulin secretion from pancreatic α and β islet cells, respectively (116, 118, 122–124). The role of CADM1 and other SynCAM family members in axonal pathfinding (125), including in the periphery, also led us to survey coexpression of CADM1 with PIEZOs in the sensory ganglia (126, 127). CADM1 is broadly expressed in most sensory neurons, including populations where PIEZO1 is expressed. PIEZO1 and CADM1 also appear to be coexpressed in putative baroreceptors (127), together with PIEZO2, where both channels are involved in acute blood pressure regulation (128). Based on our findings, expression or upregulation of CADM1 within these contexts may provide a molecular tuning mechanism for PIEZO1-mediated mechanotransduction by slowing its inactivation kinetics and, in essence, creating a transient and reversible PIEZO1 “gain-of-function” phenotype. Because PIEZO mutations that create similar electrophysiological signatures have direct consequences on human physiology and disease, we believe that modulation of inactivation by CADM1 could have important physiological outcomes within certain biological contexts and is an interesting area for future work.

## Materials and Methods

### Cell Culture.

HEK293F (FreeStyle™, Thermo Fisher, Cat#R79007), HEK293T (ATCC CRL-3216, Lot# 70023985), and HEK293T PIEZO1 KO (15) (ATCC, Cat#CRL-3519) human embryonic kidney cells were cultured in Dulbecco’s Modified Eagle Medium (DMEM, Gibco Cat#11995-065) supplemented with 10% v/v heat-inactivated fetal bovine serum (HI-FBS, Gibco, Cat#10082-147) and 1 × penicillin/streptomycin (Pen-Strep, Gibco Cat#15-140-122). Cells were used between passages 3 and 13. Neuro-2a (N2a, mouse neuroblastoma, ATCC, CCL-131, Lot# 70025321) were cultured in Eagle’s Minimum Essential Medium (EMEM, Cytiva, SH30024.FS) with Earle’s Balanced Salt Solution (EBSS) and L-glutamine additives and supplemented with 10% HI-FBS and 1x Pen-Strep.

### Molecular Biology: PIEZO Constructs.

*Piezo1*-BBS-2422-IRES-GFP [mPIEZO1-BBS (cap)], *Piezo1*-BBS86-IRES-GFP [mPIEZO1-BBS (blade)], and human K_v_1.2-BBS-S1-S2-IRES-GFP were used as characterized by Wu et al. (38). All electrophysiology experiments were performed with the mouse *Piezo1*-IRES-GFP construct (Uniprot ID: E2JF22) in pcDNA3.1 or human codon-optimized mouse *Piezo2*-IRES-mNeonGreen (Uniprot ID: Q8CD54-1) in pcDNA3.2. Mouse PIEZO1 with a GFP-FLAG tag linked to the C terminus in pcDNA3.1 (129) was used as a BBS tag-free control for proteomics experiments.

### Molecular Biology: Subcloning of cDNA Library.

Coding gene sequences were obtained from the Dharmacon CCSB human ORFeome or Mammalian Gene Collection libraries (Horizon Discovery). Protein sequences of all genes tested, as well as their sources and catalog numbers are provided in *SI Appendix, Appendix II*. If purchased cDNA sequences contained mutations differing from the canonical sequence reported in UniProt, they have been annotated in *SI Appendix, Appendix II*. In some cases, genes were corrected to reflect the canonical sequence using the Q5® Site-directed mutagenesis kit (New England Biolabs, Cat#E0552S). Detailed subcloning information is provided in *SI Appendix*, *Supporting Methods*.

### HEK293F mPIEZO1-GFP-FLAG Stable Cell Line Generation.

mPIEZO or mPIEZO1-BBS (cap) were fused to a C-terminal GFP-FLAG tag (see “PIEZO constructs”) and subcloned into a lentiviral vector. HEK293F cells were transduced with high-titer lentivirus. GFP-positive cells were fluorescence-activated cell sorting (FACS)-selected twice sequentially, yielding a heterogeneous population with ~75% GFP-positive cells. Correct protein folding was assessed by fluorescence-detection size exclusion chromatography, and plasma membrane expression of the BBS construct was validated by FACS live labeling with α-BTX conjugated to Alexa Fluor 647 (Invitrogen, Cat#B35450). Both cell lines were subjected to FACS single-cell sorting into 96-well plates, using the top 5% or 15% GFP fluorescence as a cutoff. Surviving single-cell clones were expanded and cryopreserved. Clonal lines were evaluated by calcium imaging with Yoda1 to select cells with the most homogeneous calcium response (*SI Appendix*, Fig. S1*A*). These lines were additionally characterized by western blot using an anti-FLAG antibody and by whole-cell electrophysiology (*SI Appendix*, Fig. S1*B*). The PIEZO1-BBS (cap) lines were characterized by streptavidin-HRP blot following cell-surface proximity biotinylation, and clone 4-F7 was used for MS-based proximity labeling (*SI Appendix*, Fig. S1 *A* and *C*). The PIEZO1-GFP-FLAG clone 3-G7 was used for all calcium imaging experiments and for preliminary screening of candidate genes by electrophysiology (*SI Appendix*, Fig. S1 *A* and *B*).

### Proximity Labeling.

HEK293T cells were plated a day before transfection in 15-cm plates (GenClone, Cat. #25-203). After adhering for 6 to 24 h, cells were transfected at 30 to 50% confluency in a 3:1 ratio PEI MAX (Linear Polyethylenimine Hydrochloride, Cat#24765-1, Polysciences Inc., Lot# A777707) to plasmid DNA. For a 15-cm plate, 10.2 µg of *Piezo1*-BBS2422-GFP, *Piezo1*-BBS86-GFP, *Piezo1*-GFP-FLAG, or Kv1.2-BBS-S1-S2 were transfected (with 30.6 µg PEI MAX in a 1 µg/µL stock diluted into in 2.5 mL serum-free DMEM). This mixture was added into the 22 mL of media already in the dish from the previous day. Cells reached ~90% confluency for proximity labeling. Proximity labeling was performed 48 h after transfection. For experiments with the HEK293F mPIEZO1-GFP stable cell line, cells were plated in 15-cm plates the day before experiments such that ~90% confluency was achieved on the day of labeling. Proximity labeling was performed according to a protocol adapted from ref. 130 with modifications described in *SI Appendix*, *Supporting Methods*. Briefly, α-BTX-biotin and streptavidin-HRP were secured to PIEZO1 on the cell surface during two incubation steps, followed by biotinylation of nearby proteins with biotin-phenol in the presence of hydrogen peroxide.

### Mass Spectrometry (*SI Appendix*, *Supporting Methods* for additional details).

***Protein precipitation.*** Cell pellets collected from proximity labeling were lysed by probe sonication in ice-cold DPBS (Gibco, Cat#14190-136) containing protease inhibitors (Roche, cOmplete ULTRA tablets, EDTA-free, Cat#5892791001). Protein concentrations were quantified by DC absorbance assay (BioRAD Cat#5000112) and diluted to a final concentration of 3 mg/mL in 500 μL. Protein was precipitated by sequential addition and mixing of ice-cold methanol (2 mL), chloroform (0.5 mL), and DPBS (1 mL) followed by vortexing for 30 s and centrifugation at 5,000 rpm for 10 min. The resulting protein disk was washed with 2 × 10 mL of ice-cold methanol. The supernatant was removed, and the pellet was allowed to air dry. Pellets were stored at –80 °C prior to subsequent steps.

***Denaturation, reduction, and alkylation.*** Each protein pellet was denatured by adding 500 μL of 6 M urea in DPBS, followed by 10 μL of 10% w/v sodium dodecyl sulfate (SDS) in DPBS. To reduce the protein sample, a solution containing equal volumes of 200 mM tris(2-carboxyethyl)phosphine hydrochloride (TCEP) in DPBS and 600 mM potassium carbonate in DPBS was prepared, and 50 μL of this mixture was added to the denatured sample. Each sample was probe-sonicated 15× and placed in a 37 °C shaker for 30 min, followed by alkylation with iodoacetamide (70 μL of 400 mM in DPBS) in the dark at room temperature for 30 min. An additional 130 μL of 10% SDS in DPBS was added to each sample, then diluted with 5.5 mL of DPBS (~0.5 M urea, 0.2% w/v SDS).

***Streptavidin pull-down and tryptic digest.*** Biotinylated proteins were pulled down with streptavidin agarose (100 μL per sample, Thermo Scientific, Cat#20353), according to manufacturer instructions. Beads were pelleted, washed, and resuspended in 200 mM EPPS buffer (pH 8.0 with NaOH). A 20-μg vial of sequencing-grade trypsin (Promega, Cat# V5111) was dissolved in 2.05 mL of 2 M urea in 200 mM EPPS buffer (pH 8.0). Calcium chloride (100 mM in 200 mM EPPS buffer, pH 8.0) was diluted 1:100 into the trypsin-containing solution and mixed by pipetting. On-bead tryptic digest was performed by adding 200 μL of this trypsin solution to each protein sample and incubating for ~14 h on a 37 °C shaker.

***TMT labeling and fractionation of peptides.*** Beads were removed from tryptic peptides with a micro-Bio-Spin chromatography column (Bio-Rad, Cat#7326204). TMT labeling of eluted peptides was performed, as previously described (131). Labeled peptides were combined and dried in a SpeedVac concentrator overnight. The resulting peptide/urea pellets were redissolved in Buffer A (95% H_2_O, 5% acetonitrile, 0.1% formic acid) and water-bath sonicated for 10 min. Samples were recombined into a single tube followed by addition of formic acid to reach a pH of 1 to 3. The peptide sample was fractionated offline on a Pierce™ high pH reversed-phase peptide fractionation column (Thermo Scientific Cat#84868), using a gradient elution of acetonitrile in 10 mM aqueous ammonium bicarbonate (131). Every third fraction was recombined into a 1.5-mL LoBind Eppendorf tube for a total of 3 fractions and dried overnight in a SpeedVac. Samples were resuspended in Buffer A by bath sonication and analyzed on an Orbitrap Fusion mass spectrometer (Thermo Scientific). Data acquisition was by an MS3-based TMT method as previously described (131).

***Data analysis.*** The Integrated Proteomics Pipeline (IP2) was used for raw data analysis. MS2 and MS3 spectra were analyzed using the ProLucid search algorithm against a nonredundant variant of the human UniProt database (release-2012_11). Residue modifications in the search algorithm were static modification of cysteine for carboxyamidomethylation (+57.02146 Da), static modification of lysine and the N terminus corresponding to a TMT (+229.162932 Da), and differential modification of methionine, accounting for oxidation (+15.9949 Da). At least two unique peptides were required for identification with a false discovery rate set at 0.01 and a spectral count (SC) cutoff of 5. Biological replicates of comparison groups were run for internal TMT comparison and were also repeated in separate TMT runs to ensure reproducibility of results. In total, the dataset represents 9 biological sample replicates for PIEZO1-BBS (cap) analyzed from the four independent TMT runs. Proteins not contained in all 4 TMT datasets were removed from the final list of proteins in the PIEZO1 interactome. Proteins were further ranked by comparing each dataset to the CRAPome (44), giving priority to proteins found in ≤25% (179/716) of datasets and thereby removing contaminants and highly abundant proteins commonly found in biotinylation datasets. GO analysis and manual annotation of the removed proteins based on the Human Protein Atlas (132) showed that the majority of these are annotated as intracellular or cytosolic, likely captured from dead cells (*SI Appendix*, *Supplementary File 2*). To evaluate background labeling, datasets were cross-referenced to TMT channels in which all labeling and biotinylation reagents were added to cells expressing mPIEZO1-GFP without a BBS epitope tag. Proteins equally enriched by PIEZO1 lacking a BBS tag were removed from consideration and included only a small set of biotin-associated proteins and keratin, which were also eliminated by our CRAPome filter.

### Calcium Imaging Screen.

Experiments were conducted in the HEK293F mPIEZO1-GFP clone 3-G7 stable cell line. Cells were plated 48 h after transfection with PEI MAX analogous to the protocol described for proteomics experiments (250 ng cDNA per well of a 12-well plate). Cells were plated on the morning of the experiment in imaging chambers (Ibidi, Cat#80804) and allowed to adhere for ~2 h prior to loading. Cells were washed 2× with a calcium Ringer’s solution (127 mM NaCl, 3 mM KCl, 10 mM HEPES, 2.5 mM CaCl_2_, 1 mM MgCl_2_, and 10 mM D-(+)-glucose, pH 7.3 with NaOH, 300-310 mOsm/kg with D-mannitol) and loaded at room temperature for 40 min. with 1 μM Fura2-AM (Life Technologies, Cat#F1201) and 0.02% w/v Pluronic F-127 (Invitrogen, Cat#P6867). Prior to recording, green cells (expressing PIEZO1-GFP) were identified, and a red filter was used to determine which of these expressed red fluorescence (expressing candidate gene). Data were acquired every 3 s using MetaFluor (v.7.8.2.0) as a ratio of 340/380 nm. Following 30 s of baseline recording, Yoda1 (10 μM, 2× solution; TOCRIS, Cat.#558610, CAS#448947-81-7) or DMSO control solution was rapidly pipetted near the corner of the imaging well to mix with the solution in the chamber. Ionomycin (Sigma-Aldrich, Cat#I0634) was added at the end of each experiment to identify viable cells. Data from cells lacking detectable red fluorescence were compared to cells with red fluorescence within the same field of view in an individual well (also *SI Appendix*, *Supporting Methods*).

### Electrophysiology.

#### Whole-cell patch clamp (poke) recordings.

HEK293T PIEZO1 KO cells were plated ~24 h before transfection in a 12-well plate. Cells were transfected at 40 to 50% confluency using ~500 ng of *Piezo1*-IRES-GFP or *Piezo2*-IRES-mNeonGreen in a 2:1 ratio of candidate gene/*Piezo1* subunit based on the plasmid molecular weight for each candidate construct (contained in an IRES-mCherry or IRES-tdTomato expression vector, as described in “molecular biology”). Lipofectamine™ 2000 (Invitrogen, Cat#11668027) was used to carry out transfections, using 1 μL per well in 100 μL of Opti-MEM™ (Gibco, Cat#31985062), according to manufacturer instructions. Cells were replated onto poly-D-lysine-coated glass coverslips (Corning Cat#354086 or VWR Cat#GG-12-PDL) ~24 h after transfection, a day before recording. Cells with red fluorescence (candidate gene expression) 48 h after transfection were recorded, as previously described (9) (also *SI Appendix*, *Supporting Methods*). Briefly, cells were held at –80 mV and mechanically stimulated in 0.5 μm increments every 10 s, using a glass probe polished to a 3 to 4 μm diameter (Sutter Cat#B150-110-10) (9). Electrode resistances ranged from 2 to 6 MΩ. Currents were sampled at 20 kHz and filtered at 10 kHz. All recordings were conducted at ambient temperature. Statistical *P*-values were calculated from an unpaired two-tailed *t* test comparing cells transfected with *Piezo1* or *Piezo2* and a candidate gene or an empty control vector containing a fluorescent marker.

To quantify recovery from inactivation, a two-step protocol was used in which a 100-ms mechanical stimulus was followed by a second 100-ms mechanical stimulus. The interstimulus interval varied from 10 ms to 30 s. To assess voltage dependence of inactivation, we used a 500-ms indentation with voltage steps from –80 to +80 mV (+20 mV increments). Voltage steps preceded the indentation protocol by 220 ms to allow the membrane capacitance current to decay before mechanical responses were elicited. The probe was initially positioned at ~2 to 4 µm from the cell and advanced at 1 µm/ms in 1 µm increments. For both stimulus paradigms, the step increment was stopped after eliciting a current of >200 pA and the last indentation depth maintained for subsequent protocols. Recovery time constants were obtained by fitting a built-in logistic function (OriginPro10) to the experimental data.

#### Cell-attached patch-clamp recordings (macroscopic currents) and analysis

Cells were transfected as described for “whole-cell recordings,” and experiments were performed 72 h after transfection. Electrodes were pulled as described above with resistance from 1 to 2.5 MΩ. Currents were sampled at 5 kHz and filtered at 2 kHz, using an 8-pole Bessel filter. Recordings were performed with HSPC, as described previously (9) (also *SI Appendix*, *Supporting Methods*).

#### Single-channel recording and analysis.

Transfection conditions, solutions, and equipment were identical to those described for macroscopic cell-attached recordings. Electrodes had a resistance of 2.5 to 4.5 MΩ and were used without fire-polishing. A positive pressure prepulse of +5 mmHg (2 s) was applied followed by a prestep to 0 mm Hg (100 ms) and a 500-ms step to –20 mmHg. Currents were sampled at 5 kHz and filtered at 2 kHz using an 8-pole Bessel filter. The holding potential was –40 mV. For calculation of unitary current, the holding potential was stepped from –120 to –40 mV (+20 mV increments) during negative pressure steps to –20 mmHg. Single-channel current amplitude at each voltage step was calculated using a Gaussian fit to the all-points amplitude histograms. A slope conductance was calculated from a linear regression fit to single-channel current-voltage plots. Single-channel open dwell time was analyzed at –80 mV. The open dwell-time was calculated using an exponential log probability fit to logarithmically transformed open dwell-time distributions plotted at 7 bins/decade. Statistical significance between the dwell-time distributions was assessed using the two-sample Kolmogorov–Smirnov test.

### CADM1 Knockdown and Overexpression Experiments in N2a Cells.

#### Cell culture and electrophysiology.

N2a cells (1 to 2 × 10^4^ cells/well) were plated in a 24-well plate containing 500 μL of media 15 to 24 h prior to transfection with Lipofectamine 2000. Each well was transfected with 5 pmol of SMARTpool *Cadm1* siRNA (Horizon Discovery, Cat#L-065464-01-0005) or ON-TARGETplus nontargeting control siRNA (Horizon Discovery, Cat#D001810-10-05, pool or single siRNA#1), 50 ng of an mCherry cDNA vector used as a fluorescent marker for transfected cells, and 0.25 μL of Lipofectamine 2000 in 50 μL of Opti-MEM. Medium was changed 3 d after transfection, and the cells were transfected a second time with identical conditions. Cells were used for electrophysiology or collected for qRT-PCR (~5 × 10^5^ cells) 3 d after the second transfection. For all N2a electrophysiology experiments, cells were transfected directly on poly-D-lysine coated coverslips and were not replated following transfection. Data acquisition and analysis for knockdown electrophysiology experiments was performed with the experimenter blinded to the identity of the conditions. For electrophysiology overexpression experiments, N2a cells were transfected with 40 to 45 ng of empty fluorescent vector control or CADM1-IRES-tdTomato cDNA in a 24-well plate and recorded 48 h posttransfection on 3 separate days. Electrophysiology solutions and protocols were identical to those described for whole-cell recordings. Knockdown efficiency was quantified by qPCR, as described in *SI Appendix*, *Supporting Methods*.

## Supplementary Material

Appendix 01 (PDF)

Dataset S01 (XLSX)

## Data Availability

Raw data for TMT mass spectrometry experiments have been deposited in the ProteomeXchange Consortium (133) via the PRIDE partner repository (134): Accession No. PXD054618. Summary data are provided in *SI Appendix, Supplementary File 2*. File 1: *SI Appendix*. File 2: PIEZO1 interactome proteomics data summary spreadsheet. All study data are included in the article and/or supporting information.
