# Supplementary material for: Chemical mapping of the surface interactome of PIEZO1 identifies CADM1 as a modulator of channel inactivation
Source: Proc Natl Acad Sci U S A. 2024 Oct 2;121(41):e2415934121. doi: 10.1073/pnas.2415934121 (PMC11474052; doi:10.1073/pnas.2415934121)
Supplement: Supplementary file 1 — Appendix 01 (PDF) [file pnas.2415934121.sapp.pdf]

Supporting Information for

Chemical mapping of the surface interactome of PIEZO1 identifies CADM1 as a modulator of channel inactivation

Anna K. Koster[a,b], Oleg Yarishkin[a], Adrienne E. Dubin[a], Jennifer M. Kefauver[a], Ryan A. Pak[a], Benjamin F. Cravatt*[b], Ardem Patapoutian*[a]

Corresponding author(s): Ardem Patapoutian, Benjamin F. Cravatt
Email: ardem@scripps.edu , cravatt@scripps.edu

This PDF file includes:

Supporting Materials and Methods
Figures S1 to S5
Tables S1 to S6
Appendix I: Electrophysiology screen data, as summarized in Table S1
Appendix II: cDNA library information
Appendix III: Calcium imaging screen data
Supplementary File 2: Spreadsheet Legend
SI References

Other supporting materials for this manuscript include the following:

Supplementary File 2 (proteomics summary data*)

*Raw data for TMT mass spectrometry experiments have been deposited in ProteomeXchange Consortium(1) via the PRIDE partner repository(2): Accession No. PXD054618.

Supporting Information: Materials and Methods

Molecular biology: *Subcloning of cDNA library.* For expression studies, cDNA sequences contained in Gateway pDONR vector backbone were subcloned as fusion constructs into mammalian expression vectors with a C-terminal mCherry tag (Addgene, Plasmid#31907) via the LR recombination reaction. Briefly, the destination vector was amplified by transforming into One Shot® *ccd*B Survival 2 T1^R Chemically Competent *E. coli* (Life Technologies, Cat#A10460) using chloramphenicol (25 µg/mL) and kanamycin (50 µg/mL) in Luria broth (LB) or agar for antibiotic selection. Plasmid DNA was purified from the bacterial pellet using the QIAprep spin miniprep kit (Qiagen, Cat#27106), according to manufacturer instructions. For the LR reaction, donor vector (1 µL, 100 ng) was combined with destination vector (1 µL, 150 ng) and 1 µL Gateway™ LR Clonase™ II enzyme mix (Life Technologies, Cat#11791020) in 7 µL TE buffer, pH 8.0 (10 mM Tris-HCl, 1 mM EDTA). Following overnight incubation at room temperature, 2 µL of the recombination reaction were transformed into 20 µL NEB 10-beta competent *E. coli* (New England Biolabs, Cat#C3019H) and selected with kanamycin (50 µg/mL).

Subcloning of untagged cDNAs into IRES-tdTomato or IRES-mCherry expression vectors was accomplished by polymerase chain reaction (PCR)-based cloning. Briefly, forward and reverse PCR amplification primers were designed for each gene containing a 6-basepair leader sequence to assist with restriction digest (5'-3' Forward leader: TAAGCA, Reverse leader: TGCTTA) the appropriate restriction site, and a hybridization sequence complementary to the sequence to be amplified. The length of the hybridization sequence was optimized to match the annealing temperatures of the forward and reverse primers as closely as possible. If not already included in the expression vector, a Kozak sequence (GCCACCATGG) for strong initiation of translation was incorporated into the forward primer, and a stop codon (TAA or TAG) was incorporated into the reverse primer. Genes were amplified via PCR with Phusion High-Fidelity DNA polymerase (Fisher Scientific, Cat#F530L) in HF Phusion buffer, according to suggested manufacturer thermocycling conditions. Annealing temperatures were determined using the NEB T_m Calculator tool (https://tmcalculator.neb.com) and a 30 seconds-per-kilobase extension time. PCR products were purified using the QIAQuick PCR purification kit (Qiagen, Cat#28104). The purified PCR product and recipient plasmid backbone were digested with the appropriate pair of restriction enzymes (Thermo Scientific, FastDigest) and loaded onto a 1% agarose gel containing SYBR™ safe DNA gel stain (Life Technologies, Cat#S33102) to verify correct amplicon and recipient plasmid backbone lengths (using NEB 1 kb Plus DNA ladder, Cat#N3200S). The digested PCR product and recipient plasmid backbone were excised from the agarose gel and purified using the ZymoClean Gel DNA recovery kit (Zymo Research, Cat#D4002). The purified DNA fragments were ligated in a 3:1–5:1 ratio of PCR insert to backbone recipient plasmid using T4 DNA ligase (New England Biolabs, Cat#M0202L), according to the suggested manufacturer protocol. Following a 10-minute incubation at room temperature, 2 µL of the recombination reaction were transformed into 20 µL of NEB 10-beta competent *E. coli* (New England Biolabs, Cat#C3019H) and selected with the appropriate antibiotic selection marker (kanamycin or carbenicillin).

Additional PIEZO construct information. The PIEZO1-BBS (blade) construct contains an I → M single nucleotide polymorphism (SNP) in the *Piezo1* coding sequence at position 105 (in the canonical sequence without the BBS tag), and *Piezo1*-BBS (cap) contains an L → P SNP at position 574 relative to the canonical mPIEZO1 sequence. These constructs traffic to the plasma membrane normally and are functionally similar to canonical mPIEZO1, as previously characterized by the Grandl lab(3) (and confirmed by our lab). Both *Piezo1* constructs reside in the pcDNA3.1(–) backbone and are ampicillin resistant, and K_v1.2-BBS-S1-S2-IRES-GFP is resistant to kanamycin.

Cell surface proximity labeling of PIEZO1. Prior to starting the experiment, a radical quenching solution and diluted solution of hydrogen peroxide were freshly prepared and kept on ice. The quenching solution was composed of 5 mM Trolox ((±)-6-hydroxy-2,5,7,8-tetramethyl-chromane-2-carboxylic acid, CAS# 53188-07-1, Sigma-Aldrich, Lot# BCBW5446, from a 500 mM stock solution in DMSO), 10 mM L-ascorbic acid sodium salt (ACROS, Fisher Scientific, Catalog#AC352680050, CAS#134-03-2, from a 1M stock in water), and 10 mM sodium azide (ReagentPlus CAS#26628-22-8, #S2002-5g, Sigma-Aldrich from a 1 M stock in water) in DPBS (Gibco, Cat#14190-136).

Hydrogen peroxide (30% w/w in water, Sigma-Aldrich Cat#H1009-100mL, CAS#7722-84-1) was diluted to 0.3% in deionized water and kept on ice until addition.

To append HRP to PIEZO1 channels on the cell surface, each 15-cm dish of cells was first incubated with 8 µL (500 µg/mL) of α-bungarotoxin biotin-XX conjugate stock (Invitrogen, Cat#B1196) per 20 mL media at 37 °C for 30 min (final concentration of 0.2 µg/mL, 1:5000, ~25 nM). At the end of the incubation, cells were washed with 2 × 20-mL DPBS (Gibco, Cat#14190-136) and incubated with 15 mL of complete DMEM media containing 15 µL (1 mg/mL stock solution in DPBS) of streptavidin horseradish peroxidase (strp-HRP, Invitrogen Life Technologies Cat#S911, 1:1000 dilution) for 20–30 minutes at room temperature. The strp-HRP solution was gently rocked over the cells, manually, two times during the incubation. Cells were washed with 3 × 20 mL DPBS to remove any unbound strp-HRP. For proximity labeling, a 250 mM stock of biotin phenol (BP, CAS#41994-02-9, APExBIO Cat#A8011, Boston, MA) was prepared in cell culture grade DMSO and added to 20 mL of complete DMEM (final concentration 250 µM). The BP-DMEM (20 mL) was added to the plate followed by immediate addition of 200 µL of 0.3% hydrogen peroxide (final concentration during labeling is 0.003%). The plate was gently rocked back and forth, manually, to evenly disperse H_2O_2 and then incubated for exactly 30 seconds. After 30 s, DMEM-BP was gently removed and replaced with 10 mL of ice-cold quencher solution for a total labeling time of 45–60 s prior to radical quenching. After 20–60 s, the solution was removed and replaced with an additional 15 mL of fresh, ice-cold quenching solution. The plate was placed on ice until cell collection. For labeling in the presence of the PIEZO1 agonist Yoda1, 100 µL of 2 mM Yoda1 in DMSO (TOCRIS, Cat.#558610, CAS#448947-81-7) or 100 µL of DMSO vehicle control was added to 20 mL of the BP-DMEM solution and vigorously vortexed (10 µM final concentration of Yoda1 during labeling). Yoda1 is poorly soluble in aqueous solutions, so it is critical to perform this step just before addition to the plate to ensure that Yoda1 does not precipitate prior to addition. To keep the timing consistent, plates were treated with α-bungarotoxin biotin and strp-HRP in pairs, and biotinylation was performed one plate at a time. To maintain integrity of cell surface proteins, cells were mechanically dissociated with a cell scraper and washed with additional cold DPBS for collection in a Falcon tube. Cells were pelleted at 80×g for 3 minutes at 4 °C. Each cell pellet was gently resuspended in 5-mL of ice cold DPBS, centrifuged again, and resuspended a final time in 13.5 mL of cold DPBS (0.5 mL of this was retained for Western blot to verify biotinylation prior to MS). The cells were pelleted at 80 × g for 3 minutes, the supernatant was removed, and the cell pellet was flash frozen in liquid nitrogen prior to storage at –80 °C.

Western blot for characterization of PIEZO1-BBS (cap) stable cell line. Cells were lysed with 1% NP-40 lysis buffer (25 mM Tris-HCl pH 7.4; 150 mM NaCl; 10% glycerol; 1% Nonidet P-40 in water) containing one protease inhibitor cocktail tablet (Roche, cOmplete ULTRA tablets, Mini, EDTA-free, Cat#5892791001) per 10 mL of lysis buffer. Cell pellets were kept on ice and resuspended in lysis buffer by vortexing, followed by probe sonication for 10 pulses at 4 °C (40%, output 4). Samples were centrifuged at 16,300×g for 10 minutes at 4 °C, and the supernatant was retained for protein quantification. Protein was quantified using a DC absorbance assay (BioRAD Cat#5000112) and serial dilutions of a 2 mg/mL bovine serum albumin protein standard, according to the manufacturer instructions. Protein concentrations were normalized and loaded (18–45 µg total protein per lane) onto a 4–20% Novex Tris-Glycine gradient gel (Invitrogen, Cat#XP04202BOX) with denaturing 1×Laemmli loading buffer (250 mM Tris-HCl pH 6.8; 8% sodium dodecyl sulfate; 10% glycerol; 20% β-mercaptoethanol; ~5 mM bromphenol blue). Samples were not heated prior to gel loading to avoid membrane protein aggregation. The gel was run for 1.5 h at 150 V. Proteins were transferred from the gel to an activated 0.2 µm PVDF membrane (BioRad Cat#1620177) with Towbin transfer buffer (25 mM Tris, 192 mM glycine, 20% MeOH v/v, pH 8.3) for 1.5 h at 400 mA constant current. Following transfer, the PVDF membrane was incubated in a TBS-T buffer (20 mM Tris-HCl pH 7.6, 150 mM NaCl with 0.1% Tween 20) containing 5% milk (Research Products International, Cat#M17220-1000) for 1 h at room temperature. Following block, the membrane was incubated with strp-HRP antibody (Invitrogen Life Technologies Cat#S911, 1:5000) overnight (16 h) at 4 °C. Before developing, the membrane was washed 3× with TBS-T buffer for 5 minutes on a rocker. Biotinylation was visualized with SuperSignal West pico PLUS (Thermo Scientific, Cat#34579), using a BioRad ChemiDoc XRS.

Mass spectrometry. *Preliminary method development.* As a proof of concept, we transiently expressed two PIEZO1 constructs with the BBS tag inserted into either the extracellular cap or distal blade. Both of these constructs are expressed normally at the plasma membrane and behave like wild-type PIEZO1(3). As controls, we additionally expressed a BBS-tagged voltage-gated potassium channel (K_v1.2-BBS)(3) and a generic plasma membrane targeted HRP (HRP-TM, Addgene plasmid #44441)(4) that should be non-specifically expressed over the entire cell surface. Because the radius of biotinylation is akin to a contour map in which the extent of biotinylation drops off as a function of distance from HRP, we hypothesized that ratiometric analysis of proteins enriched more strongly by PIEZO over other membrane protein controls are more likely to be specific interaction partners. While roughly the same set of proteins was captured across datasets, we found that quantitative prioritization of protein interaction candidates proved challenging based on our initial assumptions. First, we found that ratiometric evaluation and ranking of proteins enriched by PIEZO1-BBS compared to K_v1.2-BBS and HRP-TM was inconsistent among replicates, with HRP-TM completely overwhelming the PIEZO1-BBS signal and K_v1.2-BBS producing a lower signal across the proteome, presumably due to lower expression at the plasma membrane. In addition to the challenges of normalizing the plasma membrane expression of each construct, PIEZO1 and K_v1.2 are composed of multiple subunits (trimer and tetramer, respectively). Even if expression levels had been equivalent, other factors, such as the number of HRP molecules appended per ion channel, as well as the exposure of the BBS-tagged site above the plasma membrane, created difficulties in quantitative interpretation. For example, we found that despite having similar expression levels at the plasma membrane(3), the PIEZO1-BBS (cap) construct consistently produced higher signal for the same set of proteins than the PIEZO1-BBS (blade) construct, presumably because the cap domain protrudes higher into extracellular space and is more accessible to labeling(3). In light of these unanticipated challenges, we ultimately chose to generate a stable cell line containing the PIEZO1-BBS (cap) construct and use previous overexpression TMT datasets that we generated during method development as one of our data filters. That is, only proteins captured in the stable cell line dataset (closer to endogenous conditions) and all three complementary overexpression TMT datasets were considered to be potential PIEZO1 interaction partners (see 'Data analysis' in the mass spectrometry section of 'Materials and Methods'). As such, this technique is most amenable to comparisons of the same protein interactome across conditions, such as the presence and absence of a mechanical stimulus. This allows for quantitative analysis of stimulus-specific interactome changes while circumventing the need for other plasma membrane controls that may be more difficult to interpret.

Mass spectrometry (detailed protocol). *Protein precipitation:* Cell pellets collected as described in "proximity labeling" were lysed by probe sonication (10 pulses at 4 °C, 40%, output 4) in 500 µL of ice cold DPBS (Gibco, Cat#14190-136) containing one protease inhibitor cocktail tablet (Roche, cOmplete ULTRA tablets, Mini, EDTA-free, Cat#5892791001) per 10 mL. Protein concentrations were quantified using a DC absorbance assay (BioRAD Cat#5000112) and diluted to a final concentration of 3 mg/mL in 500 µL DPBS (1.5 mg of total protein from each 15-cm plate of HEK293T cells). Protein was precipitated by sequential addition and mixing of ice-cold methanol (2 mL), chloroform (0.5 mL), and DPBS (1 mL) followed by vortexing for 30 seconds and centrifugation at 5,000 rpm for 10 minutes to create a protein disk. The top and bottom layers around the disk were gently removed, and the pellet was washed with 2×10 mL of ice-cold methanol with 5,000 rpm centrifugation between washes. After the final wash, the supernatant was carefully removed from the pellet by pipette with a gel-loading tip, and the pellet was allowed to air dry for 15 minutes before further processing. Sample pellets may be stably stored in a –80 °C freezer prior to subsequent denaturing, reducing, and alkylation steps.

Denaturation, reduction, and alkylation: Each protein sample pellet was denatured by adding 500 µL of freshly prepared 6 M urea in DPBS, followed by 10 µL of 10% w/v sodium dodecyl sulfate (SDS) in DPBS. To reduce the protein sample, a solution containing equal volumes of freshly prepared 200 mM tris(2-carboxyethyl)phosphine hydrochloride (TCEP) in DPBS and 600 mM potassium carbonate in DPBS was prepared, and 50 µL of this mixture was added to the denatured sample. Each sample was then probe-sonicated 15× (40%, output 4) and placed in a 37 °C shaker for 30 minutes. Following this incubation, each sample was alkylated by adding 70 µL of 400 mM iodoacetamide in DPBS, briefly mixing, and incubating in the dark at room

temperature for 30 minutes. After alkylation, an additional 130 μL of 10% SDS in DPBS was added to each sample, then diluted with 5.5 mL of DPBS for a final volume of 6.26 mL per sample (~0.5 M urea, 0.2% w/v SDS).

Streptavidin pull-down and tryptic digest: Streptavidin agarose beads (100 μL per sample, Thermo Scientific, Cat#20353) were washed 3×500 μL with DPBS and pelleted at 2000 × g in between washes. After the final wash, the beads were re-suspended in 100 μL of DPBS and added to each 6.26-mL protein sample in a 14-mL centrifuge tube. Samples were rotated for 1 h at room temperature on a benchtop end-over-end rotator to pull down biotinylated proteins, followed by pelleting the beads at 2500 × g for 2 minutes. Supernatant was carefully removed, and the bead slurry was washed sequentially with: 1) 1 × 5 mL of 0. 2% w/v SDS in DPBS, 2) 3 × 5 mL of DPBS, 3) 1 × 5 mL of HPLC-grade water, and 4) 2 × 5 mL of 200 mM EPPS buffer (pH 8.0 with NaOH). Samples were centrifuged at 2500 × g for 2 minutes in between washes. Beads were resuspended in 500 μL of EPPS (pH 8.0) and transferred to a 1.5-mL safe-lock, LoBind Eppendorf™ tube (Fisher Scientific, Cat#13-698-794). Residual beads were transferred with an additional 500 μL of EPPS (total volume of 1 mL) and pelleted at 2500 × g for 2 minutes. Supernatant was carefully removed from the beads with a fine gel-loading pipette tip. A 20-μg vial of sequencing-grade trypsin (Promega, Cat# V5111) was dissolved in 2.05 mL of 2 M urea in 200 mM EPPS buffer (pH 8.0). Calcium chloride (100 mM in 200 mM EPPS buffer, pH 8.0) was diluted 1:100 into the trypsin-containing solution (20.5 μL added, 1 mM final concentration) and gently mixed by pipetting. On-bead tryptic digest was performed by adding 200 μL of this trypsin solution to each protein sample and incubating for ~14 h on a 37 °C shaker.

TMT labeling and fractionation of peptides: Beads were removed from tryptic peptides by filtering the digested sample through a micro-Bio-Spin chromatography column (Bio-Rad, Cat#7326204) into a clean 2-mL centrifuge tube. HPLC-grade acetonitrile (60 μL) was added to the eluted peptides (260 μL total volume), followed by 6 μL/channel of 10-plex isobaric TMT tag (Thermo Scientific, Cat#90110) dissolved in dry acetonitrile (20 μg/μL). The reaction was allowed to proceed for 1 h at room temperature with an occasional vortex, then quenched with 6 μL of 5 wt. % hydroxylamine in HPLC-grade water for 15 minutes at room temperature. Formic acid (4 μL) was added to each sample. All ten TMT-labeled samples were then combined into a single 14-mL centrifuge tube, then divided evenly into four protein LoBind microcentrifuge tubes to dry in a SpeedVac concentrator overnight. The resulting peptide/urea pellets were re-dissolved in Buffer A (95% H_2O, 5% acetonitrile, 0.1% formic acid) and sonicated in a water-bath sonicator for 10 minutes to ensure complete solubilization. Samples were recombined into a single tube followed by addition of 20–60 μL of HPLC-grade formic acid to reach a pH of 1–3. The peptide sample was manually fractionated offline into 9 fractions on a Pierce™ high pH reversed-phase peptide fractionation column (Thermo Scientific Cat#84868), using a gradient elution of acetonitrile (5, 7.5, 12.5, 15, 20, 25, 30, 40, 50, 80%) in 10 mM aqueous ammonium bicarbonate. Every third fraction (e.g., 1, 4, and 7) of eluted peptides was recombined into a 1.5-mL LoBind Eppendorf tube for a total of 3 fractions and dried overnight in a SpeedVac. Samples were resuspended in buffer A by bath sonication and analyzed on an Orbitrap Fusion mass spectrometer (Thermo Scientific). Data acquisition was by an MS3-based TMT method as previously described(5).

Data analysis: The Integrated Proteomics Pipeline (IP2) was used for initial analysis of raw data files. MS2 and MS3 spectra were analyzed using the ProLucid search algorithm against a non-redundant variant of the human Uniprot database (release-2012_11). Allowed residue modifications in the search algorithm were as follows: static modification of cysteine residues for carboxyamidomethylation (+57.02146 Da), static modification of lysine residues and the N-terminus corresponding to a TMT tag (+229.162932 Da), and differential modification of methionine residues accounting for oxidation (+15.9949 Da). Proteins were required to have at least two peptides for identification with a false discovery rate set at 0.01 and a spectral count (SC) cutoff of 5. Biological replicates of comparison groups were run in duplicate or triplicate for internal TMT comparison and were also repeated in separate TMT runs to ensure reproducibility of results. Four TMT datasets were included in analysis: three with transient overexpression of PIEZO1-BBS (cap) in HEK293T cells and one in the PIEZO1-BBS (cap) stable HEK293F cell line. Proteins not contained in all four TMT datasets were removed from the final list of proteins in the PIEZO1 interactome. Proteins were further ranked by comparing each dataset to the Contaminant Repository for Affinity Purification (CRAPome 2.0), giving priority to proteins found in ≤ 25% (179/716) of datasets and thereby

removing contaminants and other highly abundant proteins commonly found in proximity-dependent biotinylation datasets (also see Supplementary File 2). Gene ontology (GO) analysis and manual annotation of the removed proteins based on the Human Protein Atlas(6) showed that the majority of these are annotated as intracellular or cytosolic, likely captured from dead cell debris (Supplementary File 2). To evaluate the extent of background labeling, all datasets were cross-referenced to TMT channels in which all labeling and biotinylation reagents were added to cells expressing mPIEZO1-GFP without a BBS epitope tag. Any proteins equally enriched by PIEZO1 lacking a BBS tag were removed from consideration and included only a small set of biotin-associated proteins (PC, ACACA, ACACB, MCCC1, PCCA) and keratin (KRT), which had previously been eliminated from the datasets by our CRAPome filter.

Calcium imaging additional information. Calcium Ringer's solution used for washing, loading, and imaging cells contained 127 mM NaCl, 3 mM KCl, 10 mM HEPES, 2.5 mM $CaCl_2$, 1 mM $MgCl_2$, and 10 mM D-(+)-glucose (pH 7.3 with NaOH, 300-310 mOsm/kg adjusted with D-mannitol). Cells were loaded at room temperature in the dark for 35–40 minutes with ~1 μM Fura2-AM (Life Technologies, Cat#F1201, from a 1 mg/mL stock in DMSO) and 0.02% w/v Pluronic F-127 (Invitrogen, Cat#P6867, from a 20% w/v stock in DMSO freshly prepared and used within one week) in calcium Ringer's. To remove excess dye, cells were washed 3× with calcium Ringer's solution and allowed to sit in the dark for 20 minutes prior to imaging. Yoda1 (TOCRIS, Cat.#558610, CAS#448947-81-7) was prepared as a 2 mM solution in DMSO, diluted 1:200 in calcium Ringer's (10 μM, 2×), and vigorously vortexed 1–2 minutes before adding to each well (final concentration in the well, 5 μM; 170 μL of a 2× solution added to 170 μL of calcium Ringer's in the well). Use of higher stock concentration of Yoda1 and/or preparing the solution ahead of the experiment may result in inconsistent results due to the propensity of Yoda1 to precipitate in aqueous solution over a short time frame. Following 30 seconds of baseline recording, Yoda1 solution was pipetted in a single addition near the bottom corner of the imaging well (without pipetting up and down) to mix with the solution in the chamber as rapidly as possible (peak Yoda1 response occurs within 30 s of addition). Adding a DMSO vehicle control in this manner produced no response, indicating that shear stress was not responsible for the observed effects. At the end of each recording, a 170 μL of a 12 μM (3×) solution of ionomycin calcium salt in calcium Ringer's (Sigma-Aldrich, Cat#I0634 from a 5 mM stock in DMSO) was added to the well (4 μM final concentration in the well). Cells that did not respond to ionomycin were excluded from analysis. Data were acquired every 3 s using MetaFluor (v.7.8.2.0) as a ratio of 340/380nm. At least two wells per gene were tested on the same day. Candidate genes showing consistent effects for both replicates were repeated on at least one other day to confirm the observed effect and then were validated by electrophysiology under the same conditions.

Electrophysiology additional information. *Equipment and software.* Electrodes (Sutter, Cat#BF150-86-10) were pulled with a Sutter Instruments flaming/brown micropipette puller (Model P-97) and fire polished with a Micro forge (Model MF-830, Narishige, Japan) to a range of resistances from 2–6 MΩ. For poking experiments, the polished glass poke probe was advanced with a ramp speed of 1 μm/ms and held at the indicated position for 125 ms, driven by a piezoelectric controller and actuator (Physik Instrumente, E-625.SR Compact LVPZT controller/amplifier and P-601.1SL PiezoMove® nanopositioning actuator) attached to the micromanipulator with a custom dye-anodized aluminum adapter at an 80° angle to the cell being recorded(7). All recordings were performed using an Axopatch 200B or a Multiclamp 700A amplifier and acquired using Axon™ pClamp10 (Molecular Devices). Data were analyzed using Clampfit 10.6, OriginPro 10 (OriginLab), and GraphPad Prism v.10.1.2 (DotMatics).

Whole-cell recording solutions. Extracellular recording solution contained: 135 mM NaCl, 3 mM KCl, 1 mM $MgCl_2$, 2.5 mM $CaCl_2$, 10 mM D-glucose, 10 mM HEPES (pH 7.3 with NaOH; 300-310 mOsm/kg adjusted with D-mannitol to be ~10 mOsm higher than intracellular solution, if necessary). Intracellular recording solution contained: 133 mM CsCl, 5 mM EGTA, 1 mM $MgCl_2$, 1 mM $CaCl_2$, 10 mM HEPES, 4 mM Mg·ATP*, 0.4 mM Na_2GTP* (pH 7.3 with CsOH; *10 μL of frozen aqueous ATP and GTP stocks, 400 and 40 mM, respectively, were added to 980 μL aliquots of intracellular solution directly before the experiment, and the pH readjusted back up to 7.3 with ~1.5 μL of 2N CsOH; 285–300 mOsm/kg).

Cell-attached recording solutions and additional details. Extracellular bath solution contained: 140 mM KCl, 1 mM $MgCl_2$, 10 mM D-glucose, 10 mM HEPES (pH 7.3 with KOH). Pipette solution contained: 130 mM NaCl, 5 mM KCl, 1 mM $MgCl_2$, 1 mM $CaCl_2$, 10 mM TEA-Cl, 10 mM HEPES (pH 7.3 with NaOH). Cells were maintained in high-K^+ bath solution for no longer than 30 minutes. The holding potential was set as –80 mV during recording. Cells were mechanically stimulated as previously described(7) using a high-speed pressure clamp (HSPC-1) device (ALA Scientific Instruments) controlled by Clampex 10.1. A positive pressure pre-pulse of +5 mmHg (5.75 s) was applied followed by a 25-ms step to 0 mmHg before each successive negative pressure step in increments of –5 mmHg. This pre-pulse protocol was shown to minimize membrane curvature and produce maximal response to negative pressure(8).

CADM1 knockdown and overexpression experiments in Neuro-2a cells. *qRT-PCR:* For quantification of siRNA knockdown, RNA was extracted using an RNeasy Micro kit (Qiagen, Cat#74004), according to the manufacturer instructions. The RNeasy kit on-column DNaseI digest step was omitted, and digestion with DNaseI was instead performed immediately prior to reverse transcription, using the Maxima H-minus First Strand cDNA Synthesis Kit with dsDNase (Life Technologies, Cat#K1681). Integrity and purity of input RNA was evaluated by 260/280 nm absorbance ratios (~2). Synthesis of cDNA was performed immediately after RNA isolation, using 1 µg of total RNA and Oligo(dT)$_{18}$ primers provided in the kit. The cDNA products were diluted 1:25 in nuclease-free water and mixed 1:5 with SyGreen Blue Mix (Genesee Scientific, Cat#16-507) containing the following primer pairs (500 nM):

Gene (amplicon length)	**Source**	**Cat#**	**Accession#**	**Primer pairs (5' to 3')**
Actb (138 bp)	Origene	MP200232	NM_007393	FWD: CATTGCTGACAGGATGCAGAAGG<br>REV: TGCTGGAAGGTGGACAGTGAGG
Cadm1 (113 bp)	Origene	MP202240	NM_207675	FWD: ACTTCTGCCAGCTCTACACGGA<br>REV: CCCTTCAACTGCCGTGTCTTTC
Cadm1 (99 bp)	Harvard Primer Bank(9–11)	N/A	NM_207675	FWD: CCACAGGTGATGGACAGAATC<br>REV: CTGAGTCGTCACTCTTATTGACC

The cycling parameters used for qRT-PCR on a BioRad CFX384 real-time PCR system were 95 °C for 3 minutes and then 40 cycles of 95 °C for 10s and 60 °C for 30s, followed by a melt curve. Primer specificity was evidenced by a single peak in the derivative of the melt curve and by presence of a single band of the expected amplicon length on a 2% agarose gel (Fig. S4, B–C). Differences between the non-targeting scramble control and knockdown conditions were quantified by a change in the C_t value relative to *Actb*, where $\Delta C_t = C_t(Cadm1) – C_t(Actb)$ for each condition (Fig. S4, D). Fold changes were calculated by the $\Delta\Delta C_t$ method where, $\Delta\Delta C_t = \Delta C_t$ (knockdown condition)–ΔC_t (non-targeting control condition) and fold change = $2^{-(\Delta\Delta Ct)}$ with three biological replicates per condition and three technical replicates per biological replicate.

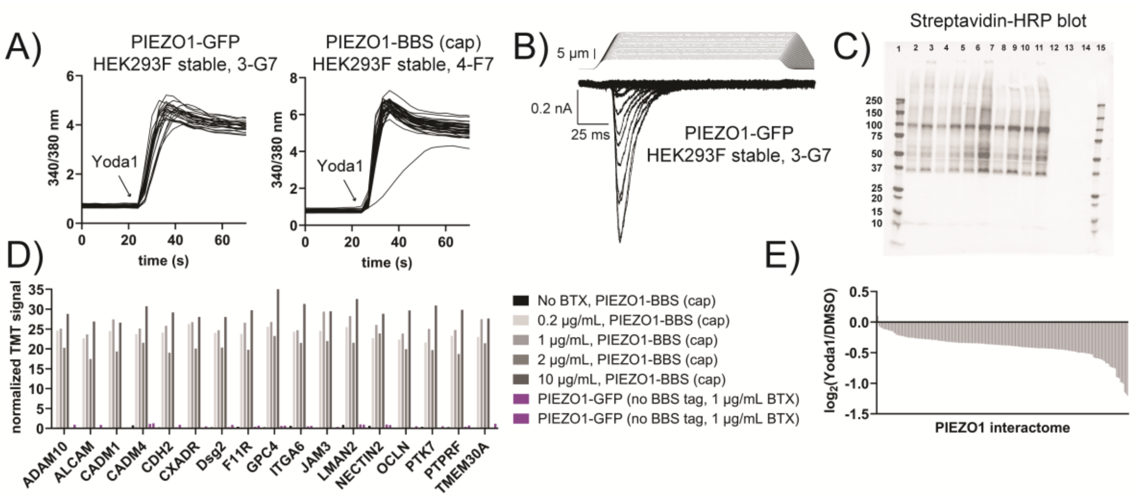

Fig. S1. Functional characterization of PIEZO1 stable cell lines used for proteomics and calcium imaging. A) Representative ratiometric calcium imaging raw data for mPIEZO1-GFP HEK293F stable cells (clone 3-G7) used in the calcium imaging screen and for mPIEZO1-BBS (cap)-GFP HEK293F stable cells (clone 4-F7) used for proximity labeling. Both cell lines produce a fairly uniform increase in intracellular Ca^{2+} in response to 5 μM of the Yoda1 channel agonist. B) Representative family of whole-cell currents elicited in response to poking in increasing 0.5 μm increments to the stable cells (clone 3-G7) used in the calcium imaging screen reveals wild-type-like PIEZO1 kinetics (inactivation tau mean ± SEM: 9.8 ± 0.7 ms, n = 24). C) Representative streptavidin-HRP blot showing the biotinylated proteome of various PIEZO1-BBS (cap) clones derived from the HEK293F stable cell line. In the presence of α-BTX-biotin, H_2O_2, and biotin-phenol, a range of biotinylated proteins are visible. Clone 4-F7 used for proteomics experiments is shown in lanes 10–11 with 18 or 45 μg of total proteome loaded, respectively. In contrast, no biotinylation is visibly detected for a representative clone derived from the PIEZO1-GFP HEK293F stable cell line in the presence of the same labeling reagents (lanes 12–13). This is consistent with the proteomics data indicating that there is little to no background labeling if the BBS tag is not present (see panel D). D) Representative data from a single TMT optimization experiment in HEK293T cells, showing the relative TMT signal (normalized to 100%) over 7 TMT channels reveals that 0.2 μg/mL (~25 nM) α-BTX performs equivalently to higher concentrations (up to 10 μg/mL, 1.25 μM). Increasing concentrations are shown left to right (gray bars) for a representative set of candidate proteins that were later tested by electrophysiology. Notably, little to no MS signal is detected when cells expressing PIEZO1-BBS (cap) are not exposed to α-BTX prior to biotinylation with H_2O_2 and biotin-phenol (black bars), or when cells expressing PIEZO1-GFP without the BBS tag are exposed to all labeling reagents (fuchsia bars). E) Plot showing the 185 high-confidence PIEZO1 interactome proteins (listed in Figure 1B) in the presence of Yoda1 (10 μM, applied over ~1-2 min.) verses a DMSO vehicle control, averaged over three internal biological replicates in a TMT 6-plex experiment. Experiments were performed with the PIEZO1-BBS (cap) HEK293F stable cell line (clone 4-F7). We observed a small but noticeable and consistent global decrease in the extent of biotinylation in the presence of Yoda1 (see Supporting Information, File 2).

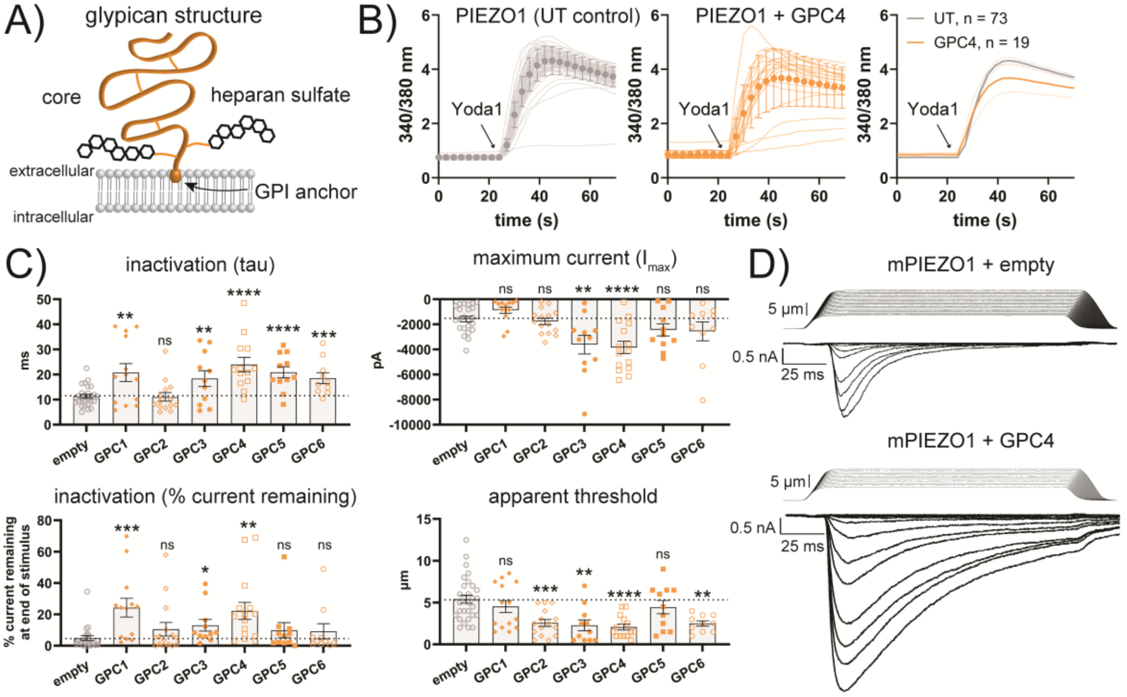

Fig. S2. Characterizing the functional effects of glypicans on PIEZO1. A) Cartoon of glypican (GPC) structure. B) Representative calcium imaging raw data in 3-G7 cells showing the Yoda1 response in cells visibly transfected with GPC4 (orange) vs. untransfected (UT) cells (gray) in the same field of view (n = 19 and n = 73 cells, respectively). Markers show the average ± SD; Right: average of raw traces shown at the left with dotted lines indicating the 90% confidence interval. C) Whole-cell poke electrophysiology parameters in HEK293T PIEZO1 KO cells co-transfected with mouse *Piezo1*-IRES-GFP and each untagged GPC family member in an IRES-tdTomato vector (see Table S2 for average values and statistical analysis). D) Representative mPIEZO1 whole-cell mechano-currents for PIEZO1 co-expressed with an empty vector control or with GPC4. When overexpressed with GPC4, PIEZO1 expressing cells appeared sensitized to mechanical indentation (i.e., they exhibited a lower apparent threshold), had larger apparent maximal currents, and inactivated more slowly than wild-type mPIEZO1. In the mPIEZO1 + GPC4 example shown, PIEZO1 began responding at a shallow indentation depth of 1 µm and reached its maximum current before the top wild-type mPIEZO1 cell started responding (5.5 µm).

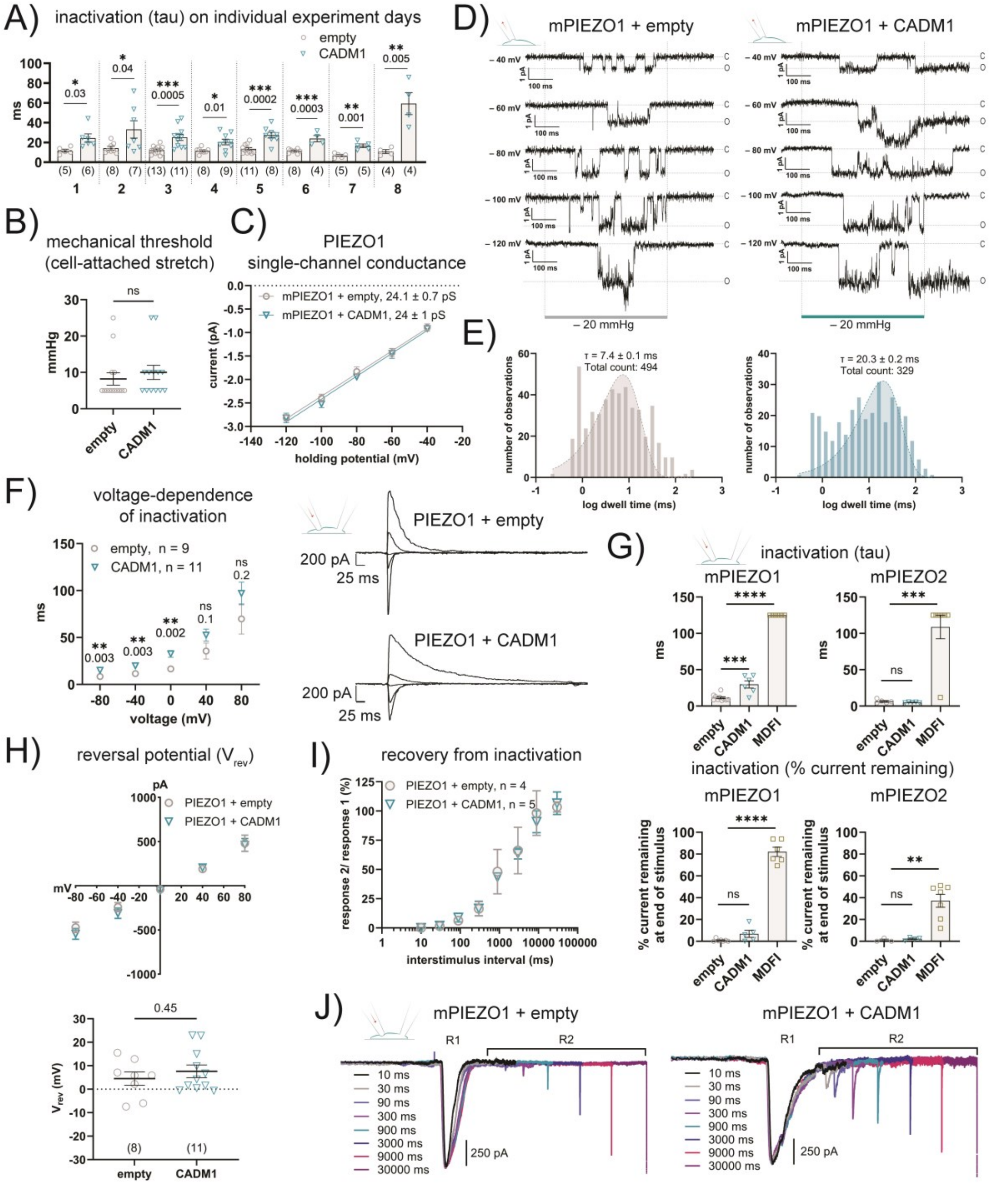

Fig. S3. Additional characterization of CADM1 on PIEZO1 and PIEZO2. All values in this figure are expressed as the mean ± SEM. A) Inactivation time constant (tau, ms) of whole-cell poke-induced mPIEZO1 currents in cells transfected with mouse *Piezo1* and either an empty vector control or co-expressed with *CADM1* on individual recording days (1–8) in HEK293T PIEZO1 knockout cells, shown as pooled data in Figure 3A. The number of cells for each condition (n) are indicated below each bar, and the *p*-values, as calculated by an unpaired two-tailed *t*-test, are shown on the graph. B) Quantification of the PIEZO1 mechanical threshold in cell-attached stretch assay in HEK293T PIEZO1 knockout cells overexpressing an empty vector or CADM1 (empty: 8 ± 2 mmHg, n = 14; CADM1: 10 ± 2 mmHg, n = 13; *p* = 0.5). These results are consistent with little to no change in the mechanical threshold of PIEZO1 with CADM1 in whole-cell poke experiments

(Table S1, Appendix I). C) Summary data of PIEZO1 unitary conductance as measured by recordings in panel D, showing no change in single-channel conductance in the presence of CADM1 (empty: 24.1 ± 0.7 pS, n = 5 vs. CADM1: 24 ± 1 pS, n = 5). D) Representative cell-attached stretch single-channel recordings for PIEZO1 in the presence of overexpressed empty vector control or CADM1. Data are shown for the same cell with voltage steps from –40 to –120 mV in 20 mV increments and an applied negative pressure stimulus of –20 mm Hg for 500 ms (stimulus duration shown by gray/turquoise bar; C = closed, O = open). E) Histograms of open-channel dwell times from single-channel recordings of PIEZO1 in the absence (gray) or presence (turquoise) of CADM1, showing a nearly 3-fold increase in the time PIEZO1 spends open when CADM1 is present (empty: 7.4 ± 0.1 ms, event count = 494; CADM1: 20.3 ± 0.2 ms, event count = 329). F) PIEZO1 voltage-dependence of inactivation as measured by whole-cell poke electrophysiology at –80, –40, 0, +40, and +80 mV in the presence of an empty vector control or CADM1 overexpressed in HEK293T PIEZO1 KO cells. Representative traces are shown at the right with quantification shown left for the indicated number of cells. Inactivation is slower and more variable at positive voltages and is not statistically significant for this number of cells, but CADM1 appears to slow inactivation at all voltages. G) Comparison of the effects of CADM1 and MDFI on PIEZO1 and PIEZO2 from whole-cell poke recordings performed in parallel on the same day. Co-expression with MDFI showed an inactivating phenotype for both mPIEZO1 and mPIEZO2. For MDFI, tau is plotted at 125 ms, the end of the stimulus, but the current persisted beyond the end of the mechanical indentation (n = 6, $p < 0.0001$ and n = 7, $p = 0.0004$ for PIEZO1 and PIEZO2, respectively), as previously reported(12). This stands in contrast to CADM1, which produced a noticeable effect on inactivation with PIEZO1 (empty tau: 11 ± 1 ms, n = 10; CADM1 tau: 30 ± 5 ms, n = 6; $p = 0.0007$) but not PIEZO2 (empty tau: 6 ± 1 ms, n = 5; CADM1 tau: 5.1 ± 0.3 ms, n = 5; $p = 0.3$). In the presence of MDFI, PIEZO2 inactivated to a greater extent than PIEZO1 over this 125-ms interval but was still significantly inactivating in both conditions (as measured by the percent current remaining at the end of the stimulus). CADM1 showed a trend towards more current remaining at the end of the stimulus, in line with the results in Fig. 3A (current remaining: PIEZO1 + empty, 0.9 ± 0.5%, n = 7; PIEZO1 + CADM1, 7 ± 3%, n = 5; PIEZO1 + MDFI, 82 ± 4%, n = 6; empty vs. CADM1, $p = 0.05$; empty vs. MDFI, $p < 0.0001$; PIEZO2 + empty, 1 ± 1%, n = 3; PIEZO2 + CADM1, 2.2 ± 0.7%, n = 3; PIEZO2 + MDFI, 37 ± 6%, n = 7; empty vs. CADM1, p = 0.3; empty vs. MDFI, $p = 0.005$). H) Evaluation of the mPIEZO1 reversal potential. Top: Average linear current-voltage (I-V) relationship as measured by whole-cell poke electrophysiology, as in panel F. Reversal potential is the voltage at which the current intercepts the x-axis for mouse *Piezo1* co-transfected with an empty vector (gray) or *CADM1* (turquoise). Bottom: mPIEZO1 reversal potentials for individual cells included in the average shown in the top panel, showing no statistical difference between the empty control and CADM1 (mean ± SEM, empty: 5 ± 3 mV, n = 8; CADM1: 8 ± 3 mV, n = 11, $p = 0.5$). I) mPIEZO1 recovery from inactivation as measured by whole-cell poke electrophysiology in the presence of empty vector control or CADM1 in HEK293T PIEZO1 KO cells. Cells were subjected to a two-step indentation protocol of 100 ms with an interstimulus interval ranging from 10 ms to 30 s. The magnitude of the second response (R2) was measured as a percentage of the first response (R1) at 8 time intervals, as shown for the representative traces in Panel J. Summary data are shown with R2/R1 shown as a function of the interstimulus interval. Fitting of this function indicates no difference between mPIEZO1 with an emtpy vector control vs. CADM1 (recovery time constant: PIEZO1 + empty, 1.1 ± 0.2 s, n = 4; PIEZO1 + CADM1, 1.3 ± 0.2 s, n = 5). J) Representative recovery from inactivation traces. The x-axis is shown on a base-10 log scale for visualization of all interstimulus intervals in the same plot for a single cell. R1 values are normalized to a single peak value for easier visualization of the current recovery.

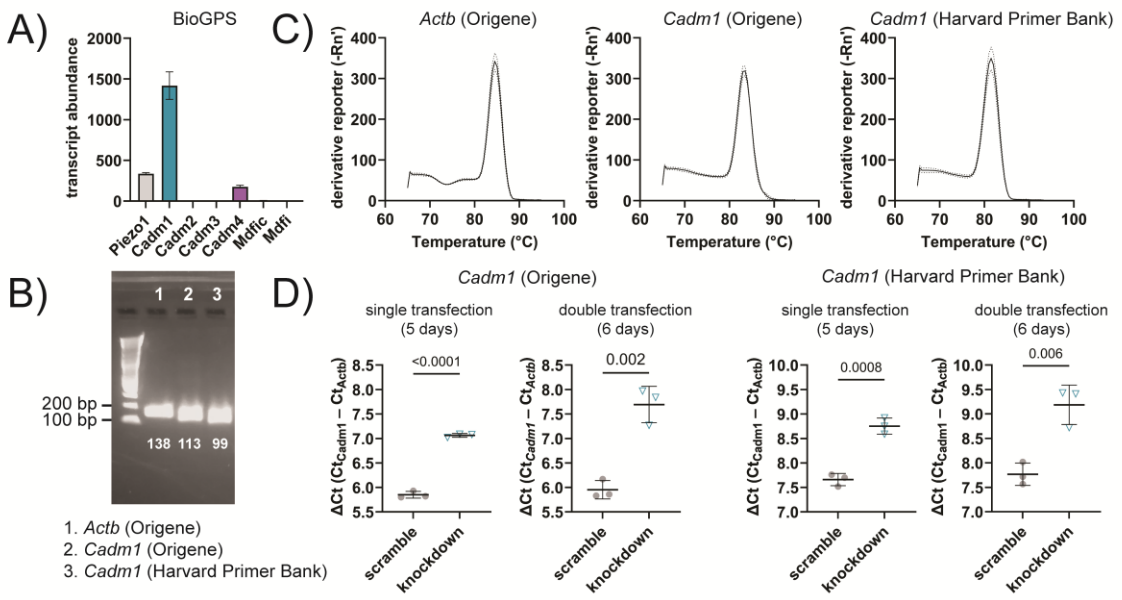

Fig. S4. *Cadm1* knockdown primer validation and qPCR in N2a cells. A) Relative abundance of *Piezo1* transcript compared to *Cadm1–4*, *Mdfic*, and *Mdfi* in N2a cells, as reported in the BioGPS database (mean ± SD)(13). B) Visualization of DNA amplicons on a 2% agarose gel, showing a single PCR product of the expected length for each of the qPCR primers used in this study. C) Representative derivative melt curves for *Actb* and *Cadm1* primers, showing the mean ± SD for 3 technical replicates of a single sample. D) Quantification of *Cadm1* siRNA knockdown by two different primer pairs (Origene or Harvard Primer Bank) under two different transfection conditions. Each point on the plot represents the average of three technical replicates for one biological replicate. The mean ± SD of the ΔC_t values for three biological replicates (non-targeting scramble control siRNA or *Cadm1* knockdown condition) are shown in each plot. Significance is shown as a *p*-value from an unpaired *t*-test. Similar knockdown efficiencies were obtained by each of the *Cadm1* primer pairs and correspond to ~53–57% transcript knockdown 5 days after a single transfection with siRNA or ~62–70% transcript knockdown 6 days after two transfections (3 days apart). The double transfection protocol was employed for all electrophysiology experiments in N2a cells. The pooled knockdown efficiency is likely an underestimate of the true knockdown efficiency in single cells, given that not every cell is transfected. Only cells containing a fluorescent transfection marker were included in electrophysiology experiments.

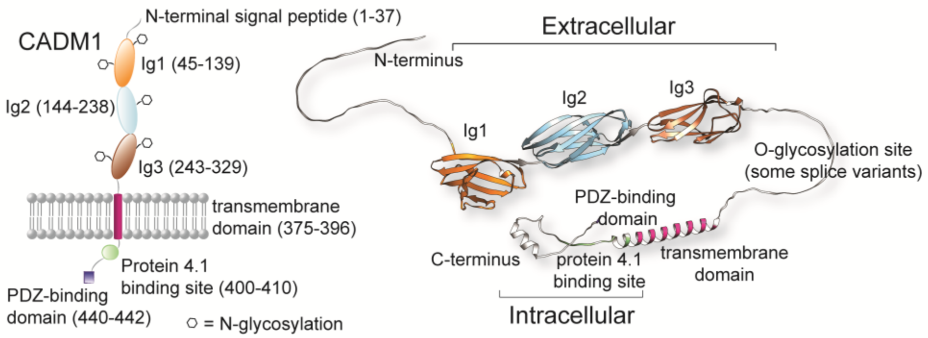


```
sp|Q9BY67|CADM1_HUMAN      MASVVLPSGSQCAAAAAAAAPPGLRLRLLLLLFS----AAALIPTGDGQNLFTKDVTVIE   56
sp|Q8N3J6|CADM2_HUMAN      ------------------MIWKR---SAVLRFYSVCG---LLLQGSQGQFPLTQNVTVVE   36
sp|Q8N126|CADM3_HUMAN      ------------------MGAPAASLLLLLLLFACCWAPGGANLSQDDSQPWTSDETVVA   42
sp|Q8NFZ8|CADM4_HUMAN      ------------------MGRARRFQWPLLLLWA------AAAGPGAGQEVQTENVTVAE   36
                                                         :* :::              ..   *.: **
                                                          Ig1
sp|Q9BY67|CADM1_HUMAN      GEVATISCQVNKSDDSVIQLLNPNRQTIYFRDFRPLKDSRFQLLNFSSSELKVSLTNVSI   116
sp|Q8N3J6|CADM2_HUMAN      GGTAILTCRVDQNDNTSLQWSNPAQQTLYFDDKKALRDNRIELVRASWHELSISVSDVSL   96
sp|Q8N126|CADM3_HUMAN      GGTVVLKCQVKDHEDSSLQWSNPAQQTLYFGEKRALRDNRIQLVTSTPHELSISISNVAL   102
sp|Q8NFZ8|CADM4_HUMAN      GGVAEITCRLHQYDGSIVVIQNPARQTLFFNGTRALKDERFQLEEFSPRRVRIRLSDARL   96
                           *  .. :.*::.. :.: :     ** :**::*    : *:*.*::*    :  .: : :::. :

sp|Q9BY67|CADM1_HUMAN      SDEGRYFCQLYTDPPQESYTTITVLVPPRNLMIDIQKDTAVEGEEIEVNCTAMASKPATT   176
sp|Q8N3J6|CADM2_HUMAN      SDEGQYTCSLFTMPVKTSKAYLTVLGVPEKPQISGFSSPVMEGDLMQLTCKTSGSKPAAD   156
sp|Q8N126|CADM3_HUMAN      ADEGEYTCSIFTMPVRTAKSLVTVLGIPQKPIITGYKSSLREKDTATLNCQSSGSKPAAR   162
sp|Q8NFZ8|CADM4_HUMAN      EDEGGYFCQLYTEDTHHQIATLTVLVAPENPVVEVR-EQAVEGGEVELSCLVPRSRPAAT   155
                            *** * *.::*    :    : :***  *.:   :     .    *      :.*     *:**:
                                                        Ig2
sp|Q9BY67|CADM1_HUMAN      IRWFKGNTELKGKSEV---EEWSDMYTVTSQLMLKVHKEDDGVPVICQVEHPAVT-GNLQ   232
sp|Q8N3J6|CADM2_HUMAN      IRWFKNDKEIKDVKYLKEEDANRKTFTVSSTLDFRVDRSDDGVAVICRVDHESLN-ATPQ   215
sp|Q8N126|CADM3_HUMAN      LTWRKGDQELHGEPTRIQEDPNGKTFTVSSSVTFQVTREDDGASIVCSVNHESLK-GADR   221
sp|Q8NFZ8|CADM4_HUMAN      LRWYRDRKELKGVSSS---QENGKVWSVASTVRFRVDRKDDGGIIICEAQNQALPSGHSK   212
                           : * :.   *::.          :    . ::*:* : ::* :.***  ::* .:: ::   .  :

sp|Q9BY67|CADM1_HUMAN      -TQRYLEVQYKPQVHIQMTYPLQGLTREGDALELTCEAIGKPQPVMVTWVRVDDEMPQ--   289
sp|Q8N3J6|CADM2_HUMAN      VAMQVLEIHYTPSVKIIP---STPFPQEGQPLILTCESKGKPLPEPVLWTKDGGELPDPD   272
sp|Q8N126|CADM3_HUMAN      STSQRIEVLYTPTAMIRP---DPPHPREGQKLLLHCEGRGNPVPQQYLWEKEGSVPPL--   276
sp|Q8NFZ8|CADM4_HUMAN      QTQYVLDVQYSPTARIHA---SQAVVREGDTLVLTCAVTGNPRPNQIRWNRGNESLPE--   267
                            :   ::: *.*  .  *          :**: *  *  *    *:* *     *  :  .    *
                                           Ig3
sp|Q9BY67|CADM1_HUMAN      HAVLSGPNLFINNLNKTDNGTYRCEASNIVGKAHSDYMLYVYDPPTTIPPPTTTTTTTTT   349
sp|Q8N3J6|CADM2_HUMAN      RMVVSGRELNILFLNKTDNGTYRCEATNTIGQSSAEYVLIVHDVPNTLLPTTIIPSLTTA   332
sp|Q8N126|CADM3_HUMAN      -KMTQESALIFPFLNKSDSGTYGCTATSNMGSYKAYYTLNVND-----------------   318
sp|Q8NFZ8|CADM4_HUMAN      RAEAVGETLTLPGLVSADNGTYTCEASNKHGHARALYVLVVYDPGAV-------------   314
                                   * :   *  .:*.*** * *:.   *    : * * * *
                                                                                 TM
sp|Q9BY67|CADM1_HUMAN      TTTTILTIITDS----------RAGEEGSIRAVDHAVIGGVVAVVVFAMLCLLIILGRYF   399
sp|Q8N3J6|CADM2_HUMAN      TVTTTVAITTSPTTSATTSSIRDPNALAGQNGPDHALIGGIVAVVVFVTLCSIFLLGRYL   392
sp|Q8N126|CADM3_HUMAN      -----------------------PSPVPSSSSTYHAIIGGIVAFIVFLLLIMLIFLGHYL   355
sp|Q8NFZ8|CADM4_HUMAN      --------------------------VEAQTSVPYAIVGGILALLVFLIICVLVGMVWCS   348
                                                    .   .  :*::**::*.:**   :   :. :
                      protein 4.1 site                          PDZ binding domain
sp|Q9BY67|CADM1_HUMAN      ARHKGTYFTHEAKGADDAADADTAIINAEGGQNNSEEKKEYFI      442
sp|Q8N3J6|CADM2_HUMAN      ARHKGTYLTNEAKGAEDAPDADTAIINAEGSQVNAEEKKEYFI      435
sp|Q8N126|CADM3_HUMAN      IRHKGTYLTHEAKGSDDAPDADTAIINAEGGQSGGDDKKEYFI      398
sp|Q8NFZ8|CADM4_HUMAN      VRQKGSYLTHEASGLDEQGEAREAFLNGSDG---HKRKEEFFI      388
                            *:**:*:*:**.*  ::   :*   *::*....     . *:*:**
```

Fig. S5. Sequence conservation across the cell adhesion molecule (CADM) family. Sequence alignments for human CADM1–4 are colored to match the cartoon and protein structure shown at the top. The red lines below the sequence indicate the regions of CADM1 that are absent in the ΔIg/ΔCT mutant.

Table S1. Whole-cell 'poke' electrophysiology screen of PIEZO1 interaction candidates. Mean inactivation time constant (tau), inward peak current (I_{max}), apparent mechanical threshold, and percent of peak current remaining at the end of a mechanical poke stimulus ± SEM at –80 mV, as shown in Fig. 2C and Appendix I. *P*-values are calculated from an unpaired two-tailed *t*-test comparing HEK293T PIEZO1 knockout cells transfected with mouse *Piezo1*-IRES-GFP and each candidate gene or an empty control vector containing a fluorescent marker. Each candidate was compared to the empty control vector transfected on the same day as each candidate for comparison, using similar numbers of individual cells ('n') and identical experiment conditions. Candidates showing any statistical trend or significance on a single day of recording were repeated on at least one additional day for confirmation of results. One cell for CADM1 and two cells for GPC4 revealed little inactivation during the stimulus (>125 ms, no exponential could be fit) and were not included in statistical analysis of tau. This was not observed in any of the other conditions and is consistent with the slow inactivation phenotype that we observe for PIEZO1 under these two conditions.

**Inactivation time constant (tau)**						
**candidate gene**	tau (ms) empty	tau (ms) candidate	*p*-value	significance	'n' empty	'n' candidate
**ADAM10**	13 ± 2	14 ± 3	0.8	ns	10	6
**ALCAM**	14 ± 1	16 ± 2	0.3	ns	13	10
**CADM1**	11.9 ± 0.5	27 ± 2	<0.0001	****	62	54
**CADM4**	14 ± 1	22 ± 4	0.04	*	15	11
**CDH2**	13 ± 2	12 ± 2	0.7	ns	10	8
**CXADR**	13 ± 1	16 ± 1	0.02	*	11	14
**Dsg2**	13 ± 2	11 ± 2	0.5	ns	10	7
**F11R**	14 ± 1	17 ± 2	0.2	ns	13	13
**GPC4**	12.5 ± 0.7	20 ± 2	0.0002	***	51	38
**ITGA6**	12 ± 1	11 ± 1	0.6	ns	19	11
**JAM3**	13 ± 1	15 ± 2	0.3	ns	15	8
**LMAN2**	11.6 ± 0.6	16 ± 2	0.07	ns	16	16
**NECTIN2**	13 ± 1	17 ± 2	0.1	ns	15	7
**OCLN**	13 ± 1	13 ± 1	0.8	ns	16	12
**PTK7**	13 ± 2	14 ± 2	0.8	ns	17	6
**PTPRF**	14 ± 2	16 ± 2	0.4	ns	15	10
**TMEM30A**	11.2 ± 0.6	10 ± 1	0.2	ns	22	11
**Apparent maximal current (I_{max})**						
**candidate gene**	I_{max} (nA) empty	I_{max} (nA) candidate	*p*-value	significance	'n' empty	'n' candidate
**ADAM10**	–1.5 ± 0.3	–1.1 ± 0.4	0.5	ns	10	6
**ALCAM**	–1.1 ± 0.2	–2.5 ± 0.8	0.07	ns	13	10
**CADM1**	–1.5 ± 0.2	–1.7 ± 0.2	0.5	ns	58	53
**CADM4**	–0.7 ± 0.1	–0.7 ± 0.2	0.7	ns	11	10
**CDH2**	–1.5 ± 0.3	–1.7 ± 0.4	0.6	ns	10	7
**CXADR**	–1.4 ± 0.3	–3.2 ± 0.4	0.003	**	10	14
**Dsg2**	–1.5 ± 0.3	–1.7 ± 0.3	0.6	ns	10	7
**F11R**	–1.1 ± 0.2	–2.9 ± 0.6	0.01	**	13	12
**GPC4**	–1.4 ± 0.1	–3.3 ± 0.3	<0.0001	****	46	39
**ITGA6**	–2.5 ± 0.6	–1.9 ± 0.4	0.4	ns	16	11
**JAM3**	–1.3 ± 0.2	–1.8 ± 0.4	0.2	ns	14	7
**LMAN2**	–1.6 ± 0.4	–1.8 ± 0.3	0.7	ns	16	16
**NECTIN2**	–1.0 ± 0.2	–1.5 ± 0.6	0.3	ns	13	6
**OCLN**	–1.3 ± 0.2	–2.7 ± 0.7	0.03	*	14	10

**PTK7**	–1.3 ± 0.2	–1.0 ± 0.2	0.4	ns	15	6
**PTPRF**	–1.1 ± 0.2	–1.5 ± 0.2	0.3	ns	14	10
**TMEM30A**	–1.7 ± 0.3	–1.0 ± 0.2	0.2	ns	20	8
**Apparent mechanical threshold (µm of indentation at first response)**						
**candidate gene**	threshold (µm) empty	threshold (µm) candidate	*p*-value	significance	‘n’ empty	‘n’ candidate
**ADAM10**	7 ± 1	8 ± 2	0.6	ns	11	6
**ALCAM**	10 ± 1	5 ± 1	0.003	**	14	10
**CADM1**	8.2 ± 0.4	7.0 ± 0.4	0.04	*	65	55
**CADM4**	8.5 ± 0.9	8 ± 1	0.9	ns	15	12
**CDH2**	7 ± 1	8 ± 1	0.4	ns	11	8
**CXADR**	9.5 ± 0.9	8 ± 1	0.2	ns	13	14
**Dsg2**	7 ± 1	6.5 ± 0.5	0.7	ns	11	7
**F11R**	10 ± 1	8 ± 1	0.1	ns	14	13
**GPC4**	6.7 ± 0.4	4.0 ± 0.4	<0.0001	****	53	40
**ITGA6**	9 ± 1	8.2 ± 0.8	0.4	ns	20	12
**JAM3**	11 ± 1	8 ± 2	0.1	ns	17	8
**LMAN2**	8.2 ± 0.8	8.1 ± 0.8	0.9	ns	16	19
**NECTIN2**	10 ± 1	9 ± 1	0.4	ns	16	8
**OCLN**	8.6 ± 0.9	10 ± 1	0.5	ns	16	13
**PTK7**	10 ± 1	8 ± 2	0.3	ns	18	7
**PTPRF**	8.5 ± 0.7	10 ± 1	0.4	ns	15	13
**TMEM30A**	9.4 ± 0.8	10 ± 1	0.9	ns	23	13
**Percent of maximal current (I_{max}) remaining at the end of the stimulus**						
**candidate gene**	% of I_{max} empty	% of I_{max} candidate	*p*-value	significance	‘n’ empty	‘n’ candidate
**ADAM10**	6 ± 4	18 ± 15	0.4	ns	10	6
**ALCAM**	4 ± 2	4 ± 2	0.8	ns	13	10
**CADM1**	2.8 ± 0.7	11 ± 2	0.0004	***	58	53
**CADM4**	6 ± 3	10 ± 5	0.4	ns	11	9
**CDH2**	6 ± 3	0.7 ± 0.3	0.2	ns	10	7
**CXADR**	2 ± 1	8 ± 5	0.3	ns	10	14
**Dsg2**	7 ± 4	3 ± 1	0.4	ns	10	7
**F11R**	4 ± 2	8 ± 6	0.4	ns	13	12
**GPC4**	4 ± 1	11 ± 3	0.01	*	46	39
**ITGA6**	6 ± 3	4 ± 1	0.7	ns	16	11
**JAM3**	3 ± 2	3 ± 1	1	ns	14	7
**LMAN2**	5 ± 2	3 ± 1	0.5	ns	16	16
**NECTIN2**	5 ± 2	2 ± 1	0.4	ns	13	6
**OCLN**	3 ± 1	2.2 ± 0.8	0.7	ns	14	10
**PTK7**	3 ± 1	8 ± 3	0.07	ns	15	6
**PTPRF**	3 ± 1	3.0 ± 0.6	0.8	ns	14	10
**TMEM30A**	4 ± 2	3 ± 1	0.7	ns	20	8

Table S2. Whole-cell 'poke' electrophysiology summary of glypican overexpression with mPIEZO1. Mean inactivation time constant (tau), peak current (I_{max}), apparent mechanical threshold, and percent of peak current remaining at the end of a mechanical poke stimulus ± SEM at –80 mV, as shown in Fig. S2. *P*-values are calculated from an unpaired two-tailed *t*-test comparing HEK293T PIEZO1 KO cells transfected with *Piezo1*-IRES-GFP and each GPC or an empty control vector containing a fluorescent marker. Experiments were performed with 4 independent transfections on 4 separate days. Each GPC was compared to the empty control vector transfected on the same day as each candidate for comparison, using similar numbers of individual cells ('n') and identical experiment conditions. Two cells for GPC4 revealed little inactivation during the stimulus (>125 ms, no exponential could be fit) and were not included in the statistical analysis of tau. This was not observed in any of the other conditions.

**Inactivation time constant (tau)**						
**candidate gene**	tau (ms) empty	tau (ms) candidate	*p*-value	significance	'n' empty	'n' candidate
**GPC1**	11.3 ± 0.8	21 ± 4	0.001	**	27	13
**GPC2**	11.3 ± 0.8	11 ± 2	0.9	ns	27	14
**GPC3**	11.3 ± 0.8	18 ± 3	0.004	**	27	11
**GPC4**	11.3 ± 0.8	24 ± 3	<0.0001	****	27	13
**GPC5**	11.3 ± 0.8	21 ± 2	<0.0001	****	27	11
**GPC6**	11.3 ± 0.8	19 ± 2	0.0003	***	27	10
**Apparent maximal current (I_{max})**						
**candidate gene**	I_{max} (nA) empty	I_{max} (nA) candidate	*p*-value	significance	'n' empty	'n' candidate
**GPC1**	–1.6 ± 0.2	–0.9 ± 0.3	0.05	ns	23	13
**GPC2**	–1.6 ± 0.2	–1.8 ± 0.3	0.6	ns	23	15
**GPC3**	–1.6 ± 0.2	–3.6 ± 0.7	0.002	**	23	11
**GPC4**	–1.6 ± 0.2	–3.8 ± 0.5	<0.0001	****	23	15
**GPC5**	–1.6 ± 0.2	–2.4 ± 0.5	0.07	ns	23	11
**GPC6**	–1.6 ± 0.2	–2.6 ± 0.8	0.1	ns	23	10
**Apparent mechanical threshold (µm of indentation at first response)**						
**candidate gene**	threshold (µm) empty	threshold (µm) candidate	*p*-value	significance	'n' empty	'n' candidate
**GPC1**	5.4 ± 0.5	4.5 ± 0.7	0.3	ns	29	14
**GPC2**	5.4 ± 0.5	2.6 ± 0.4	0.0004	***	29	15
**GPC3**	5.4 ± 0.5	2.3 ± 0.6	0.001	**	29	11
**GPC4**	5.4 ± 0.5	2.1 ± 0.4	<0.0001	****	29	14
**GPC5**	5.4 ± 0.5	4.5 ± 0.8	0.3	ns	29	11
**GPC6**	5.4 ± 0.5	2.5 ± 0.3	0.002	**	29	10
**Percent of maximal current (I_{max}) remaining at the end of the stimulus**						
**candidate gene**	% of I_{max} empty	% of I_{max} candidate	*p*-value	significance	'n' empty	'n' candidate
**GPC1**	5 ± 2	24 ± 6	0.0005	***	22	13
**GPC2**	5 ± 2	11 ± 4	0.2	ns	22	15
**GPC3**	5 ± 2	13 ± 4	0.02	*	22	11
**GPC4**	5 ± 2	22 ± 5	0.001	**	22	15
**GPC5**	5 ± 2	10 ± 5	0.2	ns	22	11
**GPC6**	5 ± 2	9 ± 5	0.3	ns	22	10

Table S3. Effect of CADM1 on mPIEZO2. Whole-cell poke electrophysiology data are shown as mean ± SEM at –80 mV. *P*-values are calculated from an unpaired two-tailed *t*-test comparing HEK293T PIEZO1 knockout cells transfected with mouse *Piezo2*-IRES-mNeonGreen and CADM1 (wild-type) or an empty control vector containing a fluorescent marker. Each candidate was compared to the empty control vector transfected on the same day as each candidate for comparison, using similar numbers of individual cells ('n') and identical experiment conditions. Statistical analysis represents pooled data from 4 independent transfections (4 separate recording days).

'poke' parameter	PIEZO2 + empty control	PIEZO2 + CADM1	*p*-value	significance	'n' empty	'n' CADM1
$tau_{inactivation}$ (ms)	5.7 ± 0.4	6.1 ± 0.5	0.6	ns	25	22
I_{max} (nA)	–0.8 ± 0.1	–0.9 ± 0.1	0.6	ns	23	19
threshold (μm)	6.9 ± 0.6	6.9 ± 0.6	1	ns	19	16
remaining current (% of I_{max})	1.1 ± 0.3	2.5 ± 0.5	0.02	*	23	19

Table S4. Effect of CADM1 overexpression on endogenous mPIEZO1 in N2a cells. Whole-cell poke electrophysiology data are shown as mean ± SEM at –80 mV. *P*-values are calculated from an unpaired two-tailed *t*-test comparing endogenous mPIEZO1 currents in cells transfected with *CADM1*-IRES-tdTomato or with those expressing an empty control vector containing a fluorescent marker. CADM1-expressing cells were compared to the empty control vector transfected on the same day, using similar numbers of individual cells ('n') and identical experiment conditions. Statistical analysis represents pooled data from 3 independent transfections/recording days.

'poke' parameter	PIEZO1 + empty control	PIEZO1 + CADM1	*p*-value	significance	'n' empty	'n' CADM1
$tau_{inactivation}$ (ms)	12.2 ± 0.8	22 ± 2	0.0006	***	14	17
I_{max} (nA)	–0.2 ± 0.05	–0.2 ± 0.02	0.6	ns	15	17
threshold (µm)	2.4 ± 0.5	3.7 ± 0.5	0.09	ns	15	17
remaining current (% of I_{max})	1.1 ± 0.9	9 ± 2	0.01	*	14	17

Table S5. Effect of *Cadm1* knockdown on endogenous mPIEZO1 in N2a cells. Whole-cell 'poke' electrophysiology data are shown as mean ± SEM at –80 mV. *P*-values are calculated from an unpaired two-tailed *t*-test comparing endogenous mPIEZO1 currents in cells transfected with either a non-targeting control RNA or si*Cadm1* and co-transfected with an empty vector containing a fluorescent marker. Statistical analysis represents pooled data from 3 independent transfections (3 separate recording days).

**'poke' parameter**	**PIEZO1 + non-targeting control**	**PIEZO1 + si*Cadm1***	***p*-value**	**significance**	**'n' control**	**'n' si*Cadm1***
$tau_{inactivation}$ (ms)	11.0 ± 0.7	8.8 ± 0.5	0.01	*	34	35
I_{max} (nA)	–0.26 ± 0.03	–0.26 ± 0.03	0.9	ns	32	30
threshold (µm)	7.3 ± 0.5	7.1 ± 0.4	0.8	ns	46	43
remaining current (% of I_{max})	2.3 ± 0.7	1.0 ± 0.2	0.1	ns	32	30

Table S6. Whole-cell 'poke' electrophysiology evaluation of CADM1 mutants co-transfected with PIEZO1. Mean inactivation time constant (tau, as shown in Fig. 4B), inward peak current (I_{max}), apparent mechanical threshold, and percent of peak current remaining at the end of a mechanical poke stimulus ± SEM at –80 mV. *P*-values are calculated from an unpaired two-tailed *t*-test comparing HEK293T PIEZO1 knockout cells transfected with mouse *Piezo1*-IRES-GFP and CADM1 (wild-type, WT or mutant) or an empty control vector containing a fluorescent marker. Each candidate was compared to the empty control vector transfected on the same day as each candidate for comparison, using similar numbers of individual cells ('n') and identical experiment conditions. Statistical analysis represents pooled data from 2–4 independent transfections (2–4 separate recording days). Empty control and WT CADM1 were recorded on every experiment day to ensure day-to-day consistency for better comparison to the mutants. The experimenter was blinded to the identity of the conditions until after data analyses were performed. None of the CADM1 mutants were significantly different from wild-type CADM1 (ΔIg, p = 0.5; ΔCT, p = 0.7; ΔPDZ, p = 0.5; ΔIg/ΔCT, p = 0.2).

**Inactivation time constant (tau)**						
**candidate gene**	tau (ms) empty	tau (ms) candidate	*p*-value	significance	'n' empty	'n' candidate
**CADM1 (WT)**	12.3 ± 0.6	24 ± 2	<0.0001	****	40	32
**CADM1 (ΔIg)**	12.0 ± 0.8	21 ± 3	0.0005	***	21	12
**CADM1 (ΔCT)**	12.0 ± 0.8	24 ± 2	<0.0001	****	21	10
**CADM1 (ΔPDZ)**	12.0 ± 0.8	21 ± 2	<0.0001	****	21	9
**CADM1 (ΔIg/ΔCT)**	12.6 ± 0.9	23 ± 1	<0.0001	****	19	15
**Apparent maximal current (I_{max})**						
**candidate gene**	I_{max} (nA) empty	I_{max} (nA) candidate	*p*-value	significance	'n' empty	'n' candidate
**CADM1 (WT)**	–1.6 ± 0.2	–1.6 ± 0.2	0.7	ns	37	31
**CADM1 (ΔIg)**	–2.0 ± 0.3	–2.0 ± 0.3	0.9	ns	20	12
**CADM1 (ΔCT)**	–2.0 ± 0.3	–3.2 ± 0.9	0.1	ns	20	8
**CADM1 (ΔPDZ)**	–2.0 ± 0.3	–1.6 ± 0.3	0.5	ns	20	9
**CADM1 (ΔIg/ΔCT)**	–1.3 ± 0.3	–2.2 ± 0.5	0.1	ns	17	15
**Apparent mechanical threshold (µm of indentation at first response)**						
**candidate gene**	threshold (µm) empty	threshold (µm) candidate	*p*-value	significance	'n' empty	'n' candidate
**CADM1 (WT)**	8.0 ± 0.5	7.5 ± 0.6	0.5	ns	40	33
**CADM1 (ΔIg)**	7.9 ± 0.5	7 ± 1	0.5	ns	21	13
**CADM1 (ΔCT)**	7.9 ± 0.5	6.7 ± 0.9	0.2	ns	21	10
**CADM1 (ΔPDZ)**	7.9 ± 0.5	7.7 ± 0.8	0.8	ns	21	9
**CADM1 (ΔIg/ΔCT)**	8.2 ± 0.9	7.0 ± 0.9	0.4	ns	19	15
**Percent of maximal current (I_{max}) remaining at the end of the stimulus**						
**candidate gene**	% of I_{max} empty	% of I_{max} candidate	*p*-value	significance	'n' empty	'n' candidate
**CADM1 (WT)**	2.9 ± 0.8	10 ± 3	0.006	**	37	31
**CADM1 (ΔIg)**	1.8 ± 0.5	13 ± 5	0.01	*	20	12
**CADM1 (ΔCT)**	1.8 ± 0.5	12 ± 2	<0.0001	****	20	8
**CADM1 (ΔPDZ)**	1.8 ± 0.5	10 ± 2	<0.0001	****	20	9
**CADM1 (ΔIg/ΔCT)**	4 ± 2	5 ± 1	0.7	ns	17	15

Appendix I: Electrophysiology screen. Whole-cell poke electrophysiology data of all candidates listed in Table S1, shown alphabetically. Left to right: inactivation time constant (tau), I_{max}, apparent threshold, and % current remaining at the end of the mechanical poking stimulus.

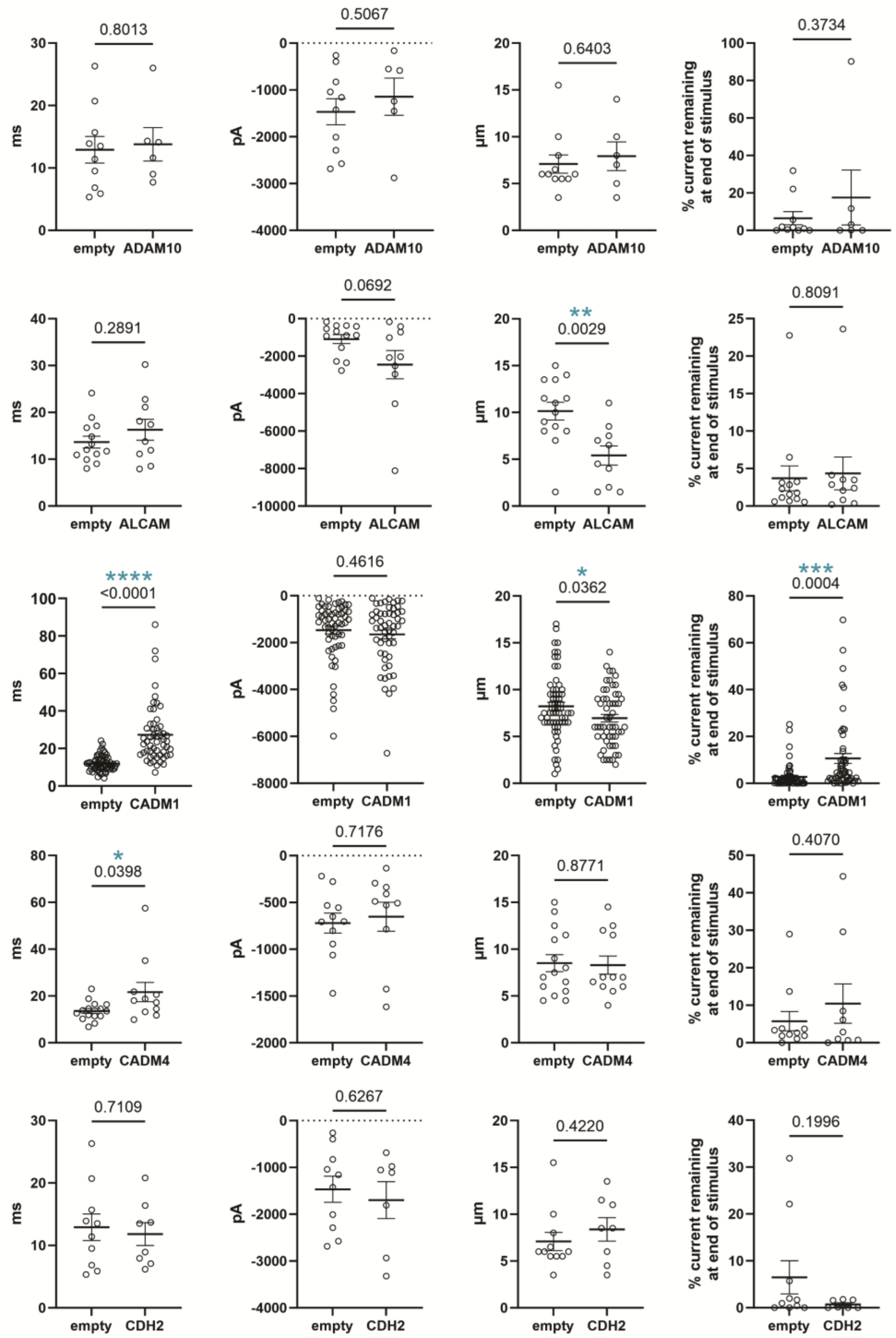

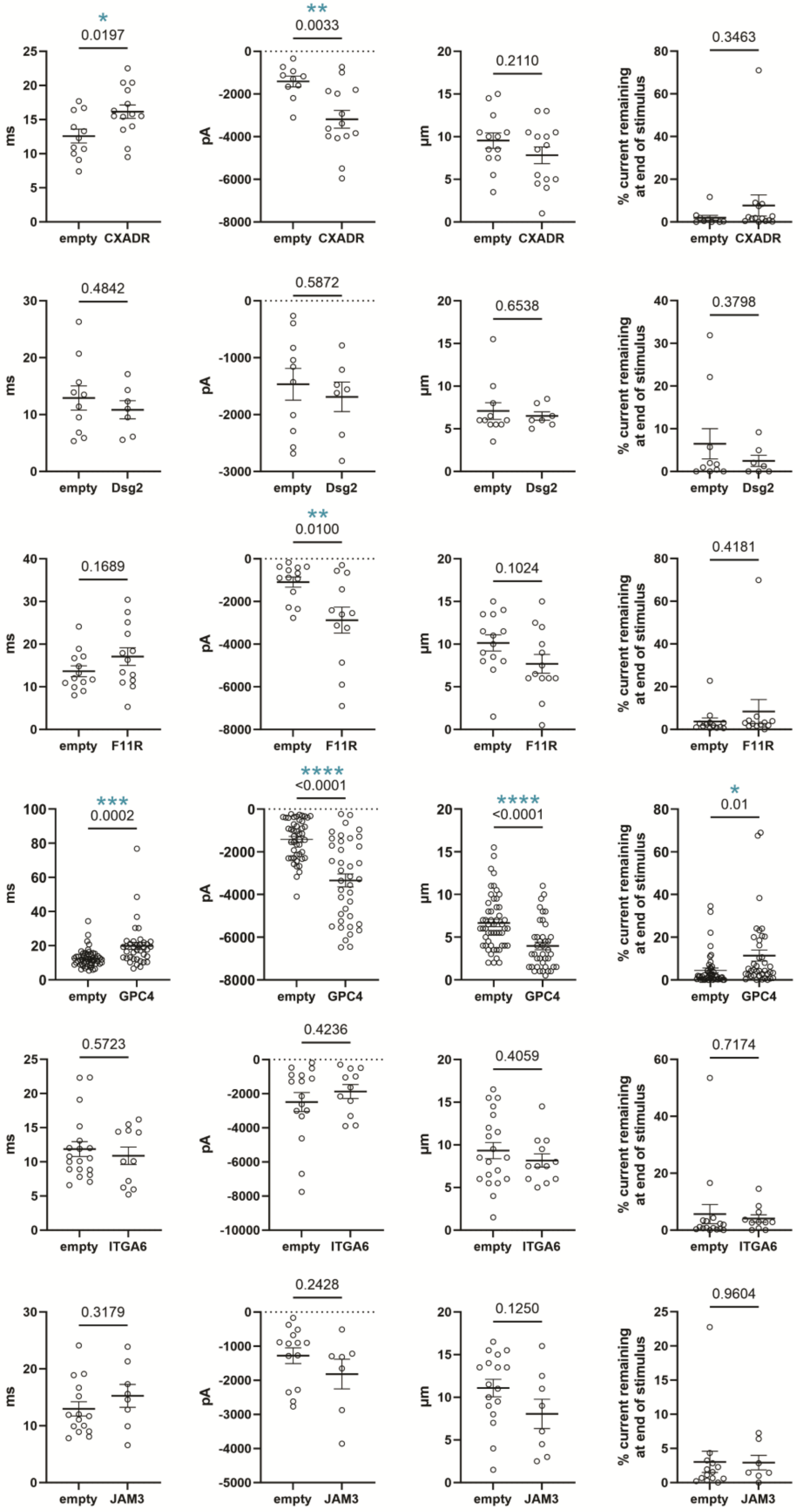

*
0.0197
ms
empty CXADR
**
0.0033
pA
empty CXADR
0.2110
µm
empty CXADR
0.3463
% current remaining at end of stimulus
empty CXADR
0.4842
ms
empty Dsg2
0.5872
pA
empty Dsg2
0.6538
µm
empty Dsg2
0.3798
% current remaining at end of stimulus
empty Dsg2
0.1689
ms
empty F11R
**
0.0100
pA
empty F11R
0.1024
µm
empty F11R
0.4181
% current remaining at end of stimulus
empty F11R
***
0.0002
ms
empty GPC4
****
<0.0001
pA
empty GPC4
****
<0.0001
µm
empty GPC4
*
0.01
% current remaining at end of stimulus
empty GPC4
0.5723
ms
empty ITGA6
0.4236
pA
empty ITGA6
0.4059
µm
empty ITGA6
0.7174
% current remaining at end of stimulus
empty ITGA6
0.3179
ms
empty JAM3
0.2428
pA
empty JAM3
0.1250
µm
empty JAM3
0.9604
% current remaining at end of stimulus
empty JAM3

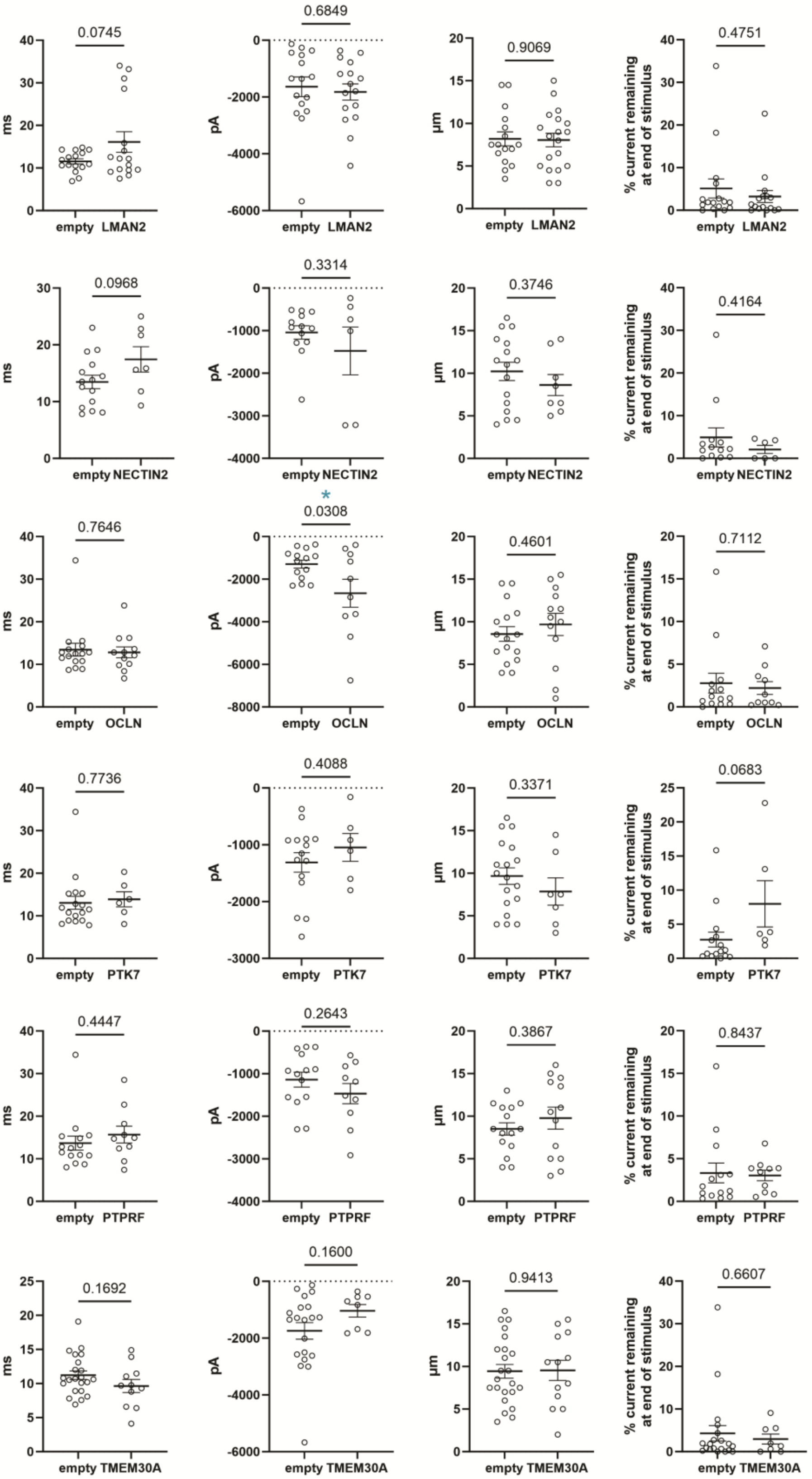

0.0745
ms
empty LMAN2
0.6849
pA
empty LMAN2
0.9069
µm
empty LMAN2
0.4751
% current remaining at end of stimulus
empty LMAN2
0.0968
ms
empty NECTIN2
0.3314
pA
empty NECTIN2
0.3746
µm
empty NECTIN2
0.4164
% current remaining at end of stimulus
empty NECTIN2
0.7646
ms
empty OCLN
*
0.0308
pA
empty OCLN
0.4601
µm
empty OCLN
0.7112
% current remaining at end of stimulus
empty OCLN
0.7736
ms
empty PTK7
0.4088
pA
empty PTK7
0.3371
µm
empty PTK7
0.0683
% current remaining at end of stimulus
empty PTK7
0.4447
ms
empty PTPRF
0.2643
pA
empty PTPRF
0.3867
µm
empty PTPRF
0.8437
% current remaining at end of stimulus
empty PTPRF
0.1692
ms
empty TMEM30A
0.1600
pA
empty TMEM30A
0.9413
µm
empty TMEM30A
0.6607
% current remaining at end of stimulus
empty TMEM30A

Appendix II: cDNA library information. List of candidate genes used for constructs in this study and their commercial sources. Sequences were subcloned from the commercial vectors into expression vectors via Gateway recombination or PCR-based cloning as described in 'Materials and Methods'. If the gene did not express, this is indicated in the 'Notes' column. If purchased sequences deviate from those reported in UniProt, this has been noted below each protein sequence following the main table. Protein sequences are provided following the main table.

Gene	UniProt ID	cDNA source (company)	cDNA source (cat#)	Backbone(s)	Notes
**ADAM10**	O14672-1	Addgene	Plasmid #65106	IRES-tdTomato	Gschwind, et al. *EMBO J.* 2003, *22(10)*:2411
**ALCAM**	Q13740-1	Horizon Discovery, hORFeome v8.1	OHS6084-202634686	IRES-tdTomato, C-term mCherry (Addgene Plasmid #31907)	
**ANO6**	Q4KMQ2-1	Horizon Discovery, hORFeome v8.1	OHS6084-202637663	C-term 2HA (Addgene Plasmid #118374)	*no expression*
**Ano6**	Q6P9J9-1	Addgene	Plasmid #62554	C-term mCherry	used as purchased
**ATP1B1**	P05026-1	Horizon Discovery, hORFeome v8.1	OHS6084-202629350	C-term mCherry (Addgene Plasmid #31907)	
**BCAM**	P50895	Horizon Discovery, hORFeome v8.1	OHS6084-202630130	C-term mCherry (Addgene Plasmid #31907)	
**CADM1**	Q9BY67-1	GenScript	gene synthesis	IRES-tdTomato	mammalian codon-optimized
**CADM1**	A0A4Z1	Horizon Discovery, hORFeome v8.1	OHS6084-202639433	C-term mCherry (Addgene Plasmid #31907), IRES-tdTomato	Partially truncated commercial sequence used for initial Ca^{2+} imaging screens; canonical protein (Q9BY67-1) in an IRES vector was used for all subsequent work
**CADM1 ΔCT**	N/A	N/A	PCR-based mutagenesis	IRES-tdTomato	Mutated from canonical (Q9BY67-1)
**CADM1 ΔIg**	N/A	N/A	PCR-based mutagenesis	IRES-tdTomato	Mutated from canonical (Q9BY67-1)
**CADM1 ΔIg/ΔCT**	N/A	N/A	PCR-based mutagenesis	IRES-tdTomato	Mutated from canonical (Q9BY67-1)
**CADM1 ΔPDZ**	N/A	N/A	PCR-based mutagenesis	IRES-tdTomato	Mutated from canonical (Q9BY67-1)
**CADM4**	Q8NFZ8	Horizon Discovery, ORFeome Collab.	OHS5893-202501989	IRES-mCherry	
**CD55**	P08174-1	Horizon Discovery, hORFeome v8.1	OHS6084-202629631	C-term 2HA, C-term mCherry (Addgene	*no expression*

				#118374, #31907)	
**CD99L2**	Q8TCZ2-1	Horizon Discovery, hORFeome v8.1	OHS6084-202633259	C-term mCherry (Addgene Plasmid #31907)	Overexpression made cells unhealthy
**CDH2**	P19022-1	Horizon Discovery, MGC	MHS6278-202808064	IRES-mCherry	mutations in commercial gene were corrected to the canonical sequence
**CXADR**	P78310-1	Horizon Discovery, hORFeome v8.1	OHS6084-202634893	IRES-tdTomato, C-term mCherry (Addgene Plasmid #31907)	
**Dsg2**	O55111	Horizon Discovery, MGC	MMM1013-211694124	IRES-tdTomato	
**F11R**	Q9Y624-1	Horizon Discovery, hORFeome v8.1	OHS6084-202632205	IRES-tdTomato, C-term mCherry (Addgene Plasmid #31907)	
**GPC1**	P35052-1	Horizon Discovery, hORFeome v8.1	OHS6084-202635061	IRES-tdTomato	S500G natural variant
**GPC2**	Q8N158	Horizon Discovery, hORFeome v8.1	OHS5893-202494498	IRES-tdTomato	
**GPC3**	P51654-1	Horizon Discovery, hORFeome v8.1	OHS6084-202635045	IRES-tdTomato	mutation in commercial gene was corrected to the canonical sequence
**GPC4**	O75487-1	Horizon Discovery, hORFeome v8.1	OHS6084-202629760	IRES-tdTomato, C-term mCherry (Addgene Plasmid #31907)	
**GPC5**	P78333	Horizon Discovery, hORFeome v8.1	OHS6084-202629768	IRES-tdTomato	
**GPC6**	Q9Y625	Horizon Discovery, hORFeome v8.1	OHS1770-202317090	IRES-tdTomato	
**IGSF8**	Q969P0-1	Horizon Discovery, hORFeome v8.1	OHS6084-202637332	IRES-tdTomato, C-term mCherry (Addgene Plasmid #31907)	
**ITGA6**	P23229-2	Horizon Discovery, MGC	MHS6278-213243527	IRES-mCherry	
**ITGB1**	P05556-1	Horizon Discovery, MGC	MHS6278-202757317	IRES-mCherry	*weak expression*
**JAM3**	Q9BX67-1	Horizon Discovery, hORFeome v8.1	OHS6084-202633263	IRES-tdTomato, C-term mCherry (Addgene Plasmid #31907)	
**KIRREL**	Q96J84-1	Horizon Discovery, hORFeome v8.1	OHS6084-202632589	C-term mCherry (Addgene Plasmid #31907)	
**LMAN2**	Q12907	Horizon Discovery, hORFeome v8.1	OHS6084-202631660	IRES-tdTomato, C-term mCherry (Addgene Plasmid #31907)	

**LSR**	Q86X29-1	Horizon Discovery, hORFeome v8.1	OHS6084-202632381	C-term 2HA, C-term mCherry (Addgene #118374, #31907)	*weak expression*
**MCAM**	P43121-1	Horizon Discovery, hORFeome v8.1	OHS6084-202635261	C-term mCherry (Addgene Plasmid #31907)	
**MDFI**	Q99750	GenScript	gene synthesis	IRES-tdTomato	mammalian codon-optimized
**NCAM1**	P13591-2	Horizon Discovery, MGC	MHS6278-202802080	IRES-mCherry	Overexpression made cells unhealthy
**NCSTN**	Q92542-2	Horizon Discovery, hORFeome v8.1	OHS6084-202639411	C-term 2HA (Addgene Plasmid #118374)	*no expression*
**NECTIN2**	Q92692-2	Horizon Discovery, hORFeome v8.1	OHS6084-202630507	IRES-tdTomato, C-term mCherry (Addgene Plasmid #31907)	Transmembrane domain differs from canonical
**NPTN**	Q9Y639-1	Horizon Discovery, hORFeome v8.1	OHS6084-202632039	C-term mCherry (Addgene Plasmid #31907)	*weak expression*
**OCLN**	Q16625-1	Horizon Discovery, MGC	MHS6278-202800831	IRES-tdTomato	
**PODXL**	O00592-2	Horizon Discovery, hORFeome v8.1	OHS6084-202635394	C-term mCherry (Addgene Plasmid #31907)	*weak expression*
**PTK7**	Q13308-1	Horizon Discovery, MGC	MHS6278-202806264	IRES-mCherry	
**PTPRF**	P10586-2	Horizon Discovery, MGC	MHS6278-202758744	IRES-tdTomato	
**PVR**	P15151-1	Horizon Discovery, hORFeome v8.1	OHS6084-202635450	C-term mCherry (Addgene Plasmid #31907)	
**RTN4R**	Q9BZR6	Horizon Discovery, hORFeome v8.1	OHS6084-202632993	C-term 2HA (Addgene Plasmid #118374)	*no expression*
**SMPDL3B**	Q92485-2	Horizon Discovery, hORFeome v8.1	OHS6084-202632075	C-term mCherry (Addgene Plasmid #31907)	
**TMEM30A**	Q9NV96-2	Horizon Discovery, hORFeome v8.1	OHS6084-202632668	IRES-tdTomato, C-term mCherry (Addgene Plasmid #31907)	C-term construct showed an effect by Ca^{2+} imaging, but this was not reproduced by electrophysiology. The untagged IRES vector did not have an apparent effect on PIEZO1 by Ca^{2+} imaging or electrophysiology (Appendix II, III)

Appendix II, continued: Protein sequences for all constructs

ADAM10 (human canonical*, Uniprot ID O14672-1, 748 AA, 84.1 kDa):

MVLLRVLILLLSWAAGMGGQYGNPLNKYIRHYEGLSYNVDSLHQKHQRAKRAVSHEDQFSRLDFHAHGRHF
NLRMKRDTSLFSDEFKVETSNKVLGYDTSHIYTGHIYGEEGSFGHGSVIDGRFEGFIQTRGGTFYVEPAER
YIKDRTLPFHSVIYHEDDINYPHKYGPQGGCADHSVFERMRKYQMTGVEEVTQIPQEEHAANGPELLRKKR
TTSAEKNTCQLYIQTDHLFFKYYGTREAVIAQISSHVKAIDTIYQTTDFSGIRNISFMVKRIRINTTADEK
DPTNPFRFPNIGVEKFLELNSEQNHDDYCLAHVFTDRDFDDGVLGLAWVGAPSGSSGGICEKSKLYSDGKK
KSLNTGIITVQNYGSHVPPKVSHITFAHEVGHNFGSPHDSGTECTPGESKNLGQKENGNYIMYARATSGDK
LNNNKFSLCSIRNISQVLEKKRNNCFVESGQPICGNGMVEQGEECDCGYSDQCKDECCFDANQPEGRKCKL
KPGKQCSPSQGPCCTAQCAFKSKSEKCRDDSDCAREGICNGFTALCPASDPKPNFTDCNRHTQVCINGQCA
GSICEKYGLEECTCASSDGKDDKELCHVCCMKKMDPSTCASTGSVQWSRHFSGRTITLQPGSPCNDFRGYC
DVFMRCRLVDADGPLARLKKAIFSPELYENIAEWIVAHWWAVLLMGIALIMLMAGFIKICGVHTPSSNPKL
PPPKPLPGTLKRRRPPQPIQQPQRQRPRESYQMGHMRR

*mutations compared to canonical: L60S, D96G, S115G, Y316H, S700G; functionally characterized in Gschwind, et al. *EMBO J.* 2003, *22(10)*:2411(14)

ALCAM (human canonical, Uniprot ID Q13740-1, 583 AA, MW 65.1 kDa):

MESKGASSCRLLFCLLISATVFRPGLGWYTVNSAYGDTIIIPCRLDVPQNLMFGKWKYEKPDGSPVFIAFR
SSTKKSVQYDDVPEYKDRLNLSENYTLSISNARISDEKRFVCMLVTEDNVFEAPTIVKVFKQPSKPEIVSK
ALFLETEQLKKLGDCISEDSYPDGNITWYRNGKVLHPLEGAVVIIFKKEMDPVTQLYTMTSTLEYKTTKAD
IQMPFTCSVTYYGPSGQKTIHSEQAVFDIYYPTEQVTIQVLPPKNAIKEGDNITLKCLGNGNPPPEEFLFY
LPGQPEGIRSSNTYTLTDVRRNATGDYKCSLIDKKSMIASTAITVHYLDLSLNPSGEVTRQIGDALPVSCT
ISASRNATVVWMKDNIRLRSSPSFSSLHYQDAGNYVCETALQEVEGLKKRESLTLIVEGKPQIKMTKKTDP
SGLSKTIICHVEGFPKPAIQWTITGSGSVINQTEESPYINGRYYSKIIISPEENVTLTCTAENQLERTVNS
LNVSAISIPEHDEADEISDENREKVNDQAKLIVGIVVGLLLAALVAGVVYWLYMKKSKTASKHVNKDLGNM
EENKKLEENNHKTEA

ANO6 (human canonical*, Uniprot ID Q4KMQ2-1, 910 AA, MW 106.2 kDa):

MKKMSRNVLLQMEEEEDDDDGDIVLENLGQTIVPDLGSLESQHDFRTPEFEEFNGKPDSLFFNDGQRRIDF
VLVYEDESRKETNKKGTNEKQRRKRQAYESNLICHGLQLEATRSVLDDKLVFVKVHAPWEVLCTYAEIMHI
KLPLKPNDLKNRSSAFGTLNWFTKVLSVDESIIKPEQEFFTAPFEKNRMNDFYIVDRDAFFNPATRSRIVY
FILSRVKYQVINNVSKFGINRLVNSGIYKAAFPLHDCKFRRQSEDPSCPNERYLLYREWAHPRSIYKKQPL
DLIRKYYGEKIGIYFAWLGYYTQMLLLAAVVGVACFLYGYLNQDNCTWSKEVCHPDIGGKIIMCPQCDRLC
PFWKLNITCESSKKLCIFDSFGTLVFAVFMGVWVTLFLEFWKRRQAELEYEWDTVELQQEEQARPEYEARC
THVVINEITQEEERIPFTAWGKCIRITLCASAVFFWILLIIASVIGIIVYRLSVFIVFSAKLPKNINGTDP
IQKYLTPQTATSITASIISFIIIMILNTIYEKVAIMITNFELPRTQTDYENSLTMKMFLFQFVNYYSSCFY
IAFFKGKFVGYPGDPVYWLGKYRNEECDPGGCLLELTTQLTIIMGGKAIWNNIQEVLLPWIMNLIGRFHRV
SGSEKITPRWEQDYHLQPMGKLGLFYEYLEMIIQFGFVTLFVASFPLAPLLALVNNILEIRVDAWKLTTQF
RRLVPEKAQDIGAWQPIMQGIAILAVVTNAMIIAFTSDMIPRLVYYWSFSVPPYGDHTSYTMEGYINNTLS
IFKVADFKNKSKGNPYSDLGNHTTCRYRDFRYPPGHPQEYKHNIYYWHVIAAKLALIIVMEHVIYSVKFFI
SYAIPDVSKRTKSKIQREKYLTQKLLHENHLKDMTKNMGVIAERMIEAVDNNLRPKSE

*contains F837L point mutation relative to canonical

ANO6 (mouse canonical, Uniprot ID Q6P9J9-1, 911 AA, MW 106.3 kDa):

MQMMTRKVLLNMELEEDDDEDGDIVLENFDQTIVCPTFGSLENQQDFRTPEFEEFNGKPDSLFFTDGQRRI
DFILVYEDESKKENNKKGTNEKQKRKRQAYESNLICHGLQLEATRSVSDDKLVFVKVHAPWEVLCTYAEIM

HIKLPLKPNDLKTRSPFGNLNWFTKVLRVNESVIKPEQEFFTAPFEKSRMNDFYILDRDSFFNPATRSRIV
YFILSRVKYQVMNNVNKFGINRLVSSGIYKAAFPLHDCRFNYESEDISCPSERYLLYREWAHPRSIYKKQP
LDLIRKYYGEKIGIYFAWLGYYTQMLLLAAVVGVACFLYGYLDQDNCTWSKEVCDPDIGGQILMCPQCDRL
CPFWRLNITCESSKKLCIFDSFGTLIFAVFMGVWVTLFLEFWKRRQAELEYEWDTVELQQEEQARPEYEAQ
CNHVVINEITQEEERIPFTTCGKCIRVTLCASAVFFWILLIIASVIGIIVYRLSVFIVFSTTLPKNPNGTD
PIQKYLTPQMATSITASIISFIIIMILNTIYEKVAIMITNFELPRTQTDYENSLTMKMFLFQFVNYYSSCF
YIAFFKGKFVGYPGDPVYLLGKYRSEECDPGGCLLELTTQLTIIMGGKAIWNNIQEVLLPWVMNLIGRYKR
VSGSEKITPRWEQDYHLQPMGKLGLFYEYLEMIIQFGFVTLFVASFPLAPLLALVNNILEIRVDAWKLTTQ
FRRMVPEKAQDIGAWQPIMQGIAILAVVTNAMIIAFTSDMIPRLVYYWSFSIPPYGDHTYYTMDGYINNTL
SVFNITDFKNTDKENPYIGLGNYTLCRYRDFRNPPGHPQEYKHNIYYWHVIAAKLAFIIVMEHIIYSVKFF
ISYAIPDVSKITKSKIKREKYLTQKLLHESHLKDLTKNMGIIAERIGGTVDNSVRPKLE

ATP1B1 (human canonical, Uniprot ID P05026-1, 303 AA, 35.1 kDa):

MARGKAKEEGSWKKFIWNSEKKEFLGRTGGSWFKILLFYVIFYGCLAGIFIGTIQVMLLTISEFKPTYQDR
VAPPGLTQIPQIQKTEISFRPNDPKSYEAYVLNIVRFLEKYKDSAQRDDMIFEDCGDVPSEPKERGDFNHE
RGERKVCRFKLEWLGNCSGLNDETYGYKEGKPCIIIKLNRVLGFKPKPPKNESLETYPVMKYNPNVLPVQC
TGKRDEDKDKVGNVEYFGLGNSPGFPLQYYPYYGKLLQPKYLQPLLAVQFTNLTMDTEIRIECKAYGENIG
YSEKDRFQGRFDVKIEVKS

BCAM (human canonical, Uniprot ID P50895, 628 AA, 67.4 kDa):

MEPPDAPAQARGAPRLLLLAVLLAAHPDAQAEVRLSVPPLVEVMRGKSVILDCTPTGTHDHYMLEWFLTDR
SGARPRLASAEMQGSELQVTMHDTRGRSPPYQLDSQGRLVLAEAQVGDERDYVCVVRAGAAGTAEATARLN
VFAKPEATEVSPNKGTLSVMEDSAQEIATCNSRNGNPAPKITWYRNGQRLEVPVEMNPEGYMTSRTVREAS
GLLSLTSTLYLRLRKDDRDASFHCAAHYSLPEGRHGRLDSPTFHLTLHYPTEHVQFWVGSPSTPAGWVREG
DTVQLLCRGDGSPSPEYTLFRLQDEQEEVLNVNLEGNLTLEGVTRGQSGTYGCRVEDYDAADDVQLSKTLE
LRVAYLDPLELSEGKVLSLPLNSSAVVNCSVHGLPTPALRWTKDSTPLGDGPMLSLSSITFDSNGTYVCEA
SLPTVPVLSRTQNFTLLVQGSPELKTAEIEPKADGSWREGDEVTLICSARGHPDPKLSWSQLGGSPAEPIP
GRQGWVSSSLTLKVTSALSRDGISCEASNPHGNKRHVFHFGTVSPQTSQAGVAVMAVAVSVGLLLLVVAVF
YCVRRKGGPCCRQRREKGAPPPGEPGLSHSGSEQPEQTGLLMGGASGGARGGSGGFGDEC

CADM1 (human canonical, Uniprot ID Q9BY67-1, 442 AA, MW 48.5 kDa):

MASVVLPSGSQCAAAAAAAAPPGLRLRLLLLLFSAAALIPTGDGQNLFTKDVTVIEGEVATISCQVNKSDD
SVIQLLNPNRQTIYFRDFRPLKDSRFQLLNFSSSELKVSLTNVSISDEGRYFCQLYTDPPQESYTTITVLV
PPRNLMIDIQKDTAVEGEEIEVNCTAMASKPATTIRWFKGNTELKGKSEVEEWSDMYTVTSQLMLKVHKED
DGVPVICQVEHPAVTGNLQTQRYLEVQYKPQVHIQMTYPLQGLTREGDALELTCEAIGKPQPVMVTWVRVD
DEMPQHAVLSGPNLFINNLNKTDNGTYRCEASNIVGKAHSDYMLYVYDPPTTIPPPTTTTTTTTTTTTIL
TIITDSRAGEEGSIRAVDHAVIGGVVAVVVFAMLCLLIIILGRYFARHKGTYFTHEAKGADDAADADTAIIN
AEGGQNNSEEKKEYFI

CADM1 (human truncated isoform*, Uniprot ID A0A4Z1, 387 AA, MW 42.7 kDa):

MASVVLPSGSQCAAAAAAAAPPGLRLRLLLLLFSAAALIPTGDGQNLFTKDVTVIEGEVATISCQVNKSDD
SVIQLLNPNRQTIYFRDFRPLKDSRFQLLNFSSSELKVSLTNVSISDEGRYFCQLYTDPPQESYTTITVLV
PPRNLMIDIQKDTAVEGEEIEVNCTAMASKPATTIRWFKGNTELKGKSEVEEWSDMYTVTSQLMLKVHKED
DGVPVICQVEHPAVTGNLQTQRYLEVQYKPQVHIQMTYPLQGLTREGDALELTCEAIGKPQPVMVTWVRVD
DEMPQHAVLSGPNLFINNLNKTDNGTYRCEASNIVGKAHSDYMLYVYDSRAGEEGSIRAVDHAVIGGVVAV
VVFAMLCLLIIILGRYFARHKGLFSLTSSPRIK

*commercially available gene from ORFeome library that contains a C-terminal truncation; this sequence was used only for preliminary calcium imaging screening prior to gene synthesis and subcloning of the canonical sequence.

CADM1 ΔCT (C-terminal deletion mutant Δ401–442):

```
MASVVLPSGSQCAAAAAAAAPPGLRLRLLLLLFSAAALIPTGDGQNLFTKDVTVIEGEVATISCQVNKSDD
SVIQLLNPNRQTIYFRDFRPLKDSRFQLLNFSSSELKVSLTNVSISDEGRYFCQLYTDPPQESYTTITVLV
PPRNLMIDIQKDTAVEGEEIEVNCTAMASKPATTIRWFKGNTELKGKSEVEEWSDMYTVTSQLMLKVHKED
DGVPVICQVEHPAVTGNLQTQRYLEVQYKPQVHIQMTYPLQGLTREGDALELTCEAIGKPQPVMVTWVRVD
DEMPQHAVLSGPNLFINNLNKTDNGTYRCEASNIVGKAHSDYMLYVYDPPTTIPPPTTTTTTTTTTTTTIL
TIITDSRAGEEGSIRAVDHAVIGGVVAVVVFAMLCLLIILGRYFA
```

CADM1 ΔIg (Ig domain deletion mutant Δ59–316):

```
MASVVLPSGSQCAAAAAAAAPPGLRLRLLLLLFSAAALIPTGDGQNLFTKDVTVIEGEGTNIVGKAHSDYM
LYVYDPPTTIPPPTTTTTTTTTTTTTILTIITDSRAGEEGSIRAVDHAVIGGVVAVVVFAMLCLLIILGRY
FARHKGTYFTHEAKGADDAADADTAIINAEGGQNNSEEKKEYFI
```

CADM1 ΔIg/ΔCT:

```
MASVVLPSGSQCAAAAAAAAPPGLRLRLLLLLFSAAALIPTGDGQNLFTKDVTVIEGEGTNIVGKAHSDYM
LYVYDPPTTIPPPTTTTTTTTTTTTTILTIITDSRAGEEGSIRAVDHAVIGGVVAVVVFAMLCLLIILGRY
FA
```

CADM1 ΔPDZ (PDZ-binding domain deletion mutant Δ440–442):

```
MASVVLPSGSQCAAAAAAAAPPGLRLRLLLLLFSAAALIPTGDGQNLFTKDVTVIEGEVATISCQVNKSDD
SVIQLLNPNRQTIYFRDFRPLKDSRFQLLNFSSSELKVSLTNVSISDEGRYFCQLYTDPPQESYTTITVLV
PPRNLMIDIQKDTAVEGEEIEVNCTAMASKPATTIRWFKGNTELKGKSEVEEWSDMYTVTSQLMLKVHKED
DGVPVICQVEHPAVTGNLQTQRYLEVQYKPQVHIQMTYPLQGLTREGDALELTCEAIGKPQPVMVTWVRVD
DEMPQHAVLSGPNLFINNLNKTDNGTYRCEASNIVGKAHSDYMLYVYDPPTTIPPPTTTTTTTTTTTTTIL
TIITDSRAGEEGSIRAVDHAVIGGVVAVVVFAMLCLLIILGRYFARHKGTYFTHEAKGADDAADADTAIIN
AEGGQNNSEEKKE
```

CADM4 (human canonical, Uniprot ID Q8NFZ8, 388 AA, 42.8 kDa):

```
MGRARRFQWPLLLLWAAAAGPGAGQEVQTENVTVAEGGVAEITCRLHQYDGSIVVIQNPARQTLFFNGTRA
LKDERFQLEEFSPRRVRIRLSDARLEDEGGYFCQLYTEDTHHQIATLTVLVAPENPVVEVREQAVEGGEVE
LSCLVPRSRPAATLRWYRDRKELKGVSSSQENGKVWSVASTVRFRVDRKDDGGIIICEAQNQALPSGHSKQ
TQYVLDVQYSPTARIHASQAVVREGDTLVLTCAVTGNPRPNQIRWNRGNESLPERAEAVGETLTLPGLVSA
DNGTYTCEASNKHGHARALYVLVVYDPGAVVEAQTSVPYAIVGGILALLVFLIICVLVGMVWCSVRQKGSY
LTHEASGLDEQGEAREAFLNGSDGHKRKEEFFI
```

CD55 (human canonical, Uniprot ID P08174-1, 381 AA, MW 41.4 kDa):

```
MTVARPSVPAALPLLGELPRLLLLVLLCLPAVWGDCGLPPDVPNAQPALEGRTSFPEDTVITYKCEESFVK
IPGEKDSVICLKGSQWSDIEEFCNRSCEVPTRLNSASLKQPYITQNYFPVGTVVEYECRPGYRREPSLSPK
LTCLQNLKWSTAVEFCKKKSCPNPGEIRNGQIDVPGGILFGATISFSCNTGYKLFGSTSSFCLISGSSVQW
SDPLPECREIYCPAPPQIDNGIIQGERDHYGYRQSVTYACNKGFTMIGEHSIYCTVNNDEGEWSGPPPECR
GKSLTSKVPPTVQKPTTVNVPTTEVSPTSQKTTTKTTTPNAQATRSTPVSRTTKHFHETTPNKGSGTTSGT
TRLLSGHTCFTLTGLLGTLVTMGLLT
```

CD99L2 (human canonical, Uniprot ID Q8TCZ2-1, 262 AA, 28.0 kDa):

MVAWRSAFLVCLAFSLATLVQRGSGDFDDFNLEDAVKETSSVKQPWDHTTTTTTNRPGTTRAPAKPPGSGL
DLADALDDQDDGRRKPGIGGRERWNHVTTTTKRPVTTRAPANTLGNDFDLADALDDRNDRDDGRRKPIAGG
GGFSDKDLEDIVGGGEYKPDKGKGDGRYGSNDDPGSGMVAEPGTIAGVASALAMALIGAVSSYISYQQKKF
CFSIQQGLNADYVKGENLEAVVCEEPQVKYSTLHTQSAEPPPPPEPARI

CDH2 (human canonical, Uniprot ID P19022-1, 906 AA, MW 99.8 kDa):

MCRIAGALRTLLPLLAALLQASVEASGEIALCKTGFPEDVYSAVLSKDVHEGQPLLNVKFSNCNGKRKVQY
ESSEPADFKVDEDGMVYAVRSFPLSSEHAKFLIYAQDKETQEKWQVAVKLSLKPTLTEESVKESAEVEEIV
FPRQFSKHSGHLQRQKRDWVIPPINLPENSRGPFPQELVRIRSDRDKNLSLRYSVTGPGADQPPTGIFIIN
PISGQLSVTKPLDREQIARFHLRAHAVDINGNQVENPIDIVINVIDMNDNRPEFLHQVWNGTVPEGSKPGT
YVMTVTAIDADDPNALNGMLRYRIVSQAPSTPSPNMFTINNETGDIITVAAGLDREKVQQYTLIIQATDME
GNPTYGLSNTATAVITVTDVNDNPPEFTAMTFYGEVPENRVDIIVANLTVTDKDQPHTPAWNAVYRISGGD
PTGRFAIQTDPNSNDGLVTVVKPIDFETNRMFVLTVAAENQVPLAKGIQHPPQSTATVSVTVIDVNENPYF
APNPKIIRQEEGLHAGTMLTTFTAQDPDRYMQQNIRYTKLSDPANWLKIDPVNGQITTIAVLDRESPNVKN
NIYNATFLASDNGIPPMSGTGTLQIYLLDINDNAPQVLPQEAETCETPDPNSINITALDYDIDPNAGPFAF
DLPLSPVTIKRNWTITRLNGDFAQLNLKIKFLEAGIYEVPIIITDSGNPPKSNISILRVKVCQCDSNGDCT
DVDRIVGAGLGTGAIIAILLCIIILLILVLMFVVWMKRRDKERQAKQLLIDPEDDVRDNILKYDEEGGGEE
DQDYDLSQLQQPDTVEPDAIKPVGIRRMDERPIHAEPQYPVRSAAPHPGDIGDFINEGLKAADNDPTAPPY
DSLLVFDYEGSGSTAGSLSSLNSSSSGGEQDYDYLNDWGPRFKKLADMYGGGDD

CXADR (human canonical, Uniprot ID P78310-1, 365 AA, 40.0 kDa):

MALLLCFVLLCGVVDFARSLSITTPEEMIEKAKGETAYLPCKFTLSPEDQGPLDIEWLISPADNQKVDQVI
ILYSGDKIYDDYYPDLKGRVHFTSNDLKSGDASINVTNLQLSDIGTYQCKVKKAPGVANKKIHLVVLVKPS
GARCYVDGSEEIGSDFKIKCEPKEGSLPLQYEWQKLSDSQKMPTSWLAEMTSSVISVKNASSEYSGTYSCT
VRNRVGSDQCLLRLNVVPPSNKAGLIAGAIIGTLLALALIGLIIFCCRKKRREEKYEKEVHHDIREDVPPP
KSRTSTARSYIGSNHSSLGSMSPSNMEGYSKTQYNQVPSEDFERTPQSPTLPPAKVAAPNLSRMGAIPVMI
PAQSKDGSIV

DSG2 (mouse canonical, Uniprot ID O55111, 1122 AA, MW 122.4 kDa):

MARSPGDRCALLLLVQLLAVVCLDFGNGLHLEVFSPRNEGKPFPKHTHLVRQKRAWITAPVALREGEDLSR
KNPIAKIHSDLAEEKGIKITYKYTGKGITEPPFGIFVFDRNTGELNITSILDREETPYFLLTGYALDSRGN
NLEKPLELRIKVLDINDNEPVFTQEVFVGSIEELSAAHTLVMKITATDADDPETLNAKVSYRIVSQEPANS
HMFYLNKDTGEIYTTSFTLDREEHSSYSLTVEARDGNGQITDKPVQQAQVQIRILDVNDNIPVVENKMYEG
TVEENQVNVEVMRIKVTDADEVGSDNWLANFTFASGNEGGYFHIETDTQTNEGIVTLVKEVDYEEMKKLDL
SIIVTNKAAFHKSILSKYKATPIPITVKVKNVVEGIHFKSSVVSFRASEAMDRSSLSRSIGNFQVFDEDTG
QAAKVTYVKVQDTDNWVSVDSVTSEIKLVKIPDFESRYVQNGTYTAKVVAISKEHPQKTITGTIVITVEDV
NDNCPVLVDSVRSVCEDEPYVNVTAEDLDGAQNSAPFSFSIIDQPPGTAQKWKITHQESTSVLLQQSERKR
GRSEIPFLISDSQGFSCPERQVLQLTVCECLKGGGCVAAQYDNYVGLGPAAIALMILALLLLLLVPLLLLI
CHCGGGAKGFTPIPGTIEMLHPWNNEGAPPEDKVVPSLLVADHAESSAVRGGVGGAMLKEGMMKGSSSASV
TKGQHELSEVDGRWEEHRSLLTAGATHHVRTAGTIAANEAVRTRATGSSRDMSGARGAVAVNEEFLRSYFT
EKAASYNGEDDLHMAKDCLLVYSQEDTASLRGSVGCCSFIEGELDDLFLDDLGLKFKTLAEVCLGRKIDLD
VDIEQRQKPVREASVSAASGSHYEQAVTSSESAYSSNTGFPAPKPLHEVHTEKVTQEIVTESSVSSRQSQK
VVPPPDPVASGNIIVTETSYAKGSAVPPSTVLLAPRQPQSLIVTERVYAPTSTLVDQHYANEEKVLVTERV
IQPNGGIPKPLEVTQHLKDAQYVMVRERESILAPSSGVQPTLAMPSVAAGGQNVTVTERILTPASTLQSSY
QIPSETSITARNTVLSSVGSIGPLPNLDLEESDRPNSTITTSSTRVTKHSTMQHSYS

F11R (human canonical, Uniprot ID Q9Y624-1, 299 AA, MW 32.6 kDa):

MGTKAQVERKLLCLFILAILLCSLALGSVTVHSSEPEVRIPENNPVKLSCAYSGFSSPRVEWKFDQGDTTR
LVCYNNKITASYEDRVTFLPTGITFKSVTREDTGTYTCMVSEEGGNSYGEVKVKLIVLVPPSKPTVNIPSS
ATIGNRAVLTCSEQDGSPPSEYTWFKDGIVMPTNPKSTRAFSNSSYVLNPTTGELVFDPLSASDTGEYSCE
ARNGYGTPMTSNAVRMEAVERNVGVIVAAVLVTLILLGILVFGIWFAYSRGHFDRTKKGTSSKKVIYSQPS
ARSEGEFKQTSSFLV

GPC1 (human canonical*, Uniprot ID P35052-1, 558 AA, MW 61.7 kDa):

MELRARGWWLLCAAAALVACARGDPASKSRSCGEVRQIYGAKGFSLSDVPQAEISGEHLRICPQGYTCCTS
EMEENLANRSHAELETALRDSSRVLQAMLATQLRSFDDHFQHLLNDSERTLQATFPGAFGELYTQNARAFR
DLYSELRLYYRGANLHLEETLAEFWARLLERLFKQLHPQLLLPDDYLDCLGKQAEALRPFGEAPRELRLRA
TRAFVAARSFVQGLGVASDVVRKVAQVPLGPECSRAVMKLVYCAHCLGVPGARPCPDYCRNVLKGCLANQA
DLDAEWRNLLDSMVLITDKFWGTSGVESVIGSVHTWLAEAINALQDNRDTLTAKVIQGCGNPKVNPQGPGP
EEKRRRGKLAPRERPPSGTLEKLVSEAKAQLRDVQDFWISLPGTLCSEKMALSTASDDRCWNGMARGRYLP
EVMGDGLANQINNPEVEVDITKPDMTIRQQIMQLKIMTNRLRSAYNGNDVDFQDASDDGSGSGSGDGCLDD
LCGRKVSRKSSSSRTPLTHALPGLSEQEGQKTSAASCPQPPTFLLPLLLFLALTVARPRWR

*S500G natural variant

GPC2 (human canonical, Uniprot ID Q8N158, 579 AA, MW 62.8 kDa):

MSALRPLLLLLLPLCPGPGPGPGSEAKVTRSCAETRQVLGARGYSLNLIPPALISGEHLRVCPQEYTCCSS
ETEQRLIRETEATFRGLVEDSGSFLVHTLAARHRKFDEFFLEMLSVAQHSLTQLFSHSYGRLYAQHALIFN
GLFSRLRDFYGESGEGLDDTLADFWAQLLERVFPLLHPQYSFPPDYLLCLSRLASSTDGSLQPFGDSPRRL
RLQITRTLVAARAFVQGLETGRNVVSEALKVPVSEGCSQALMRLIGCPLCRGVPSLMPCQGFCLNVVRGCL
SSRGLEPDWGNYLDGLLILADKLQGPFSFELTAESIGVKISEGLMYLQENSAKVSAQVFQECGPPDPVPAR
NRRAPPPREEAGRLWSMVTEEERPTTAAGTNLHRLVWELRERLARMRGFWARLSLTVCGDSRMAADASLEA
APCWTGAGRGRYLPPVVGGSPAEQVNNPELKVDASGPDVPTRRRRLQLRAATARMKTAALGHDLDGQDADE
DASGSGGGQQYADDWMAGAVAPPARPPRPPYPPRRDGSGGKGGGGSARYNQGRSRSGGASIGFHTQTILIL
SLSALALLGPR

GPC3 (human canonical, Uniprot ID P51654-1, 580 AA, MW 65.6 kDa):

MAGTVRTACLVVAMLLSLDFPGQAQPPPPPPDATCHQVRSFFQRLQPGLKWVPETPVPGSDLQVCLPKGPT
CCSRKMEEKYQLTARLNMEQLLQSASMELKFLIIQNAAVFQEAFEIVVRHAKNYTNAMFKNNYPSLTPQAF
EFVGEFFTDVSLYILGSDINVDDMVNELFDSLFPVIYTQLMNPGLPDSALDINECLRGARRDLKVFGNFPK
LIMTQVSKSLQVTRIFLQALNLGIEVINTTDHLKFSKDCGRMLTRMWYCSYCQGLMMVKPCGGYCNVVMQG
CMAGVVEIDKYWREYILSLEELVNGMYRIYDMENVLLGLFSTIHDSIQYVQKNAGKLTTTIGKLCAHSQQR
QYRSAYYPEDLFIDKKVLKVAHVEHEETLSSRRRELIQKLKSFISFYSALPGYICSHSPVAENDTLCWNGQ
ELVERYSQKAARNGMKNQFNLHELKMKGPEPVVSQIIDKLKHINQLLRTMSMPKGRVLDKNLDEEGFESGD
CGDDEDECIGGSGDGMIKVKNQLRFLAELAYDLDVDDAPGNSQQATPKDNEISTFHNLGNVHSPLKLLTSM
AISVVCFFFLVH

GPC4 (human canonical, Uniprot ID O75487-1, 556 AA, MW 62.4 kDa):

MARFGLPALLCTLAVLSAALLAAELKSKSCSEVRRLYVSKGFNKNDAPLHEINGDHLKICPQGSTCCSQEM
EEKYSLQSKDDFKSVVSEQCNHLQAVFASRYKKFDEFFKELLENAEKSLNDMFVKTYGHLYMQNSELFKDL
FVELKRYYVVGNVNLEEMLNDFWARLLERMFRLVNSQYHFTDEYLECVSKYTEQLKPFGDVPRKLKLQVTR
AFVAARTFAQGLAVAGDVVSKVSVVNPTAQCTHALLKMIYCSHCRGLVTVKPCYNYCSNIMRGCLANQGDL
DFEWNNFIDAMLMVAERLEGPFNIESVMDPIDVKISDAIMNMQDNSVQVSQKVFQGCGPPKPLPAGRISRS
ISESAFSARFRPHHPEERPTTAAGTSLDRLVTDVKEKLKQAKKFWSSLPSNVCNDERMAAGNGNEDDCWNG

KGKSRYLFAVTGNGLANQGNNPEVQVDTSKPDILILRQIMALRVMTSKMKNAYNGNDVDFFDISDESSGEG
SGSGCEYQQCPSEFDYNATDHAGKSANEKADSAGVRPGAQAYLLTVFCILFLVMQREWR

GPC5 (human canonical, Uniprot ID P78333, 572 AA, MW 63.7 kDa):

MDAQTWPVGFRCLLLLALVGSARSEGVQTCEEVRKLFQWRLLGAVRGLPDSPRAGPDLQVCISKKPTCCTR
KMEERYQIAARQDMQQFLQTSSSTLKFLISRNAAAFQETLETLIKQAENYTSILFCSTYRNMALEAAASVQ
EFFTDVGLYLFGADVNPEEFVNRFFDSLFPLVYNHLINPGVTDSSLEYSECIRMARRDVSPFGNIPQRVMG
QMGRSLLPSRTFLQALNLGIEVINTTDYLHFSKECSRALLKMQYCPHCQGLALTKPCMGYCLNVMRGCLAH
MAELNPHWHAYIRSLEELSDAMHGTYDIGHVLLNFHLLVNDAVLQAHLNGQKLLEQVNRICGRPVRTPTQS
PRCSFDQSKEKHGMKTTTRNSEETLANRRKEFINSLRLYRSFYGGLADQLCANELAAADGLPCWNGEDIVK
SYTQRVVGNGIKAQSGNPEVKVKGIDPVINQIIDKLKHVVQLLQGRSPKPDKWELLQLGSGGGMVEQVSGD
CDDEDGCGGSGSGEVKRTLKITDWMPDDMNFSDVKQIHQTDTGSTLDTTGAGCAVATESMTFTLISVVMLL
PGIW

GPC6 (human canonical, Uniprot ID Q9Y625, 555 AA, MW 62.7 kDa):

MPSWIGAVILPLLGLLLSLPAGADVKARSCGEVRQAYGAKGFSLADIPYQEIAGEHLRICPQEYTCCTTEM
EDKLSQQSKLEFENLVEETSHFVRTTFVSRHKKFDEFFRELLENAEKSLNDMFVRTYGMLYMQNSEVFQDL
FTELKRYYTGGNVNLEEMLNDFWARLLERMFQLINPQYHFSEDYLECVSKYTDQLKPFGDVPRKLKIQVTR
AFIAARTFVQGLTVGREVANRVSKVSPTPGCIRALMKMLYCPYCRGLPTVRPCNNYCLNVMKGCLANQADL
DTEWNLFIDAMLLVAERLEGPFNIESVMDPIDVKISEAIMNMQENSMQVSAKVFQGCGQPKPAPALRSARS
APENFNTRFRPYNPEERPTTAAGTSLDRLVTDIKEKLKLSKKVWSALPYTICKDESVTAGTSNEEECWNGH
SKARYLPEIMNDGLTNQINNPEVDVDITRPDTFIRQQIMALRVMTNKLKNAYNGNDVNFQDTSDESSGSGS
GSGCMDDVCPTEFEFVTTEAPAVDPDRREVDSSAAQRGHSLLSWSLTCIVLALQRLCR

IGSF8 (human canonical, Uniprot ID Q969P0-1, 613 AA, MW 65.0 kDa):

MGALRPTLLPPSLPLLLLLMLGMGCWAREVLVPEGPLYRVAGTAVSISCNVTGYEGPAQQNFEWFLYRPEA
PDTALGIVSTKDTQFSYAVFKSRVVAGEVQVQRLQGDAVVLKIARLQAQDAGIYECHTPSTDTRYLGSYSG
KVELRVLPDVLQVSAAPPGPRGRQAPTSPPRMTVHEGQELALGCLARTSTQKHTHLAVSFGRSVPEAPVGR
STLQEVVGIRSDLAVEAGAPYAERLAAGELRLGKEGTDRYRMVVGGAQAGDAGTYHCTAAEWIQDPDGSWA
QIAEKRAVLAHVDVQTLSSQLAVTVGPGERRIGPGEPLELLCNVSGALPPAGRHAAYSVGWEMAPAGAPGP
GRLVAQLDTEGVGSLGPGYEGRHIAMEKVASRTYRLRLEAARPGDAGTYRCLAKAYVRGSGTRLREAASAR
SRPLPVHVREEGVVLEAVAWLAGGTVYRGETASLLCNISVRGGPPGLRLAASWWVERPEDGELSSVPAQLV
GGVGQDGVAELGVRPGGGPVSVELVGPRSHRLRLHSLGPEDEGVYHCAPSAWVQHADYSWYQAGSARSGPV
TVYPYMHALDTLFVPLLVGTGVALVTGATVLGTITCCFMKRLRKR

ITGA6 (human isoform Alpha-6X1A*, Uniprot ID P23229-2, 1073 AA, 119.5 kDa):

MAAAGQLCLLYLSAGLLSRLGAAFNLDTREDNVIRKYGDPGSLFGFSLAMHWQLQPEDKRLLLVGAPRAEA
LPLQRANRTGGLYSCDITARGPCTRIEFDNDADPTSESKEDQWMGVTVQSQGPGGKVVTCAHRYEKRQHVN
TKQESRDIFGRCYVLSQNLRIEDDMDGGDWSFCDGRLRGHEKFGSCQQGVAATFTKDFHYIVFGAPGTYNW
KGIVRVEQKNNTFFDMNIFEDGPYEVGGETEHDESLVPVPANSYLGFSLDSGKGIVSKDEITFVSGAPRAN
HSGAVVLLKRDMKSAHLLPEHIFDGEGLASSFGYDVAVVDLNKDGWQDIVIGAPQYFDRDGEVGGAVYVYM
NQQGRWNNVKPIRLNGTKDSMFGITVKNIGDINQDGYPDIAVGAPYDDLGKVFIYHGSANGINTKPTQVLK
GISPYFGYSIAGNMDLDRNSYPDVAVGSLSDSVTIFRSRPVINIQKTITVTPNRIDLRQKTACGAPSGICL
QVKSCFEYTANPAGYNPSISIVGTLEAEKERRKSGLSSRVQFRNQGSEPKYTQELTLKRQKQKVCMEETLW
LQDNIRDKLRPIPITASVEIQEPSSRRRVNSLPEVLPILNSDEPKTAHIDVHFLKEGCGDDNVCNSNLKLE
YKFCTREGNQDKFSYLPIQKGVPELVLKDQKDIALEITVTNSPSNPRNPTKDGDDAHEAKLIATFPDTLTY
SAYRELRAFPEKQLSCVANQNGSQADCELGNPFKRNSNVTFYLVLSTTEVTFDTPDLDINLKLETTSNQDN
LAPITAKAKVVIELLLSVSGVAKPSQVYFGGTVVGEQAMKSEDEVGSLIEYEFRVINLGKPLTNLGTATLN
IQWPKEISNGKWLLYLVKVESKGLEKVTCEPQKEINSLNLTESHNSRKKREITEKQIDDNRKFSLFAERKY
QTLNCSVNVNCVNIRCPLRGLDSKASLILRSRLWNSTFLEEYSKLNYDILMRAFIDVTAAAENIRLPNAG

TQVRVTVFPSKTVAQYSGVPWWIILVAILAGILMLALLVFILWKCGFFKRNKKDHYDATYHKAEIHAQPSD
KERLTSDA

*Most similar to Alpha-6X1A, differences compared to canonical: Δ255–293, A419T, 1084–1130 SRYDDSVPRYHAVRIRKEEREIKDEKYIDNLEKKQWITKWNENESYS→NKKDHYDATYHKAEIH AQPSDKERLTSDA

ITGB1 (human canonical, Uniprot ID P05556-1, 798 AA, 88.4 kDa):

MNLQPIFWIGLISSVCCVFAQTDENRCLKANAKSCGECIQAGPNCGWCTNSTFLQEGMPTSARCDDLEALK
KKGCPPDDIENPRGSKDIKKNKNVTNRSKGTAEKLKPEDITQIQPQQLVLRLRSGEPQTFTLKFKRAEDYP
IDLYYLMDLSYSMKDDLENVKSLGTDLMNEMRRITSDFRIGFGSFVEKTVMPYISTTPAKLRNPCTSEQNC
TSPFSYKNVLSLTNKGEVFNELVGKQRISGNLDSPEGGFDAIMQVAVCGSLIGWRNVTRLLVFSTDAGFHF
AGDGKLGGIVLPNDGQCHLENNMYTMSHYYDYPSIAHLVQKLSENNIQTIFAVTEEFQPVYKELKNLIPKS
AVGTLSANSSNVIQLIIDAYNSLSSEVILENGKLSEGVTISYKSYCKNGVNGTGENGRKCSNISIGDEVQF
EISITSNKCPKKDSDSFKIRPLGFTEEVEVILQYICECECQSEGIPESPKCHEGNGTFECGACRCNEGRVG
RHCECSTDEVNSEDMDAYCRKENSSEICSNNGECVCGQCVCRKRDNTNEIYSGKFCECDNFNCDRSNGLIC
GGNGVCKCRVCECNPNYTGSACDCSLDTSTCEASNGQICNGRGICECGVCKCTDPKFQGQTCEMCQTCLGV
CAEHKECVQCRAFNKGEKKDTCTQECSYFNITKVESRDKLPQPVQPDPVSHCKEKDVDDCWFYFTYSVNGN
NEVMVHVVENPECPTGPDIIPIVAGVVAGIVLIGLALLLIWKLLMIIHDRREFAKFEKEKMNAKWDTGENP
IYKSAVTTVVNPKYEGK

JAM3 (human canonical, Uniprot ID Q9BX67-1, 310 AA, MW 35.0 kDa):

MALRRPPRLRLCARLPDFFLLLLFRGCLIGAVNLKSSNRTPVVQEFESVELSCIITDSQTSDPRIEWKKIQ
DEQTTYVFFDNKIQGDLAGRAEILGKTSLKIWNVTRRDSALYRCEVVARNDRKEIDEIVIELTVQVKPVTP
VCRVPKAVPVGKMATLHCQESEGHPRPHYSWYRNDVPLPTDSRANPRFRNSSFHLNSETGTLVFTAVHKDD
SGQYYCIASNDAGSARCEEQEMEVYDLNIGGIIGGVLVVLAVLALITLGICCAYRRGYFINNKQDGESYKN
PGKPDGVNYIRTDEEGDFRHKSSFVI

KIRREL (human canonical, Uniprot ID Q96J84-1, 757 AA, 83.5 kDa):

MLSLLVWILTLSDTFSQGTQTRFSQEPADQTVVAGQRAVLPCVLLNYSGIVQWTKDGLALGMGQGLKAWPR
YRVVGSADAGQYNLEITDAELSDDASYECQATEAALRSRRAKLTVLIPPEDTRIDGGPVILLQAGTPHNLT
CRAFNAKPAATIIWFRDGTQQEGAVASTELLKDGKRETTVSQLLINPTDLDIGRVFTCRSMNEAIPSGKET
SIELDVHHPPTVTLSIEPQTVQEGERVVFTCQATANPEILGYRWAKGGFLIEDAHESRYETNVDYSFFTEP
VSCEVHNKVGSTNVSTLVNVHFAPRIVVDPKPTTTDIGSDVTLTCVWVGNPPLTLTWTKKDSNMVLSNSNQ
LLLKSVTQADAGTYTCRAIVPRIGVAEREVPLYVNGPPIISSEAVQYAVRGDGGKVECFIGSTPPPDRIAW
AWKENFLEVGTLERYTVERTNSGSGVLSTLTINNVMEADFQTHYNCTAWNSFGPGTAIIQLEEREVLPVGI
IAGATIGASILLIFFFIALVFFLYRRRKGSRKDVTLRKLDIKVETVNREPLTMHSDREDDTASVSTATRVM
KAIYSSFKDDVDLKQDLRCDTIDTREEYEMKDPTNGYYNVRAHEDRPSSRAVLYADYRAPGPARFDGRPSS
RLSHSSGYAQLNTYSRGPASDYGPEPTPPGPAAPAGTDTTSQLSYENYEKFNSHPFPGAAGYPTYRLGYPQ
APPSGLERTPYEAYDPIGKYATATRFSYTSQHSDYGQRFQQRMQTHV

LMAN2 (human canonical, Uniprot ID Q12907, 356 AA, MW 40.2 kDa):

MAAEGWIWRWGWGRRCLGRPGLLGPGPGPTTPLFLLLLLGSVTADITDGNSEHLKREHSLIKPYQGVGSSS
MPLWDFQGSTMLTSQYVRLTPDERSKEGSIWNHQPCFLKDWEMHVHFKVHGTGKKNLHGDGIALWYTRDRL
VPGPVFGSKDNFHGLAIFLDTYPNDETTERVFPYISVMVNNGSLSYDHSKDGRWTELAGCTADFRNRDHDT
FLAVRYSRGRLTVMTDLEDKNEWKNCIDITGVRLPTGYYFGASAGTGDLSDNHDIISMKLFQLMVEHTPDE
ESIDWTKIEPSVNFLKSPKDNVDDPTGNFRSGPLTGWRVFLLLLCALLGIVVCAVVGAVVFQKRQERNKRF
Y

LSR (human canonical, Uniprot ID Q86X29-1, 649 AA, MW 71.4 kDa):

MQQDGLGVGTRNGSGKGRSVHPSWPWCAPRPLRYFGRDARARRAQTAAMALLAGGLSRGLGSHPAAAGRDA
VVFVWLLLSTWCTAPARAIQVTVSNPYHVVILFQPVTLPCTYQMTSTPTQPIVIWKYKSFCRDRIADAFSP
ASVDNQLNAQLAAGNPGYNPYVECQDSVRTVRVVATKQGNAVTLGDYYQGRRITITGNADLTFDQTAWGDS
GVYYCSVVSAQDLQGNNEAYAELIVLGRTSGVAELLPGFQAGPIEDWLFVVVVCLAAFLIFLLLGICWCQC
CPHTCCCYVRCPCCPDKCCCPEALYAAGKAATSGVPSIYAPSTYAHLSPAKTPPPPAMIPMGPAYNGYPGG
YPGDVDRSSSAGGQGSYVPLLRDTDSSVASEVRSGYRIQASQQDDSMRVLYYMEKELANFDPSRPGPPSGR
VERAMSEVTSLHEDDWRSRPSRGPALTPIRDEEWGGHSPRSPRGWDQEPAREQAGGGWRARRPRARSVDAL
DDLTPPSTAESGSRSPTSNGGRSRAYMPPRSRSRDDLYDQDDSRDFPRSRDPHYDDFRSRERPPADPRSHH
HRTRDPRDNGSRSGDLPYDGRLLEEAVRKKGSEERRRPHKEEEEEAYYPPAPPPYSETDSQASRERRLKKN
LALSRESLVV

MCAM (human canonical, Uniprot ID P43121-1, 646 AA, MW 71.6 kDa):

MGLPRLVCAFLLAACCCCPRVAGVPGEAEQPAPELVEVEVGSTALLKCGLSQSQGNLSHVDWFSVHKEKRT
LIFRVRQGQGQSEPGEYEQRLSLQDRGATLALTQVTPQDERIFLCQGKRPRSQEYRIQLRVYKAPEEPNIQ
VNPLGIPVNSKEPEEVATCVGRNGYPIPQVIWYKNGRPLKEEKNRVHIQSSQTVESSGLYTLQSILKAQLV
KEDKDAQFYCELNYRLPSGNHMKESREVTVPVFYPTEKVWLEVEPVGMLKEGDRVEIRCLADGNPPPHFSI
SKQNPSTREAEEETTNDNGVLVLEPARKEHSGRYECQGLDLDTMISLLSEPQELLVNYVSDVRVSPAAPER
QEGSSLTLTCEAESSQDLEFQWLREETGQVLERGPVLQLHDLKREAGGGYRCVASVPSIPGLNRTQLVNVA
IFGPPWMAFKERKVWVKENMVLNLSCEASGHPRPTISWNVNGTASEQDQDPQRVLSTLNVLVTPELLETGV
ECTASNDLGKNTSILFLELVNLTTLTPDSNTTTGLSTSTASPHTRANSTSTERKLPEPESRGVVIVAVIVC
ILVLAVLGAVLYFLYKKGKLPCRRSGKQEITLPPSRKSELVVEVKSDKLPEEMGLLQGSSGDKRAPGDQGE
KYIDLRH

MDFI (human canonical, Uniprot ID Q99750, 246 AA, MW 25.0 kDa):

MYQVSGQRPSGCDAPYGAPSAAPGPAQTLSLLPGLEVVTGSTHPAEAAPEEGSLEEAATPMPQGNGPGIPQ
GLDSTDLDVPTEAVTCQPQGNPLGCTPLLPNDSGHPSELGGTRRAGNGALGGPKAHRKLQTHPSLASQGSK
KSKSSSKSTTSQIPLQAQEDCCVHCILSCLFCEFLTLCNIVLDCATCGSCSSEDSCLCCCCCGSGECADCD
LPCDLDCGILDACCESADCLEICMECCGLCFSS

NCAM1 (human canonical*, Uniprot ID P13591-2, 858 AA, MW 94.6 kDa):

MLQTKDLIWTLFFLGTAVSLQVDIVPSQGEISVGESKFFLCQVAGDAKDKDISWFSPNGEKLTPNQQRISV
VWNDDSSSTLTIYNANIDDAGIYKCVVTGEDGSESEATVNVKIFQKLMFKNAPTPQEFREGEDAVIVCDVV
SSLPPTIIWKHKGRDVILKKDVRFIVLSNNYLQIRGIKKTDEGTYRCEGRILARGEINFKDIQVIVNVPPT
IQARQNIVNATANLGQSVTLVCDAEGFPEPTMSWTKDGEQIEQEEDDEKYIFSDDSSQLTIKKVDKNDEAE
YICIAENKAGEQDATIHLKVFAKPKITYVENQTAMELEEQVTLTCEASGDPIPSITWRTSTRNISSEEKAS
WTRPEKQETLDGHMVVRSHARVSSLTLKSIQYTDAGEYICTASNTIGQDSQSMYLEVQYAPKLQGPVAVYT
WEGNQVNITCEVFAYPSATISWFRDGQLLPSSNYSNIKIYNTPSASYLEVTPDSENDFGNYNCTAVNRIGQ
ESLEFILVQADTPSSPSIDQVEPYSSTAQVQFDEPEATGGVPILKYKAEWRAVGEEVWHSKWYDAKEASME
GIVTIVGLKPETTYAVRLAALNGKGLGEISAASEFKTQPVQGEPSAPKLEGQMGEDGNSIKVNLIKQDDGG
SPIRHYLVRYRALSSEWKPEIRLPSGSDHVMLKSLDWNAEYEVYVVAENQQGKSKAAHFVFRTSAQPTAIP
ANGSPTSGLSTGAIVGILIVIFVLLLVVVDITCYFLNKCGLFMCIAVNLCGKAGPGAKGKDMEEGKAAFSK
DESKEPIVEVRTEEERTPNHDGGKHTEPNETTPLTEPEKGPVEAKPECQETETKPAPAEVKTVPNDATQTK
ENENKA

*S856N point mutation relative to canonical sequence

NCSTN (human isoform 2*, Uniprot ID Q92542-2, 689 AA, MW 76.7 kDa):

MDFNLILESLCRGNSVERKIYIPLNKTAPCVRLLNATHQIGCQSSISGDTGVIHVVEKEEDLQWVLTDGPN
PPYMVLLESKHFTRDLMEKLKGRTSRIAGLAVSLTKPSPASGFSPSVQCPNDGFGVYSNSYGPEFAHCREI
QWNSLGNGLAYEDFSFPIFLLEDENETKVIKQCYQDHNLSQNGSAPTFPLCAMQLFSHMHAVISTATCMRR
SSIQSTFSINPEIVCDPLSDYNVWSMLKPINTTGTLKPDDRVVVAATRLDSRSFFWNVAPGAESAVASFVT
QLAAAEALQKAPDVTTLPRNVMFVFFQGETFDYIGSSRMVYDMEKGKFPVQLENVDSFVELGQVALRTSLE
LWMHTDPVSQKNESVRNQVEDLLATLEKSGAGVPAVILRRPNQSQPLPPSSLQRFLRARNISGVVLADHSG
AFHNKYYQSIYDTAENINVSYPEWLSPEEDLNFVTDTAKALADVATVLGRALYELAGGTNFSDTVQADPQT
VTRLLYGFLIKANNSWFQSILRQDLRSYLGDGPLQHYIAVSSPTNTTYVVQYALANLTGTVVNLTREQCQD
PSKVPSENKDLYEYSWVQGPLHSNETDRLPRCVRSTARLARALSPAFELSQWSSTEYSTWTESRWKDIHAR
IFLIASKELELITLTVGFGILIFSLIVTYCINAKADVLFIAPREPGAVSY

*contains R637H point mutation relative to isoform 2

NECTIN2 (PVRL2 human isoform Alpha*, Uniprot ID Q92692-2, 479 AA, MW 51.4 kDa):

MARAAALLPSRSPPTPLLWPLLLLLLLETGAQDVRVQVLPEVRGQLGGTVELPCHLLPPVPGLYISLVTWQ
RPDAPANHQNVAAFHPKMGPSFPSPKPGSERLSFVSAKQSTGQDTEAELQDATLALHGLTVEDEGNYTCEF
ATFPKGSVRGMTWLRVIAKPKNQAEAQKVTFSQDPTTVALCISKEGRPPARISWLSSLDWEAKETQVSGTL
AGTVTVTSRFTLVPSGRADGVTVTCKVEHESFEEPALIPVTLSVRYPPEVSISGYDDNWYLGRTDATLSCD
VRSNPEPTGYDWSTTSGTFPTSAVAQGSQLVIHAVDSLFNTTFVCTVTNAVGMGRAEQVIFVRETPRASPR
DVGPLVWGAVGGTLLVLLLLAGGSLAFILLRVRRRRKSPGGAGGGASGDGGFYDPKAQVLGNGDPVFWTPV
VPGPMEPDGKDEEEEEEEEKAEKGLMLPPPPALEDDMESQLDGSLISRRAVYV

*This isoform is derived from GenBank sequence AF058448 as characterized by Warner, et al.(15) and contains an alternative transmembrane domain sequence relative the canonically defined isoform, Uniprot ID Q92692-1

NPTN (human isoform 1, Uniprot ID Q9Y639-1, 282 AA, MW 31.3 kDa):

MSGSSLPSALALSLLLVSGSLLPGPGAAQNEPRIVTSEEVIIRDSPVLPVTLQCNLTSSSHTLTYSYWTKN
GVELSATRKNASNMEYRINKPRAEDSGEYHCVYHFVSAPKANATIEVKAAPDITGHKRSENKNEGQDATMY
CKSVGYPHPDWIWRKKENGMPMDIVNTSGRFFIINKENYTELNIVNLQITEDPGEYECNATNAIGSASVVT
VLRVRSHLAPLWPFLGILAEIIILVVIIVVYEKRKRPDEVPDDDEPAGPMKTNSTNNHKDKNLRQRNTN

OCLN (human canonical*, Uniprot ID Q16625-1, 522 AA, MW 59.1 kDa):

MSSRPLESPPPYRPDEFKPNHYAPSNDIYGGEMHVRPMLSQPAYSFYPEDEILHFYKWTSPPGVIRILSML
IIVMCIAIFACVASTLAWDRGYGTSLLGGSVGYPYGGSGFGSYGSGYGYGYGYGYGYGGYTDPRAAKGFML
AMAAFCFIAALVIFVTSVIRSEMSRTRRYYLSVIIVSAILGIMVFIATIVYIMGVNPTAQSSGSLYGSQIY
ALCNQFYTPAATGLYVDQYSYHYCVVDPQEAIAIVLGFMIIVAFALIIFFAVKTRRKMDRYDKSNILWDKE
HIYDEQPPNVEEWVKNVSAGTQDVPSPPSDYVERVDSPMAYSSNGKVNDKRFYPESSYKSTPVPEVVQELP
LTSPVDDFRQPRYSSGGNFETPSKRAPAKGRAGRSKRTEQDHYETDYTTGGESCDELEEDWIREYPPITSD
QQRQLYKRNFDTGLQEYKSLQSELDEINKELSRLDKELDDYREESEEYMAAADEYNRLKQVKGSADYKSKK
NHCKQLKSKLSHIKKMVGDYDRQKT

*L233S point mutation relative to canonical sequence

PODXL (human isoform 2, Uniprot ID O00592-2, 526 AA, MW 55.4 kDa):

MRCALALSALLLLLLSTPPLLPSSPSPSPSPSQNATQTTTDSSNKTAPTPASSVTIMATDTAQQSTVPTSKA
NEILASVKATTLGVSSDSPGTTTLAQQVSGPVNTTVARGGGSGNPTTTIESPKSTKSADTTTVATSTATAK

PNTTSSQNGAEDTTNSGGKSSHSVTTDLTSTKAEHLTTPHPTSPLSPRQPTSTHPVATPTSSGHDHLMKIS
SSSSTVAIPGYTFTSPGMTTTLPSSVISQRTQQTSSQMPASSTAPSSQETVQPTSPATALRTPTLPETMSS
SPTAASTTHRYPKTPSPTVAHESNWAKCEDLETQTQSEKQLVLNLTGNTLCAGGASDEKLISLICRAVKAT
FNPAQDKCGIRLASVPGSQTVVVKEITIHTKLPAKDVYERLKDKWDELKEAGVSDMKLGDQGPPEEAEDRF
SMPLIITIVCMASFLLLVAALYGCCHQRLSQRKDQQRLTEELQTVENGYHDNPTLEVMETSSEMQEKKVVS
LNGELGDSWIVPLDNLTKDDLDEEEDTHL

PTK7 (human canonical*, Uniprot ID Q13308-1, 1070 AA, MW 118.4 kDa):

MGAARGSPARPRRLPLLSVLLLPLLGGTQTAIVFIKQPSSQDALQGRRALLRCEVEAPGPVHVYWLLDGAP
VQDTERRFAQGSSLSFAAVDRPQDSGTFQCVARDDVTGEEARSANASFNIKWIEAGPVVLKHPASEAEIQP
QTQVTLRCHIDGHPRPTYQWFRDGTPLSDGQSNHTVSSKERNLTLRPAGPEHSGLYSCCAHSAFGQACSSQ
NFTLSIADESFARVVLAPQDVVVARYEEAMFHCQFSAQPPPSLQWLFEDETPITNRSRPPHLRRATVFANG
SLLLTQVRPRNAGIYRCIGQGQRGPPIILEATLHLAEIEDMPLFEPRVFTAGSEERVTCLPPKGLPEPSVW
WEHAGVRLPTHGRVYQKGHELVLANIAESDAGVYTCHAANLAGQRRQDVNITVATVPSWLKKPQDSQLEEG
KPGYLDCLTQATPKPTVVWYRNQMLISEDSRFEVFKNGTLRINSVEVYDGTWYRCMSSTPAGSIEAQARVQ
VLEKLKFTPPPQPQQCMEFDKEATVPCSATGREKPTIKWERADGSSLPEWVTDNAGTLHFARVTRDDAGNY
TCIASNGPQGQIRAHVQLTVAVFITFKVEPERTTVYQGHTALLQCEAQGDPKPLIQWKGKDRILDPTKLGP
RMHIFQNGSLVIHDVAPEDSGRYTCIAGNSCNIKHTEAPLYVVDKPVPEESEGPGSPPPYKMIQTIGLSVG
AAVAYIIAVLGLMFYCKKRCKAKRLQKQPEGEEPEMECLNGGPLQNGQPSAEIQEEVALTSLGSGPAATNK
RHSTSDKMHFPRSSLQPITTLGKSEFGEVFLAKAQGLEEGVAETLVLVKSLQSKDEQQQLDFRRELEMFGK
LNHANVVRLLGLCREAEPHYMVLEYVDLGDLKQFLRISKSKDEKLKSQPLSTKQKVALCTQVALGMEHLSN
NRFVHKDLAARNCLVSAQRQVKVSALGLSKDVYNSEYYHFRQAWVPLRWMSPEAILEGDFSTKSDVWAFGV
LMWEVFTHGEMPHGGQADDEVLADLQAGKARLPQPEGCPSKLYRLMQRCWALSPKDRPSFSEIASALGDST
VDSKP

*L93P point mutation relative to canonical sequence

PTPRF (human isoform 2*, Uniprot ID P10586-2, 1898 AA, MW 211.7 kDa):

MAPEPAPGRTMVPLVPALVMLGLVAGAHGDSKPVFIKVPEDQTGLSGGVASFVCQATGEPKPRITWMKKGK
KVSSQRFEVIEFDDGAGSVLRIQPLRVQRDEAIYECTATNSLGEINTSAKLSVLEEEQLPPGFPSIDMGPQ
LKVVEKARTATMLCAAGGNPDPEISWFKDFLPVDPATSNGRIKQLRSGALQIESSEESDQGKYECVATNSA
GTRYSAPANLYVRVRRVAPRFSIPPSSQEVMPGGSVNLTCVAVGAPMPYVKWMMGAEELTKEDEMPVGRNV
LELSNVVRSANYTCVAISSLGMIEATAQVTVKALPKPPIDLVVTETTATSVTLTWDSGNSEPVTYYGIQYR
AAGTEGPFQEVDGVATTRYSIGGLSPFSEYAFRVLAVNSIGRGPPSEAVRARTGEQAPSSPPRRVQARMLS
ASTMLVQWEPPEEPNGLVRGYRVYYTPDSRRPPNAWHKHNTDAGLLTTVGSLLPGITYSLRVLAFTAVGDG
PPSPTIQVKTQQGVPAQPADFQAEVESDTRIQLSWLLPPQERIIMYELVYWAAEDEDQQHKVTFDPTSSYT
LEDLKPDTLYRFQLAARSDMGVGVFTPTIEARTAQSTPSAPPQKVMCVSMGSTTVRVSWVPPPADSRNGVI
TQYSVAYEAVDGEDRGRHVVDGISREHSSWDLVGLEKWTEYRVWVRAHTDVGPGPESSPVLVRTDEDVPSG
PPRKVEVEPLNSTAVHVYWKLPVPSKQHGQIRGYQVTYVRLENGEPRGLPIIQDVMLAEAQETTISGLTPE
TTYSVTVAAYTTKGDGARSKPKIVTTTGAVPGRPTMMISTTAMNTALLQWHPPKELPGELLGYRLQYCRAD
EARPNTIDFGKDDQHFTVTGLHKGTTYIFRLAAKNRAGLGEEFEKEIRTPEDLPSGFPQNLHVTGLTTSTT
ELAWDPPVLAERNGRIISYTVVFRDINSQQELQNITTDTRFTLTGLKPDTTYDIKVRAWTSKGSGPLSPSI
QSRTMPVEQVFAKNFRVAAAMKTSVLLSWEVPDSYKSAVPFKILYNGQSVEVDGHSMRKLIADLQPNTEYS
FVLMNRGSSAGGLQHLVSIRTAPDLLPHKPLPASAYIEDGRFDLSMPHVQDPSLVRWFYIVVVPIDRVGGS
MLTPRWSTPEELELDELLEAIEQGGEEQRRRRRQAERLKPYVAAQLDVLPETFTLGDKKNYRGFYNRPLSP
DLSYQCFVLASLKEPMDQKRYASSPYSDEIVVQVTPAQQQEEPEMLWVTGPVLAVILIIILIVIAILLFKRK
RTHSPSSKDEQSIGLKDSLLAHSSDPVEMRRLNYQTPGMRDHPPIPITDLADNIERLKANDGLKFSQEYES
IDPGQQFTWENSNLEVNKPKNRYANVIAYDHSRVILTSIDGVPGSDYINANYIDGYRKQNAYTATQGPLPE
TMGDFWRMVWEQRTATVVMMTRLEEKSRVKCDQYWPARGTETCGLIQVTLLDTVELATYTVRTFALHKSGS
SEKRELRQFQFMAWPDHGVPEYPTPILAFLRRVKACNPLDAGPMVVHCSAGVGRTGCFIVIDAMLERMKHE
KTVDIYGHVTCMRSQRNYMVQTEDQYVFIHEALLEAATCGHTEVPARNLYAHIQKLGQVPPGESVTAMELE
FKLLASSKAHTSRFISANLPCNKFKNRLVNIMPYELTRVCLQPIRGVEGSDYINASFLDGYRQQKAYIATQ
GPLAESTEDFWRMLWEHNSTIIVMLTKLREMGREKCHQYWPAERSARYQYFVVDPMAEYNMPQYILREFKV

TDARDGQSRTIRQFQFTDWPEQGVPKTGEGFIDFIGQVHKTKEQFGQDGPITVHCSAGVGRTGVFITLSIV
LERMRYEGVVDMFQTVKTLRTQRPAMVQTEDQYQLCYRAALEYLGSFDHYAT

*I1412T point mutation relative to isoform 2 sequence; Δ772–780 relative to canonical sequence

PVR (human canonical*, Uniprot ID P15151-1, 417 AA, MW 45.3 kDa):

MARAMAAAWPLLLVALLVLSWPPPGTGDVVVQAPTQVPGFLGDSVTLPCYLQVPNMEVTHVSQLTWARHGE
SGSMAVFHQTQGPSYSESKRLEFVAARLGAELRNASLRMFGLRVEDEGNYTCLFVTFPQGSRSVDIWLRVL
AKPQNTAEVQKVQLTGEPVPMARCVSTGGRPPAQITWHSDLGGMPNTSQVPGFLSGTVTVTSLWILVPSSQ
VDGKNVTCKVEHESFEKPQLLTVNLTVYYPPEVSISGYDNNWYLGQNEATLTCDARSNPEPTGYNWSTTMG
PLPPFAVAQGAQLLIRPVDKPINTTLICNVTNALGARQAELTVQVKEGPPSEHSGMSRNAIIFLVLGILVF
LILLGIGIYFYWSKCSREVLWHCHLCPSSTEHASASANGHVSYSAVSRENSSSQDPQTEGTR

*contains I340M point mutation relative to canonical (natural variant UniProtKB/Swiss-Prot: VAR_011736)

RTN4R (human canonical, Uniprot ID Q9BZR6, 473 AA, MW 50.7 kDa):

MKRASAGGSRLLAWVLWLQAWQVAAPCPGACVCYNEPKVTTSCPQQGLQAVPVGIPAASQRIFLHGNRISH
VPAASFRACRNLTILWLHSNVLARIDAAAFTGLALLEQLDLSDNAQLRSVDPATFHGLGRLHTLHLDRCGL
QELGPGLFRGLAALQYLYLQDNALQALPDDTFRDLGNLTHLFLHGNRISSVPERAFRGLHSLDRLLLHQNR
VAHVHPHAFRDLGRLMTLYLFANNLSALPTEALAPLRALQYLRLNDNPWVCDCRARPLWAWLQKFRGSSSE
VPCSLPQRLAGRDLKRLAANDLQGCAVATGPYHPIWTGRATDEEPLGLPKCCQPDAADKASVLEPGRPASA
GNALKGRVPPGDSPPGNGSGPRHINDSPFGTLPGSAEPPLTAVRPEGSEPPGFPTSGPRRRPGCSRKNRTR
SHCRLGQAGSGGGGTGDSEGSGALPSLTCSLTPLGLALVLWTVLGPC

SMPDL3B (human isoform 2, Uniprot ID Q92485-2, 373 AA, 41.7 kDa):

MRLLAWLIFLANWGGARAEPGKFWHIADLHLDPDYKVSKDPFQVCPSAGSQPVPDAGPWGDYLCDSPWALI
NSSIYAMKEIEPEPDFILWTGDDTPHVPDEKLGEAAVLEIVERLTKLIREVFPDTKVYAALGNHDFHPKNQ
FPAGSNNIYNQIAELWKPWLSNESIALFKKGAFYCEKLPGPSGAGRIVVLNTNLYYTSNALTADMADPGQQ
FQWLEDVLTDASKAGDMVYIVGHVPPGFFEKTQNKAWFREGFNEKYLKVVRKHHRVIAGQFFGHHHTDSFR
MLYDDAGVPISAMFITPGVTPWKTTLPGVVNGANNPAIRVFEYDRATLSLKVRSPAEARGGGWEGLKCITT
FPHSQLIHLPLTTEPQEG

TMEM30A (human isoform 2, Uniprot ID Q9NV96-2, 325 AA, MW 36.7 kDa):

MAMNYNAKDEVDGGPPCAPGGTAKTRRPDNTAFKQQRLPAWQPILTAGTVLPIFFIIGLIFIPIGIGIFVT
SNNIREIEGNVFMYYGLSNFYQNHRRYVKSRDDSQLNGDSSALLNPSKECEPYRRNEDKPIAPCGAIANSM
FNDTLELFLIGNDSYPIPIALKKKGIAWWTDKNVKFRNPPGGDNLEERFKGTTKPVNWLKPVYMLDSDPDN
NGFINEDFIVWMRTAALPTFRKLYRLIERKSDLHPTLPAGRYSLNVTYNYPVHYFDGRKRMILSTISWMGG
KNPFLGIAYIAVGSISFLLGVVLLVINHKYRNSSNTADITI

Appendix III: Calcium imaging screen representative data for each candidate gene, alphabetically, showing the Yoda1 response with two representative trials on the left and right. Cells without visible transfection (UT, black traces) are being compared to those with visible red fluorescence (red traces) in the same field of view, together with an overlay of the average ± SD, as described in the Materials and Methods. Genes are C-terminally fused to mCherry unless otherwise noted (tdT = tdTomato). See Appendix II for cDNA and protein sequences that were used. If applicable (e.g., if multiple constructs for the same gene were tested), the corresponding UniProt ID is noted. Cells were excluded from analysis if they were obviously unhealthy (round morphology or baseline calcium ratio >1.5), did not respond to Yoda1 within the first 10 s of application and/or did not show green PIEZO1-GFP fluorescence, or did not respond to ionomycin following the Yoda1 stimulus. In a few cases, very few transfected cells remained in the field of view, either due to poor transfection efficiency, expression, or cell health issues resulting from transient overexpression. This has been noted in Appendix II.

Appendix III, continued:

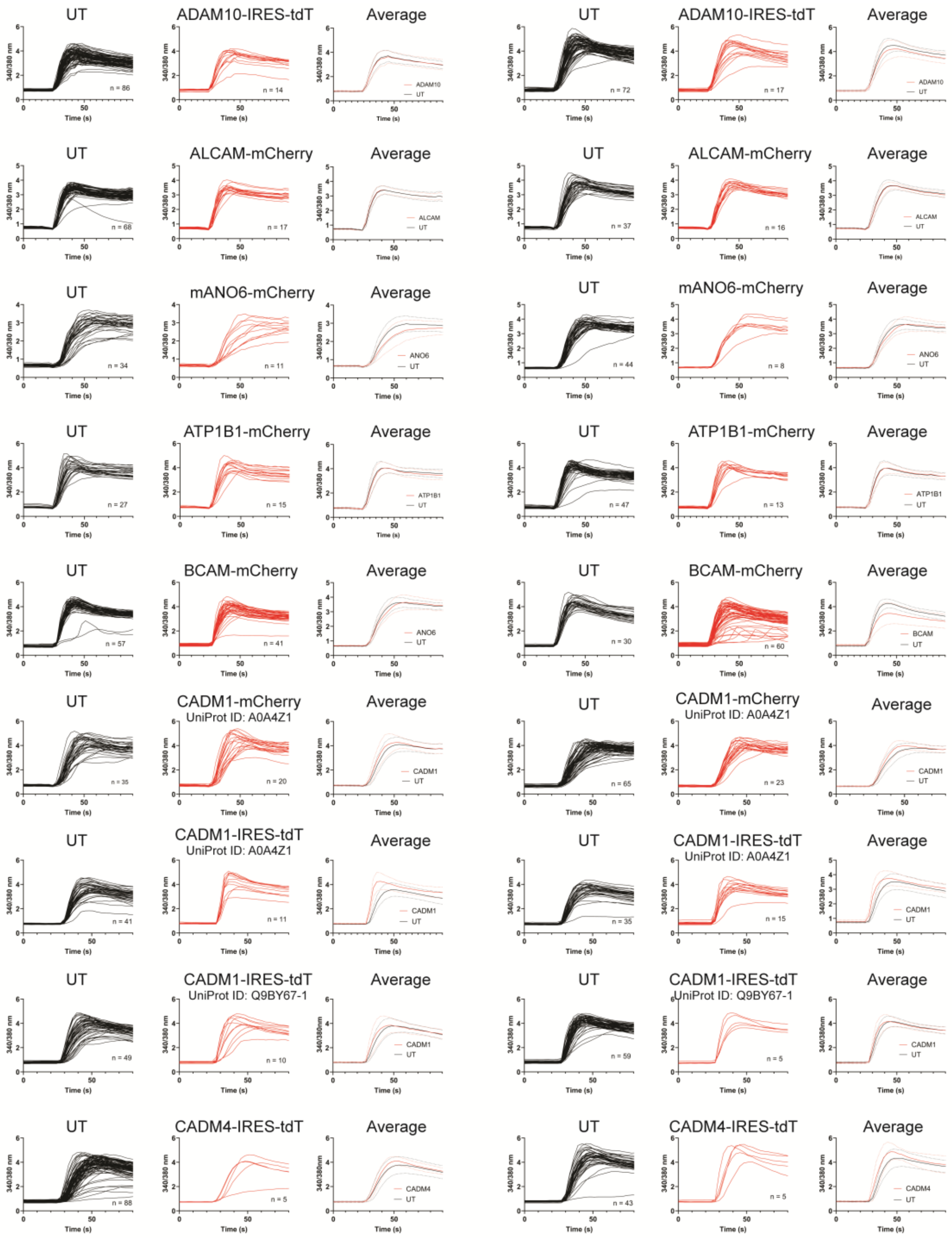

Appendix III, continued:

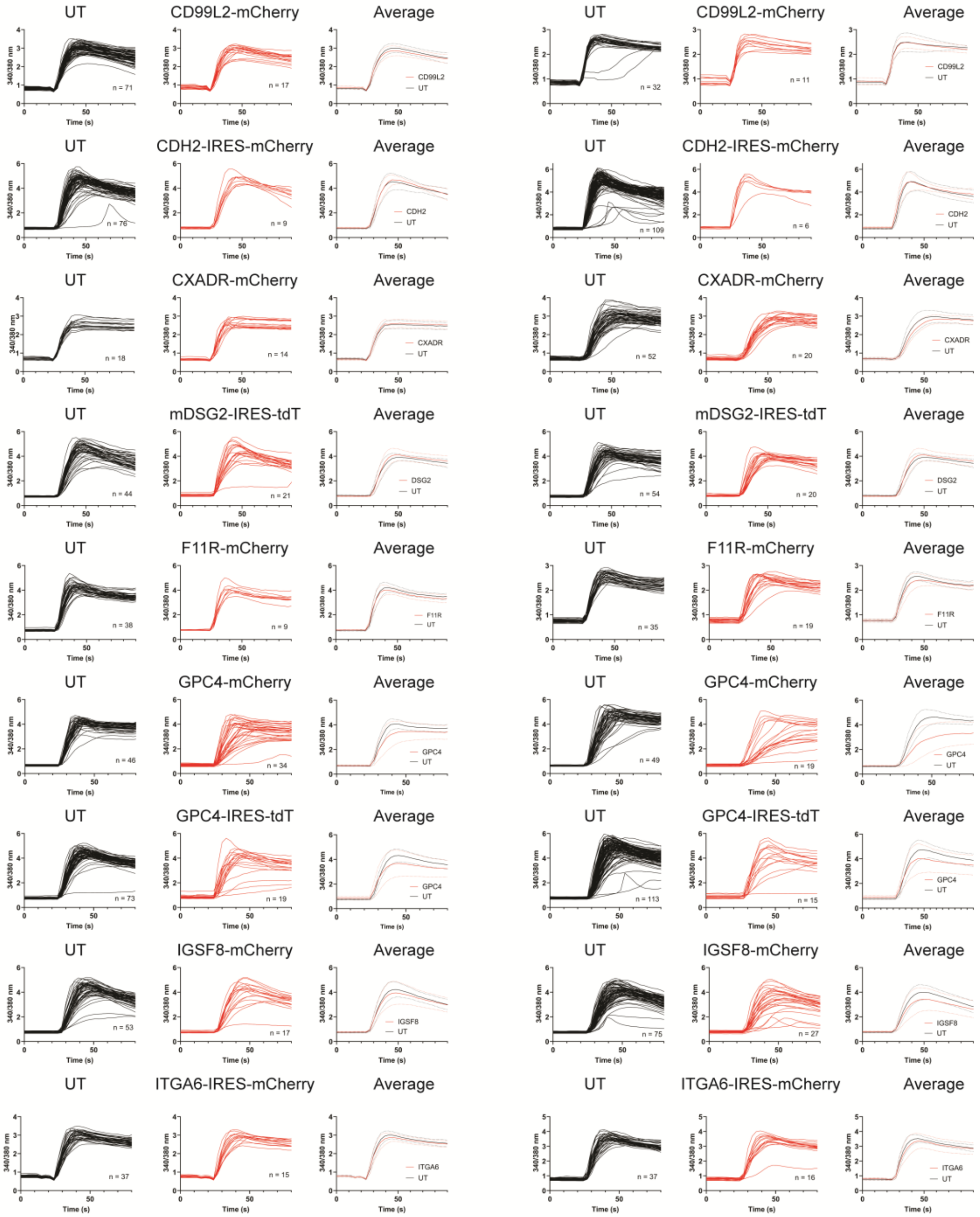

Appendix III, continued:

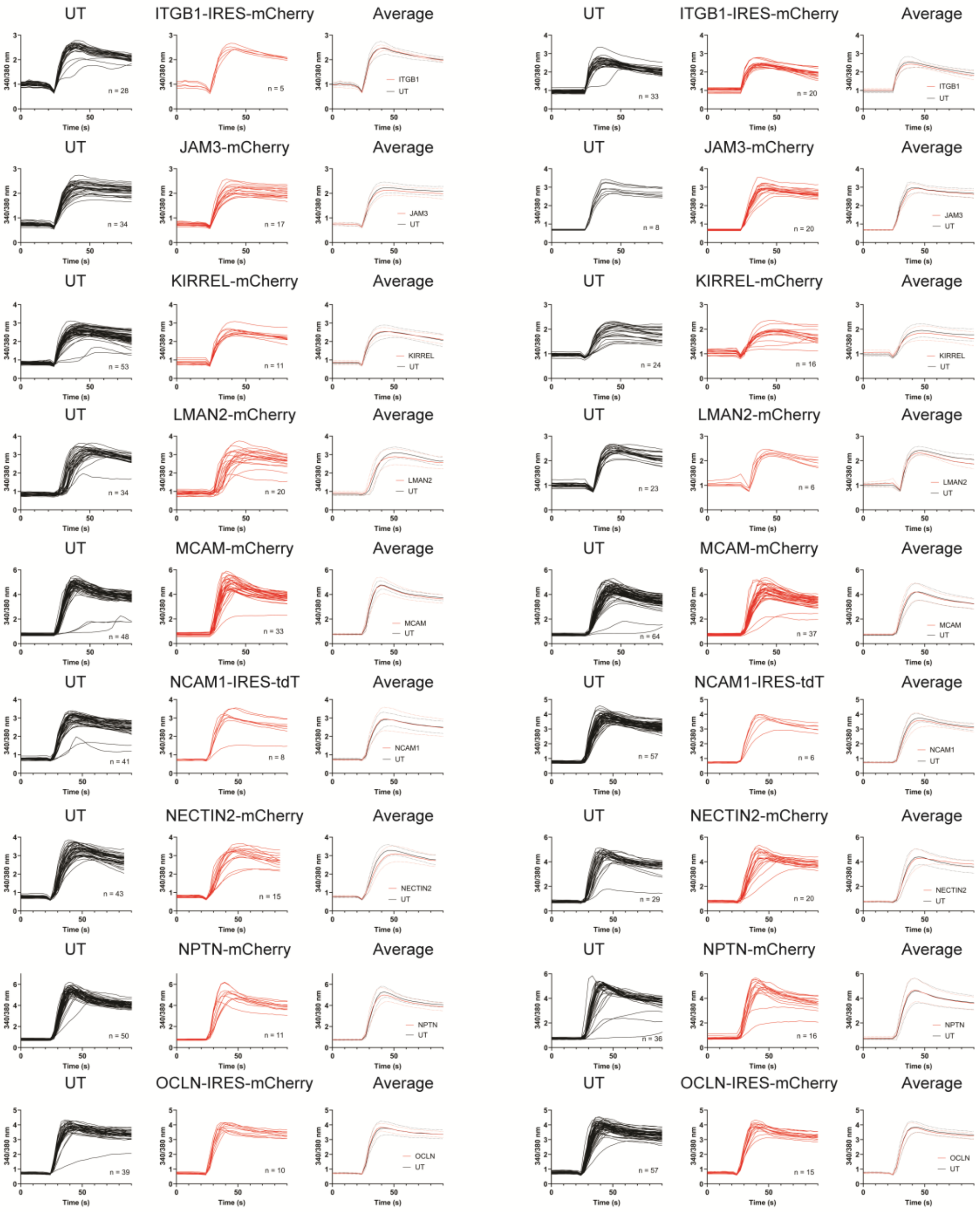

Appendix III, continued:

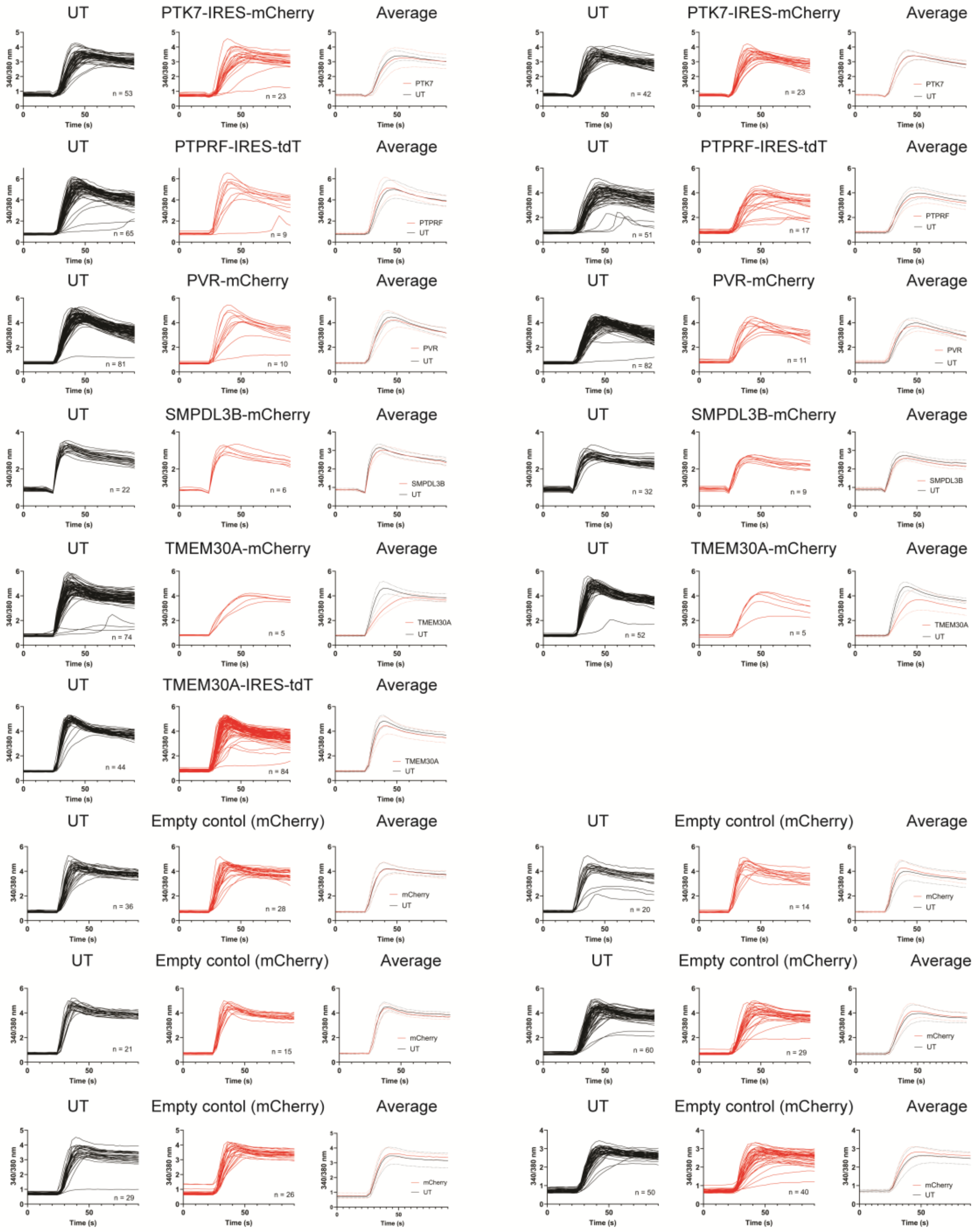

Supplementary File 2 (separate file). PIEZO1 interactome in the HEK293F PIEZO1 stable cell line, showing all proteins identified in a single multiplexed TMT proximity labeling dataset prior to any filtering ('PIEZO1 interactome, HEK stable' tab). The final 185 high-confidence proteins shown in Fig. 1 are listed in the 'Final 185 proteins' tab (3 separately treated biological replicates for each condition).

How to read this spreadsheet:

TMT1–3 are 3 biological replicates performed with a DMSO vehicle control, while TMT4–6 are 3 biological replicates performed in the presence of 10 μM Yoda1 agonist (~1–2-min. application). Normalized signal from each TMT channel is indicated for each protein in columns F-K (Sum of TMT1–6 = 100%). Column M is the ratio of average signal coming from TMT4–6 (Yoda1)/ the average from TMT1–3 (DMSO control) and is illustrated in Fig. S1, E.

Color coding indicates which proteins were omitted with each filter and why:

- Yellow highlight (Columns A–M): Eliminated because spectral count (Column D) was <5 (our cutoff for reliable protein ID)

- Red text (Columns A–M): Eliminated because the protein was not captured in all four TMT datasets in HEK cells (3 with transient overexpression, 1 with the stable cell line, representing 9 total biological replicates). If a protein was not seen in all 4 independent TMT experiments, this is indicated by a 'FALSE' in column 'L'). Columns Y–AA indicate whether a protein was captured ('TRUE') or not ('FALSE') in each of three transient PIEZO1 overexpression TMT datasets in HEK cells in addition to the stable cell line.

- Blue text (Columns A–M): Eliminated because the protein was equally enriched by PIEZO1 lacking a BBS tag (these are primarily endogenous biotin-associated proteins that were captured during pull-down with streptavidin)

- Red text (Columns R–V): Eliminated because the protein is present in >25% (179/716) of CRAPome datasets. Column T indicates this number out of 716. Column W indicates which of the CRAPome filtered proteins are annotated to be intracellular or cytoplasmic (Y = YES, N = NO) based on GO analysis (https://geneontology.org/)(16, 17) and the Human Protein Atlas (https://www.proteinatlas.org/)(6), which suggests they are coming from dead cells and debris instead of specific labeling on the cell surface.

*Note: *Mouse* PIEZO1-BBS (cap) is being stably expressed in a *human* cell line; therefore, peptide quantification for PIEZO1 (due to self-labeling) appears lower than would be expected for this type of experiment (only common tryptic peptides between mouse and human PIEZO1 are being counted). Using a mouse proteome search algorithm instead of human for this same dataset, PIEZO1 goes from 4.4% sequence coverage (spectral count 36) up to 25.25% sequence coverage (spectral count 177), indicating that this difference is only due to species.

SI References

1. E. W. Deutsch, *et al.*, The ProteomeXchange consortium at 10 years: 2023 update. *Nucleic Acids Res* **51**, D1539–D1548 (2023).

2. Y. Perez-Riverol, *et al.*, The PRIDE database resources in 2022: a hub for mass spectrometry-based proteomics evidences. *Nucleic Acids Res* **50**, D543–D552 (2022).

3. J. Wu, R. Goyal, J. Grandl, Localized force application reveals mechanically sensitive domains of Piezo1. *Nat Commun* **7**, 12939 (2016).

4. K. H. Loh, *et al.*, Proteomic Analysis of Unbounded Cellular Compartments: Synaptic Clefts. *Cell* **166**, 1295-1307.e21 (2016).

5. E. V. Vinogradova, *et al.*, An Activity-Guided Map of Electrophile-Cysteine Interactions in Primary Human T Cells. *Cell* **182**, 1009-1026.e29 (2020).

6. P. J. Thul, *et al.*, A subcellular map of the human proteome. *Science* **356**, eaal3321 (2017).

7. B. Coste, *et al.*, Piezo1 and Piezo2 are essential components of distinct mechanically activated cation channels. *Science* **330**, 55–60 (2010).

8. A. H. Lewis, J. Grandl, Mechanical sensitivity of Piezo1 ion channels can be tuned by cellular membrane tension. *eLife* **4**, e12088 (2015).

9. A. Spandidos, X. Wang, H. Wang, B. Seed, PrimerBank: a resource of human and mouse PCR primer pairs for gene expression detection and quantification. *Nucleic Acids Research* **38**, D792–D799 (2010).

10. A. Spandidos, *et al.*, A comprehensive collection of experimentally validated primers for Polymerase Chain Reaction quantitation of murine transcript abundance. *BMC Genomics* **9**, 633 (2008).

11. X. Wang, B. Seed, A PCR primer bank for quantitative gene expression analysis. *Nucleic Acids Res* **31**, e154 (2003).

12. Z. Zhou, *et al.*, MyoD-family inhibitor proteins act as auxiliary subunits of Piezo channels. *Science* **381**, 799–804 (2023).

13. C. Wu, X. Jin, G. Tsueng, C. Afrasiabi, A. I. Su, BioGPS: building your own mash-up of gene annotations and expression profiles. *Nucleic Acids Research* **44**, D313–D316 (2016).

14. A. Gschwind, S. Hart, O. M. Fischer, A. Ullrich, TACE cleavage of proamphiregulin regulates GPCR-induced proliferation and motility of cancer cells. *EMBO J* **22**, 2411–2421 (2003).

15. M. S. Warner, *et al.*, A cell surface protein with herpesvirus entry activity (HveB) confers susceptibility to infection by mutants of herpes simplex virus type 1, herpes simplex virus type 2, and pseudorabies virus. *Virology* **246**, 179–189 (1998).

16. M. Ashburner, *et al.*, Gene Ontology: tool for the unification of biology. *Nat Genet* **25**, 25–29 (2000).

17. The Gene Ontology Consortium, *et al.*, The Gene Ontology knowledgebase in 2023. *Genetics* **224**, iyad031 (2023).
